# Alkaloids of *Abuta panurensis* Eichler: *In silico* and *in vitro* study of acetylcholinesterase inhibition, cytotoxic and immunomodulatory activities

**DOI:** 10.1371/journal.pone.0239364

**Published:** 2020-09-29

**Authors:** Rochelly da Silva Mesquita, Andrii Kyrylchuk, Regiane Costa de Oliveira, Ingrity Suelen Costa Sá, Gabriel Coutinho Borges Camargo, Gemilson Soares Pontes, Felipe Moura Araújo da Silva, Rita de Cássia Saraiva Nunomura, Andriy Grafov

**Affiliations:** 1 Department of Chemistry, Federal University of Amazonas (UFAM), Manaus, Amazonas, Brazil; 2 Institute of Organic Chemistry, National Academy of Sciences (NAS), Kyiv, Ukraine; 3 Post-Graduate Program in Hematology, University of the State of Amazonas (UEA), Manaus, Amazonas, Brazil; 4 Laboratory of Virology, National Institute of Amazonian Research (INPA), Manaus, Amazonas, Brazil; 5 Analytical Center –Multidisciplinary Support Center (CAM), Federal University of Amazonas (UFAM), Manaus, Amazonas, Brazil; 6 Department of Chemistry, University of Helsinki, Helsinki, Finland; UMR-S1134, INSERM, Université Paris Diderot, INTS, FRANCE

## Abstract

Natural products obtained from species of the genus *Abuta* (Menispermaceae) are known as ethnobotanicals that are attracting increasing attention due to a wide range of their pharmacological properties. In this study, the alkaloids stepharine and 5-*N-*methylmaytenine were first isolated from branches of *Abuta panurensis* Eichler, an endemic species from the Amazonian rainforest. Structure of the compounds was elucidated by a combination of 1D and 2D NMR spectroscopic and MS and HRMS spectrometric techniques. Interaction of the above-mentioned alkaloids with acetylcholinesterase enzyme and interleukins IL-6 and IL-8 was investigated *in silico* by molecular docking. The molecules under investigation were able to bind effectively with the active sites of the AChE enzyme, IL-6, and IL-8 showing affinity towards the proteins. Along with the theoretical study, acetylcholinesterase enzyme inhibition, cytotoxic, and immunomodulatory activity of the compounds were assessed by *in vitro* assays. The data obtained *in silico* corroborate the results of AChE enzyme inhibition, the IC_50_ values of 61.24μM for stepharine and 19.55μM for 5-*N*-methylmaytenine were found. The compounds showed cytotoxic activity against two tumor cell lines (K562 and U937) with IC_50_ values ranging from 11.77 μM to 28.48 μM. The *in vitro* assays revealed that both alkaloids were non-toxic to Vero and human PBMC cells. As for the immunomodulatory activity, both compounds inhibited the production of IL-6 at similar levels. Stepharine inhibited considerably the production of IL-8 in comparison to 5-*N*-methylmaytenine, which showed a dose dependent action (inhibitory at the IC_50_ dose, and stimulatory at the twofold IC_50_ one). Such a behavior may possibly be explained by different binding modes of the alkaloids to the interleukin structural fragments. Occurrence of the polyamine alkaloid 5-*N*-methylmaytenine was reported for the first time for the Menispermaceae family, as well as the presence of stepharine in *A*. *panurensis*.

## Introduction

Menispermaceae family has a wide geographic distribution, mainly in tropical and subtropical regions of the world; its name is related to a crescent moon shape of the seeds [1, 2]. The genus *Abuta* is native to tropical Central and South America, where it is represented by more than 30 species. Some of them have been used by indigenous people to prepare curare, alkaloid-containing arrow and dart head poisons that paralyze the prey [1, 3].

As a result, the Menispermaceae family have been in a focus of rising interest to study several classes of secondary plant metabolites, including alkaloids; which are rather abundant constituents [4–11]. Several classes of alkaloids were isolated from *Abuta*, those include: isoquinoline [12, 13], benzylisoquinoline [12, 14], benzyltetraisoquinoline [15], bisbenzyltetraisoquinoline [15–17], aporphine [18], and proaporphine [12, 15] derivatives; as well as other less frequent ones such as tropolone-isoquinoline, azafluoranthene, and benzazepine alkaloids [19, 20]. Those alkaloids reveal a wide range of pharmacological activities including muscle relaxant [1], antiplasmodial [14], inhibitory for acetylcholinesterase (AChE) and butyrylacetylcholinesterase enzymes [17, 21, 22], cytotoxic [19, 23, 24], and immunomodulatory [25].

A stepharine is one of the most representative proaporphine alkaloids in Menispermaceae family; it was first identified in the genus *Stephania* [26]. Proaporphine alkaloids are principally known for their potential to inhibit reversibly the acetylcholinesterase enzyme [27, 28]. Effectiveness and anti-AChE potential of the stepharine sulfate salt (stephaglabine) was reported for the treatment of traumatic and postoperative injuries of the peripheral nervous system and confirmed by a clinical study [29]. However, a pharmaceutical potential of stepharine is much wider according to the literature reports [30, 31]. It showed a cytotoxic activity against two human lung cancer cell lines [32], as well as a weak antifungal potential and DNA- damaging activity [30]. Therefore, both isolation of stepharine from plants cell cultures and several synthetic procedures were developed to satisfy a growing need for the medicinal use of the compound [31, 33].

Polyamine alkaloids (the derivatives of putrescine, spermidine, spermine, and cadaverine) are metabolites that occur widely in angiosperm plants [34–37], but are practically absent in sterile ones [35, 37–42]. Those compounds may also be isolated from other natural sources [43], particularly from fungi [44–48]. The polyamine alkaloids are not common for the Menispermaceae family, they were reported only for a *Cissampelos* genus [37, 49]. Cinnamoyl derivatives of polyamine alkaloids inhibit the AChE and α-glucosidase [38, 50, 51]. Alongside, they also inhibited cancer cell growth [46, 48, 52] and revealed an antiviral activity [53]. In the Amazon region, a polyamine alkaloid *N*,*N’*-di-*E*-cinnamoylspermidine or maytenine was isolated from *Maytenus krukovii (Maytenus chuchuhuasha)* trees (Celastraceae) [46, 54]. Several studies have stimulated the development of synthetic approach to that class of polyamides [46, 55–58] including the maytenine synthesis [59].

In the present study, 5-*N*-methylmaytenine (**1**) and the stepharine (**2**) were isolated for the first time from *A*. *panurensis*. This is also the first report on the occurrence of (**1**) in Menispermaceae family. Interactions of the alkaloids in question with acetylcholinesterase (AChE) enzyme and cytokines IL-6 and IL-8 were investigated *in silico* by molecular docking. The potential of (**1**) and (**2**) as AChE inhibitors, antitumor and immunomodulatory agents was demonstrated by *in vitro* studies.

## Materials and methods

### Chemicals

Reagents and HPLC-grade solvents were purchased from Tedia Company (Fairfield, OH, USA) and Sigma-Aldrich and used as supplied. P.A. (Nuclear) grade solvents were purified by standard procedures used in natural products chemistry. An ultrahigh-purity water was obtained by Milli Q system (Millipore, Bedford, MA, USA).

### Plant material

The authors declare that a specific permission from the National Institute of Amazonian Research (INPA) was required to collect plant material. The authors got the permission No. 35/12 of 02.12.2017 and confirm that the study did not involve endangered or protected species. *A*. *panurensis* plant material was collected at the Adolpho Ducke Forest Reserve, 26 km along the AM-010 highway from the city of Manaus, the State of Amazonas, Brazil. The species under investigation had been identified by the taxonomist L.S.Mergulhão. The voucher specimens were deposited in the Herbarium of the National Institute of Amazonian Research (INPA) under the voucher no 279373. The access to genetic heritage was registered at the National System of Genetic Heritage and Associated Traditional Knowledge Management (SisGen, Brazil) under the code number A9CC956.

The branches collected were dried at room temperature (ca. 20°C) for 10 days. Subsequently, the vegetal material (1.4 kg of branches) was crushed in a knife mill and stored in a refrigerator until use.

### Extraction

Dried and crushed plant material was subjected to an acid-base extraction [60]. The crushed branches (300g) were macerated with a 10% solution of NH_4_OH (2L) and CH_2_Cl_2_ (2L) at room temperature (20°C) for 72h, the material was stirred every 24 hours. The organic phase (1.5L) was transferred to a separatory funnel with a 10% solution of acetic acid (2L) and stirred manually. Then, the acidic aqueous phase was transferred to another vessel and the pH was adjusted to 10 using NH_4_OH and extracted with CH_2_Cl_2_ (2 × 300mL). The CH_2_Cl_2_ phase was separated, concentrated on a rotary evaporator under reduced pressure, and dried with anhydrous sodium sulfate, resulting in the alkaloid fraction (280mg).

### LC-APCI-MS analysis

LC-APCI-MS analyzes were performed on an Acella chromatograph (Thermo Scientific); coupled to a triple-quadrupole mass spectrometer model TSQ Quantum Acess^®^ (Thermo Scientific), equipped with an Atmospheric Pressure Chemical Ionization (APCI) source, operated in positive mode with monitoring in the range of *m/z* 100–800. The mass spectrometer was equipped with Surveyor LC Pump Plus, Surveyor Autosampler Plus, Rheodyne injection valve (25μL), Luna C18 column (150 × 4.60mm, 5μm) (Phenomenex–Torrance, CA, USA), operating simultaneously with Surveyor PDA Plus diode array detector (DAD). The mobile phase was composed of B (methanol) and A (formic acid 1% v/v in H_2_O) with a linear elution gradient: 0–20 min 20–80% B, 20–35 min 80% B, 35–45 min 20–80% B. The flow rate of the mobile phase was 1 mL/min and the injection volume was 10μL. The DAD detector was set up for monitoring between 200-400nm. The spectra were processed using an Xcalibur software (version 2.2).

### Semi-preparative HPLC analysis

Isolation of the alkaloids was performed on a semi-preparative scale on a Shimadzu chromatograph composed of a CBM-20A communication module, SPD-20AV UV detector, DGU-20A5 degasser, LC-6AD pump, 200 μL Rheodyne injection valve, and Luna C18 column (250 x 15.00mm, 5μm) (Phenomenex–Torrance, CA, USA) with a flow rate of 3 mL/min. The mobile phase was composed of B (methanol) and A (formic acid 1% v/v in H_2_O), with a linear elution gradient: 0–20 min 20–80% B, 20–35 min 80% B, 35–45 min 20–80% B. The UV detector was set to monitoring at 260nm and 280nm. Fractions containing 5-*N*-methylmaytenine (11.2mg—**1**) and stepharine (22.1mg—**2**) were collected and analyzed by high-resolution mass spectrometry (HRMS) and NMR spectroscopy.

### High resolution mass spectrometry

HRMS analyses were performed on a Shimadzu chromatograph composed of a CBM-20A communication module, a SPD-20AV UV detector, a LC-20AD pump, a SIL-20A HT autosampler (200μL), a CTO-20A oven, and a Luna PFP column (150 x 2mm, 100A); coupled to a Bruker microTOF-QII mass spectrometer, equipped with an Atmospheric Pressure Chemical Ionization (APCI) source, operated in a positive mode. The instrument parameters were as follows: capillary voltage, 4500V; nebulizer pressure (N_2_), 4.0 bar; dry gas flow (N_2_), 8L/min; dry heater temperature, 200°C; with a monitoring in the range of *m/z* 100–800 Da. The mobile phase was composed of B (formic acid 0.1% v/v in methanol) and A (formic acid 0.1% v/v in H_2_O) with a linear elution gradient as follows: 0–2 min 20–80% B; 2–42 min 100% B. The flow rate of the mobile phase was set to 0.2 mL/min and the injection volume was 10μL. The UV detector was set up for monitoring between 254nm and 330nm. The spectra were processed using a Bruker Compass Data Analysis software (version 4.2).

### 1D and 2D NMR spectroscopy

NMR spectra were recorded on a Bruker Avance IIIHD spectrometer, 500.13 MHz for ^1^H and 125.0 MHz for ^13^C operated at a magnetic field strength of 11.7 Tesla, equipped with a 5 mm direct detection PA BBO BBF HD-05-Z SP Intelligent probe incorporating Z-axis gradient coil, capable of providing gradient amplitudes up to 50 G/cm. Shigemi’s 5.0 mm NMR tubes were used. For structural elucidation, the samples of 5-*N*-methylmaytenine and stepharine were solubilized in 600 μL of DMSO-d_6_ (δ_H_ 2.50, δ_C_ 39.9) and CD_3_OD (δ_H_ 3.34, δ_C_ 49.8), respectively. The acquisition of ^1^H, ^13^C, DEPT 135, COSY, HSQC, and HMBC spectra was performed using standard Bruker pulse sequences. The analysis based on ^1^H NMR data was performed by solubilizing 10.0 mg of the 5-*N*-methylmaytenine in 550 μL of DMSO-d_6_ with 50 μL of TMS (0.5 mM, 98%, Tokyo Chemical Industry) and 15.0 mg of the stepharine in 550 μL of CD_3_OD with 50 μL of TMS (0.5 mM, 98%, Tokyo Chemical Industry) at 25 °C. Acquisition of 5-*N*-methylmaytenine and stepharine spectra was performed using the zg30 pulse sequence with water signal suppression, data points of the 64 kB time domain, 10 kHz spectral width, 1.00 second relaxation delay (D1), 3.27 second acquisition time (AQ), 32 scan numbers with DS of 2, decomposition resolution of 0.31 Hz, a constant receiver gain at 161 (5-*N*-methylmaytenine) and 181 (stepharine) with displacement frequency set at 2,425.23 Hz, PLW1 of 20.3 W. The calibration pulse (P1 9.400 μs to 5-*N*-methylmaytenine and P1 10.300 μs to stepharine) with PLW9 were of 7.183×10^−5^ W (5-*N*-methylmaytenine) and 8.6243×10^−5^ W (stepharine). Data were processed using Bruker^®^ Topspin 4.0.6 software.

### Molecular docking calculations

#### Ligand structures preparation

Ligand structures were built manually and preliminary optimized in a classic molecular mechanics software. In order to use equilibrium ligand structures, their geometry optimizations were conducted using a semi-empirical PM7 level [61] within the MOPAC program [62]. Ligand structure files for docking were prepared using AutoDock Tools [63, 64]. Default settings for the detection of rotatable bonds were used.

#### Protein files preparation

X-ray structures of the proteins under investigation (AChE, PDB ID: 6H12; IL-6, PDB ID: 4NI7 and IL-8, PDB ID: 3IL8) were obtained from the RCSB Protein Data Bank. *Missing residues*. Modeling of missing residues in the protein structures was performed using Modeller web-service [65]. Obtained structures that do not possess serious structural issues were selected for docking studies. Clashes or contacts between the side chains of the produced structures were resolved by minimization routine implemented in UCSF Chimera [66]. *Water molecules* are commonly found in the XRD structures of proteins and can have substantial impact on the docking affinities. Selection of water molecules that could be important in docking was performed according to the distance criterion. All water molecules farther than 3.3 Å from the H-bond donors and acceptors in the protein structures were removed. Protonation of the oxygen atoms in water molecules was made in UCSF Chimera followed by minimization of the hydrogen positions.

Binding sites of the proteins were identified using Discovery Studio Visualizer [67]. AutoDock Tools [63,64] were used for the preparation of Structure files for docking.

#### Docking runs

Docking studies were performed with AutoDock Vina program [68]. The protein structures stayed rigid during the docking in all cases. Each run generated nine binding poses. Exhaustiveness parameter of 50 was used for the medium search space sizes, e.g. for the binding pocket in AChE. In the case of larger search spaces (IL-6, IL-8) exhaustiveness of 500 was used. Since the success of a docking run depends on a random seed, which is defined at the beginning of the run and does not change during it; we have performed three docking runs for each protein-ligand pair and search space. The best docking poses found were similar between the runs in most cases, showing that the chosen parameters provided exhaustive search of the conformational space. *Missing residues*. Selected protein structures from Modeller service were used for the estimation of binding to the regions that were missing in the initial XRD structures. Search spaces that include the modeled parts of the protein were chosen. *Water molecules*. It is known that inclusion of all water molecules in the docking run can lead to erroneous results [69]. Therefore, trial docking runs were performed with an inclusion of one of the crystallization water molecules found in the XRD structures at a time. The water molecules were fixed during the docking run. The search space either covered the defined binding site of the protein or was centered on the water molecule and had a size of 20×20×20 Å.

Protein structure images were obtained using Discovery Studio Visualizer [67], PyMOL [70], and UCSF Chimera [66]. Detailed docking parameters are collected in the S1 File.

### Acetylcholinesterase inhibition assay

Acetylcholinesterase enzyme from an electric ray *Tetronarce californica* (Sigma-Aldrich, USA) was used for the experiments. The *in vitro* AChE inhibition assay was performed in 96-well microplates according to the methodology proposed by Ellman *et al*. [71] (1961) and Senol *et al*. [72] (2015) with some modifications. The alkaloids were tested at concentrations of 2.8; 5.6; 11.2; 22.5; 45.0 and 90.0 μg/mL. Initially, 20 μL of each sample from the stock solution (1 mg/mL) were added and serial dilutions were performed. Then, 150 μL of a sodium phosphate buffer pH = 8 (0.1 mM), 20 μL of 5,5´-dithio-bis(2- nitrobenzoic)acid (DTNB, 0.0025 M), and 20 μL of the acetylcholinesterase enzyme (1 U/mL) were added subsequently to each well at 25 °C and left for 15 minutes. The reaction was initiated by addition of 10 μL of acetylcholine iodide (AChI) (0.1 M). Neostigmine (0.28–9.0 μg/mL) was used as a positive control.

A thiocholine formed by the enzymatic hydrolysis of the AChI, reacts with the DTNB giving rise to yellow 5-mercapto-2-nitrobenzoate anion. Concentration of the latter in each well was measured as absorbance at 405 nm using a 96-well microplate reader spectrophotometer (Biotek model ELX800). The transformation was monitored for 30 min at 5 min intervals. Inhibition curve was plotted as the inhibition percent *vs* concentration. All assays were performed in triplicate.

### Cytotoxicity assay

The cytotoxicity of the alkaloids to different cell lines was evaluated by MTT (3-(4,5-dimethylthiazol-2-yl)-2,5-diphenyltetrazolium bromide) cell proliferation assay. The following cell lines were used for the evaluation: K562 (human chronic myelogenous leukemia, ATCC^®^ CCL-243^™^), U937 (human histiocytic lymphoma, ATCC^®^ CRL-1593.2^™^), HL60 (human acute promyelocytic leukemia, ATCC^®^ CCL-240^™^), Vero (kidney epithelial cells extracted from an African green monkey *Chlorocebus* sp., ATCC^®^ CCL-81^™^), and human peripheral blood mononuclear cells (PBMC) from healthy blood donors. The cell lines K562, HL60, and U937 were kindly donated by Prof. S.O. Saad (Hematology and Hemotherapy Center at the National Institute of Blood Science and Technology of the University of Campinas, Campinas, São Paulo, Brazil). The Vero cells were kindly donated by Prof. F.Naveca (Laboratory of Infectious Disease Ecology in the Amazon, L. and M.Deane Institute, FIOCRUZ, Manaus, Amazonas, Brazil). The cells were cultured in 96-well plates. An amount of 2×10^4^ cells was seeded into each well containing 0.2 mL of a RPMI medium supplemented with 10% FBS, penicillin-streptomycin and fungizone, in an atmosphere of 5% CO_2_ at 37° C for 24 hours. After a formation of sub-confluent monolayer, the cells were treated with different concentrations of the alkaloids (12 … 100 μg/mL) and incubated again at the same conditions for 24, 48, and 72 hours. Sterile PBS and DMSO 100% were used as negative and positive controls, respectively. Subsequently, the medium was removed from all wells and 10 μL of the MTT (5 mg/mL in sterile PBS) diluted in 100 μL of a DMEM medium (without phenol red to avoid misinterpretation) was added into the wells and incubated for 4 hours at the same conditions mentioned above. After that, the MTT was removed and 50 μL of the MTT lysis buffer were added to each well. The plate was homogenized gently to dissolve the formazan crystals and incubated for 10 minutes at 37 °C. Optical densities of the samples at wavelength of 570 nm were measured using a microplate reader. The relative viability of the cells was estimated using the following equation:

$$\frac{\text{A}570\;\text{of}\;\text{treated}\;\text{sample}}{\text{A}570\;\text{of}\;\text{untreated}\;\text{sample}}\times 100,$$

where A570 is the absorbance at 570nm. All assays were done in triplicate.

### Immunological assay

To analyze the immunomodulatory potential, human peripheral blood mononuclear cells (PBMCs) obtained from healthy blood donor candidates were cultured in RPMI-1640 medium in a 96-well plate and incubated for 24h with 5-*N*-methylmaytenine (12.5 μM and 25 μM) or stepharine (28.48 μM and 57.0 μM). After the incubation, the supernatants were collected for cytokine assays. The supernatant of untreated PBMC cells was used as control. The concentrations of IL-6 and IL-8 cytokines were evaluated by commercially available enzyme-linked immunosorbent assay (ELISA) kits (Boster, Pleasanton, CA, USA). The results were normalized for protein levels contained in each sample and were expressed in pg/mg of total protein. The assays were repeated in triplicate for each individual sample using untreated cells as negative control. This study was approved by the Committee for Ethics in Research on Human Beings of the HEMOAM (approval number: 3.138.343).

### Statistical analysis

Statistical analysis was performed with GraphPad Prism 7.0^®^ software using Student’s *t*-test and ANOVA. A probability value of less than 0.05 was chosen as a statistical significance criterion. Throughout the text, the asterisks correspond to the following probability values: * means *p*<0.01; ** means *p*< 0.001; and *** means *p*<0.0001, when compared to the negative control (untreated cells). The half maximum inhibitory concentration IC_50_ values were calculated using nonlinear regression.

## Results

### Spectral data

5-*N*-methylmaytenine (**1**) was isolated as a light yellow amorphous solid (11.2 mg). ^1^H NMR (500 MHz, DMSO d_6_, TMS): δ 8.12 (t; 2H; *J* = 5.5 Hz, 1 and 10-NH), δ 7.56 (m, 2H, 5´-H and 5´´-H or 9´-H and 9´´-H), δ 7.54 (m, 2H, 9´-H and 9´´-H or 5´-H and 5´´-H), δ 7.55 (m, 2H, 6´-H, 6´´-H, 8´-H, 8´´-H), δ 7.40 (m, 2H, 7´-H and 7´´-H), δ 7.42 (m, 1H, 3´-H or 3´´-H), δ 7.38 (m, 1H, 3´´-H or 3´-H), δ 6.63 (d, 1H, 2.4 Hz, 2´-H or 2´´-H), δ 6.60 (d, 1H, 2.4 Hz, 2´´-H or 2´-H), δ 3.20 (m, 2H, 2-H), δ 3.18 (m, 2H, 9-H), δ 2.35 (m, 2H, 4-H), δ 2.32 (m, 2H, 6-H), δ 2.16 (s, 3H, 5-NCH_3_), δ 1.61 (m, 2H, 3-H), δ 1.47 (m, 2H, 8-H), δ 1.45 (m, 2H, 7-H). ^13^C NMR (125 MHz, DMSO d_6_, TMS): δ 165.32; 165.27; 135.43; 127.98; 127.94, 129.39; 129.83; 138.85; 138.88; 122.79; 122.83; 55.26; 57.12; 42.09; 37.58; 39.07; 27.28; 24.53; 27.48 ppm. (S1 Table in S1 File). MS (APCI+) *m/z* 420 [M+H]^+^: 202, 188, and 131. HRMS *m/z* 420.2669 (calc. for C_26_H_34_N_3_O_2_ *m/z* 420.2646, Δ_*m/z* theor._ = -5.6 ppm).

Stepharine (**2**) was isolated as a light brownish amorphous solid (22.1mg). ^1^H NMR (500 MHz, CD_3_OD, TMS): δ 7.01 (dd, 1H, 3 and 10Hz, 12-H), δ 7.16 (dd, 1H, 3 and 10Hz, 8-H), δ 6.89 (s, 1H, 3-H), δ 6.41(dd, 1H, 1.8 and 10Hz, 11-H), δ 6.29 (dd, 1H, 1.8 and 10Hz, 9-H), δ 4.72 (m, 1H, 6a-H), δ 3.82 (s, 3H, 2-OCH_3_), δ 3.70 (ddd, 1H, 1.5, 6.3 and 13Hz, 5-H), δ 3.61 (s, 3H, 1-OCH_3_), δ 2.52 (dd, 1H, 6.6 and 12 Hz, 7-H), δ 2.42 (dd, 1H, 10.5 and 12Hz, 7`-H), δ 3.44 (ddd, 1H, 6.3, 11 and 13Hz, 5-H), δ 3,02 and δ 3,00 (m, 2H, 4-H), δ 1.95 (s, 1H, NH) ppm. ^13^C NMR by HSQC (125 MHz, CD_3_OD, TMS): δ 153.63; 150.28; 112.15; 127.81; 126.74; 56.48; 55.37; 43.71; 59.95, 44.90; 43.71; 23.5 ppm. (S2 Table in S1 File). MS (APCI+) *m/z* 298 [M+H]^+^: 281, 266, 250, 235, 161. HRMS *m/z* 298.1461 (calc. for C_18_H_20_NO_3_ *m/z* 298.1438, Δ_*m/z* theor._ = -7.9 ppm).

### Binding with acetylcholinesterase of *Tetronarce californica* (TcAChE)

X-ray structure of the AChE from the electric ray *T*. *californica* was found in the RCSB Protein Data Bank under 6H12 code [73]. The crystal structure retrieved from the Data Bank represents the AChE adduct with a functionalized urea ligand. In order to verify robustness of the method, preliminary docking studies were conducted using the ligand structures obtained from the experimental XRD data. The best affinity of the ligand without the inclusion of water molecules reached -15.3 kcal/mol with a RMSD value of 4.03 Å compared to the ligand pose found in the crystal structure. Relatively high RMSD value can be explained by substantial internal mobility of the ligand molecule. Inclusion of 18 crystallization water molecules increased the affinity value to -14.0 kcal/mol with comparable RMSD. In the case of fully hydrated pocket, many binding poses were found with similar affinity values, ranging from -13 to -14 kcal/mol. Some of those conformations showed better RMSD values reaching 2.2 Å. Screening of the water molecules failed to achieve interaction energies lower than those obtained for the empty binding pocket. Therefore, water molecules do not seem to play a substantial role in the binding of the urea ligand. Still, there was a possibility that hydration could be important for interactions with stepharine or 5-*N*-methylmaytenine. Therefore, it was considered for those ligands as well.

**Fig 1:**
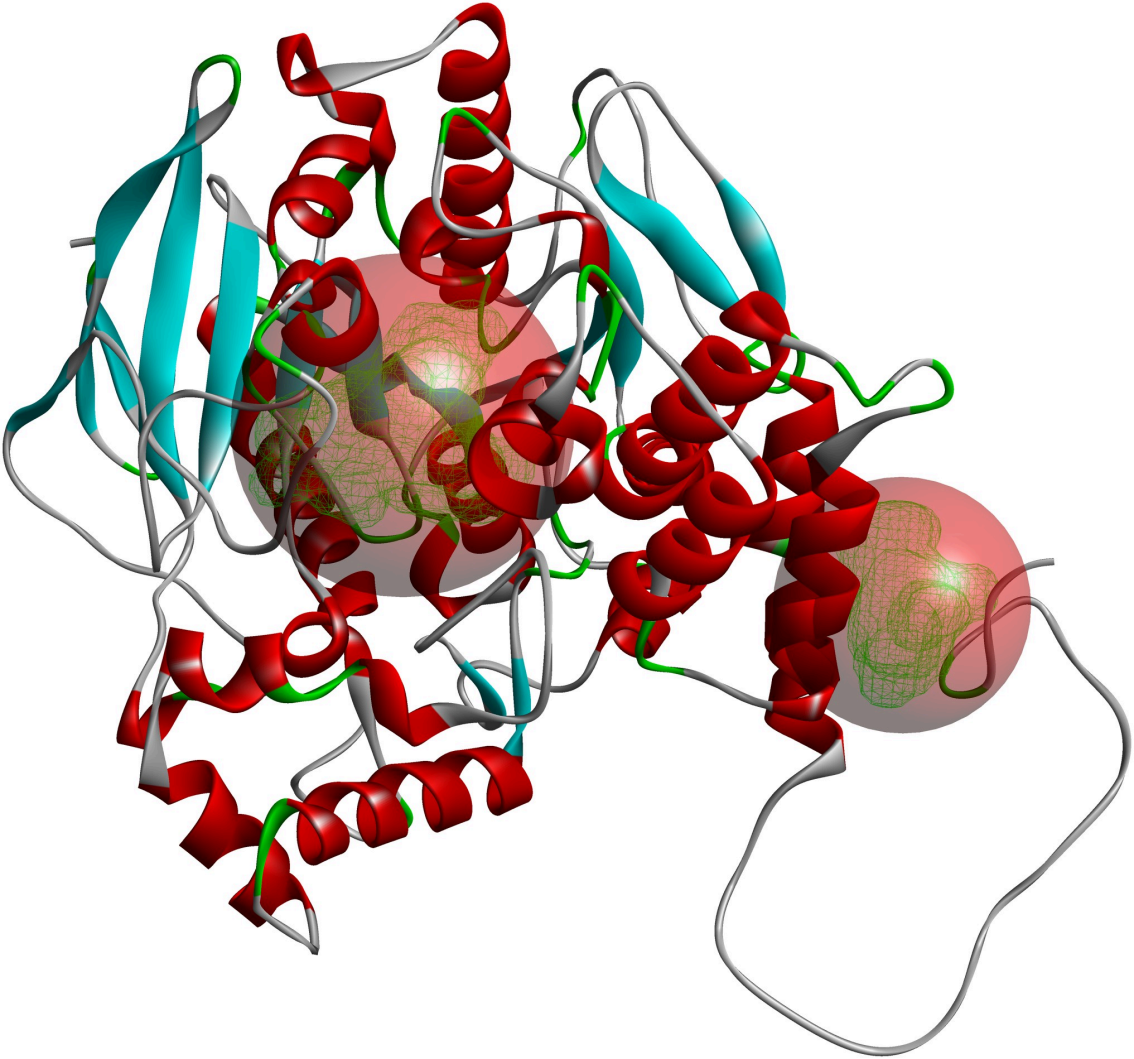
Two major binding sites found for AChE. Other, smaller sites are not shown.

Two major binding sites were identified in the AChE structure–the main active site and the region near the modeled C-terminal loop (Fig 1). Consequently, we have conducted docking studies of these sites and a rear region that can possess smaller pockets (S32–S37 Figs in S1 File and the accompanying information).

Neostigmine is known as an efficient AChE inhibitor that was used as positive control in this study. According to the neostigmine docking results, the best affinity value was achieved for the main cavity of the enzyme and reaches -7.6 kcal/mol. The binding energies for the other sites were weaker by ca. 2 kcal/mol. Inclusion of water molecules did not produce any pronounced effect on the affinities and changed them by ca. 0.5 kcal/mol (S31 Fig in S1 File and the accompanying information).

5-*N*-methylmaytenine (**1**) preferably binds to the AChE active site with the affinity of -10.5 kcal/mol. Stepharine (**2**) fits well inside the AChE active site with the predicted binding affinity of -10.3 kcal/mol. Affinities of both ligands to the other sites were weaker by 2 …4 kcal/mol. Explicit inclusion of water molecules into the binding cavity had little effect on the binding strengths (S31 Fig in S1 File and the accompanying information).

### Binding with interleukin-6

**Fig 2:**
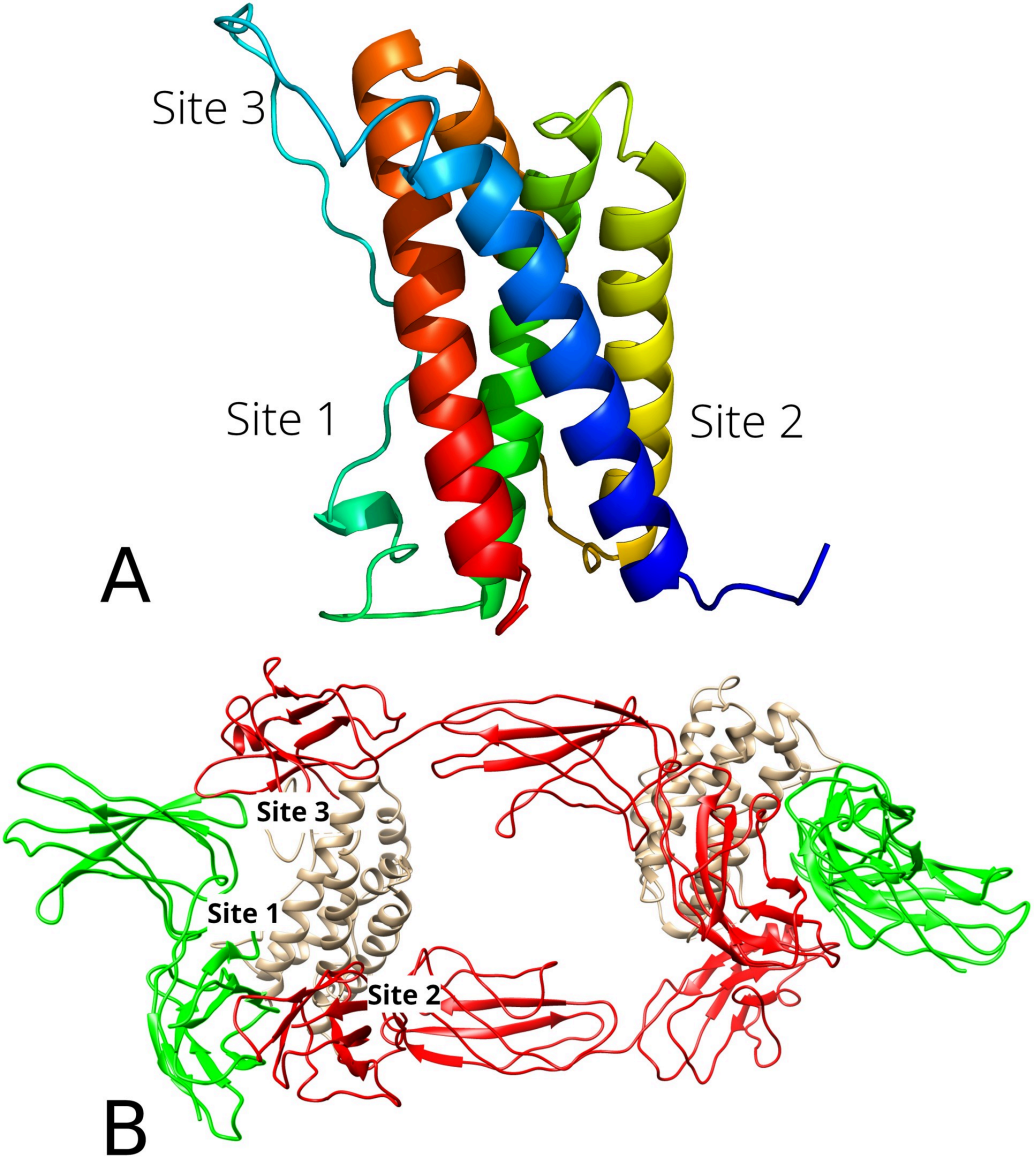
Binding sites of the IL-6 according to [74] (A) and IL-6 receptor complex 1P9M (B). In Fig 2B, the IL-6 structures are beige, the gp130 is marked red, and the IL-6r is green.

X-ray structure of human interleukin-6 (PDB ID: 4NI7) was used for the docking studies [74]. The structure contains a co-crystallized nucleic acid moiety that was removed prior to docking. During the signaling event, the IL-6 associates with the IL-6 receptor (IL-6r) and forms a complex. Then, the second receptor protein, *viz*. the gp130 glycoprotein, binds to the complex giving rise to a dimer; and the signaling is initiated [74–76]. X-ray structure of the interleukin-receptor complex (Fig 2B) was retrieved from the RCSB PDB (ID: 1P9M) [76].

According to the original paper [74], the IL-6 molecule possesses three binding sites, where the interactions with the receptor proteins and the second IL-6 molecule occur (Fig 2A). IL-6 –IL-6r interaction takes place at the Site 1, whereas the IL-6 interacts with the gp130 at the Sites 2 and 3. The IL-6 molecule does not have an apparent binding pocket. Four small binding pockets at the Sites 1 and 2 and several smaller pockets at the upper rim of the protein were identified. Since the pocket volume has never exceed 42 Å^3^, the entire protein surface was considered for the docking calculations.

The strongest affinity of -7.9 kcal/mol was found for **1**, when the ligand interacted with two helices and a modeled loop at the IL-6 upper rim. Several other binding modes employed an interaction with a side surface of the two helices, including a π-stacking with both 5-*N*-methylmaytenine phenyl rings as well. Water molecules had also been screened for possible interactions with the docked ligand, but no significant effect was found (p 45 of the S1 File).

Stepharine (**2**) binds to the IL-6 with the affinity of -6.9 kcal/mol at the same site as 5-*N*-methylmaytenine. Again, water molecules did not provide any additional binding strength (p 45 of the S1 File).

### Interleukin-8

**Fig 3:**
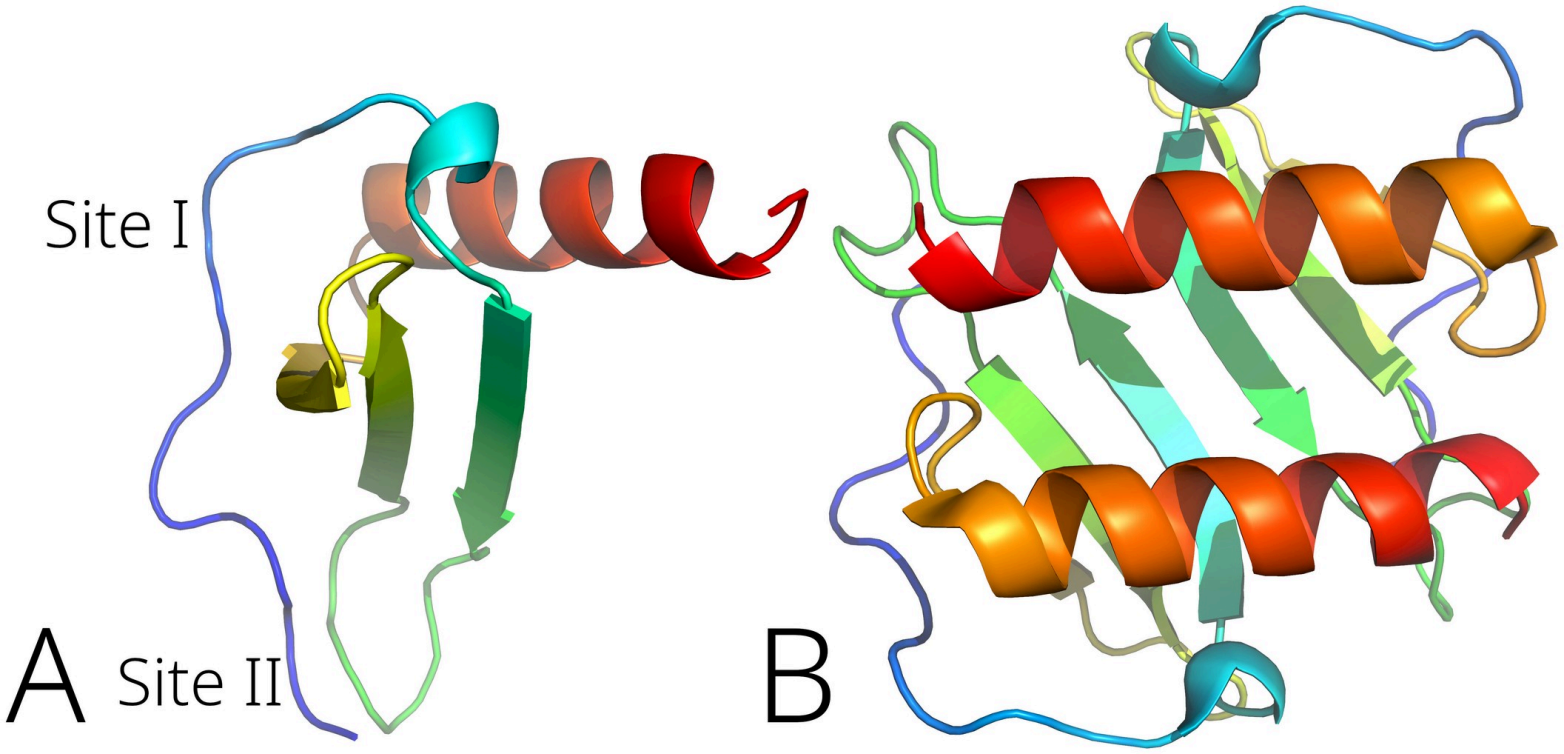
Binding sites of the IL-8 monomer according to [78–80] (A) and IL-8 dimer structure (B).

The crystal structure of the interleukin-8 was retrieved from the RCSB protein data bank (PDB ID: 3IL8) [77]. It is known that IL-8 interaction with the appropriate receptors (CXCR1 and CXCR2) involves two sites close to the N-terminus (Fig 3) [78–80]. The N-loop designated as Site I includes the residues from Ser14 to Lys20. The Site II is comprised of Glu4-Leu5-Arg6 (‘‘ELR”) residue sequence. During the signaling event, the Site I interacts with the receptor N-terminal residues while the Site II is involved in the interaction with the receptor extracellular residues [78]. Two other important regions of the protein structure comprise lateral sides of the α-helix and the β-strand involved in the IL-8 dimer formation.

No binding sites were identified by the Discovery Studio routines. Both the IL-8 monomer and dimer were employed in the docking studies. Due to a moderate size of the interleukin molecule, the entire surface of the protein was included into the search space. 5-*N*-methylmaytenine (**1**) interacts with the IL-8 monomer and dimer with the affinities of -6.9 and -7.0 kcal/mol, respectively. Stepharine (**2**) interacts with the monomeric and dimeric forms of the IL-8 with the similar affinity of -5.9 kcal/mol. Screening of the water molecules did not improve the binding strengths (S38 and S39 Figs in S1 File and accompanying information).

### Acetylcholinesterase inhibition assay

The inhibitory activity of 5-*N*-methylmaytenine and stepharine towards the AChE enzyme showed the inhibition percentage of 78.01 ± 0.09% and, 74.58 ± 0.03% respectively. The inhibition percentage of 93.04 ± 0.03% was found for neostigmine, the efficient short-term reversible inhibitor of the AChE enzyme, used as a positive control. The compounds isolated from *A*. *panurensis* revealed promising IC_50_ values of 19.55μM for 5-*N*-methylmaytenine and 61.24μM for stepharine, the IC_50_ of 3.72μM was observed for neostigmine (Table 1).

**Table 1:** Cytotoxic and AChE inhibitory activity of (1) and (2).

Compounds	IC_50_ (μM)			
	Cell lines			AChE
	K562	U937	HL60	
5-*N*-methylmaytenine	12.5	11.77	>100	19.55
Stepharine	28.48	19.97	>100	61.24
Neostigmine (positive control, AChE)	n/a	n/a	n/a	3.72

n/a–not applicable

### Cytotoxicity assay

The compounds were screened for anticancer activity against three cancerous cell lines K562 (human chronic myelogenous leukemia), U937 (human histiocytic lymphoma), and HL60 (human acute promyelocytic leukemia). The compounds demonstrated a good antiproliferative activity against the former two cell lines investigated. None of the compounds showed cytotoxicity against normal cells of the Vero line and human PBMC. The results for each compound are summarized in the Table 1.

### Immunological assay

The levels of cytokines IL-6 and IL-8 were assessed by ELISA in the supernatant of human PBMC cells treated with 5-*N*-methylmaytenine and stepharine. Our results demonstrated that both 5-*N*-methylmaytenine and stepharine inhibited the IL-6 production in human PBMC after 24 hours treatment in levels statistically significant (*p* = 0.0001), when compared to the untreated PBMC. However, only stepharine induced significant (*p* = 0.01) decrease in the IL-8 levels, when compared to the control. It is interesting to note that the IL-8 levels became up-regulated in comparison to the control, when the concentration of 5-*N*-methylmaytenine was doubled to 2×IC_50_ (Table 1). The results suggest that both **1** and **2** induce the anti-inflammatory modulation, but the effect on the IL-8 expression is dose-dependent in the former case.

## Discussion

### Spectral data interpretation

**Fig 4:**
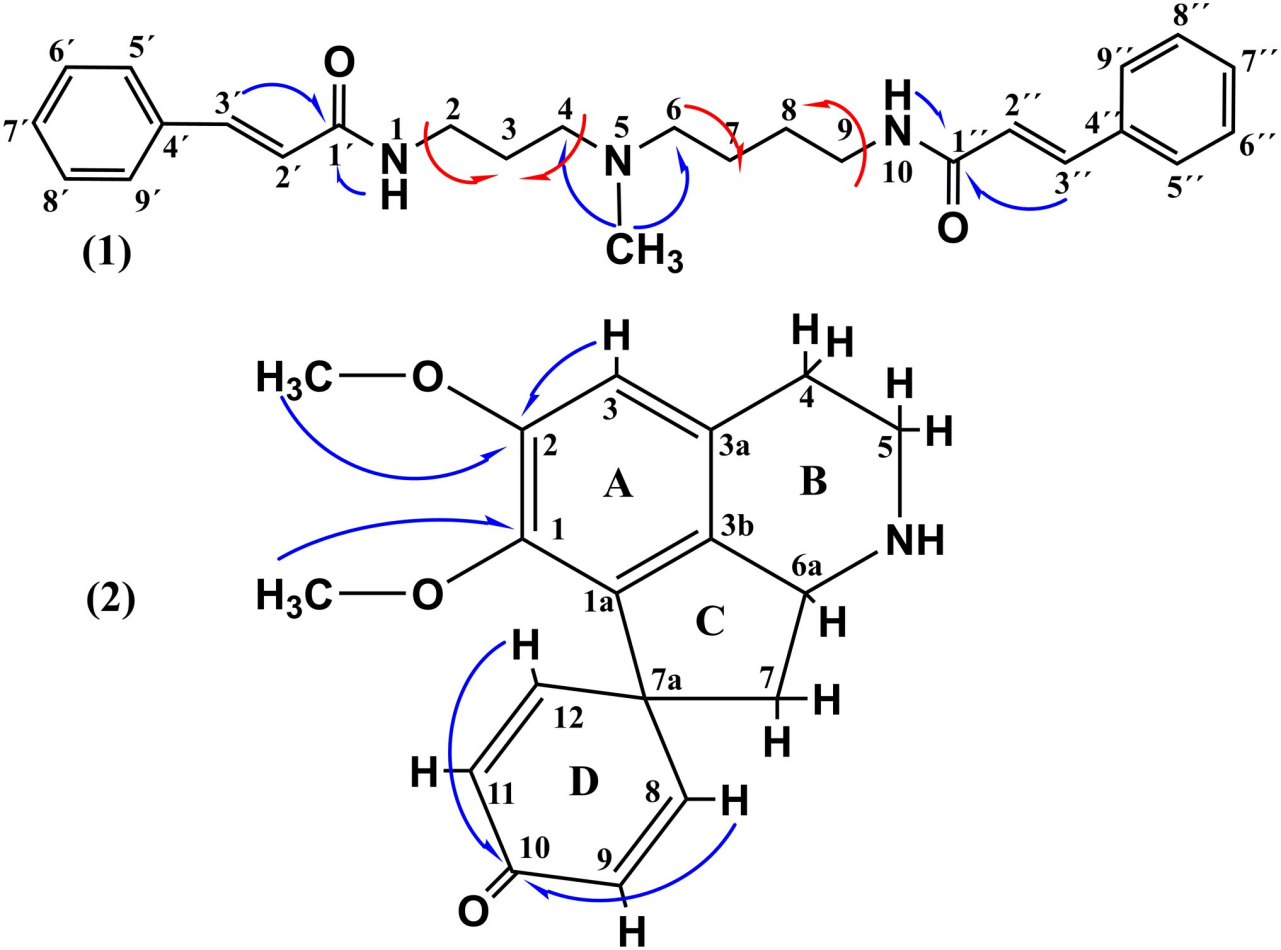
5-*N*-methylmaytenine (1) and stepharine (2). Principal couplings are shown with arrows: HMBC–blue, COSY–red.

Structural formulas of the alkaloids with numbering of atoms are shown in the Fig 4. The MS and NMR spectra of both alkaloids are found in the S1 File.

Molecular formula of the compound (**1**) was determined by HRMS in the positive mode as C_26_H_34_N_3_O_2_. The ^1^H NMR data revealed (Fig 4) the presence of two dehydroxylated cinnamic acid moieties characterized by signals of unsubstituted aromatic rings at δ_H_ 7.40 (m, H-7´and H-7´´) and δ_H_ 7.54–7.56 (m, H-5´; H-5´´; H-6´; H-6´´; H-8´; H-8´´; H-9´; H-9´´), as well as the signals of methylene protons at δ_H_ 7.38 (m, H-3´´ or H-3´), δ_H_ 7.42 (m, H-3´or H-3´´), δ_H_ 6.60 (d, H-2´or H-2´´), and δ_H_ 6.63 (d, H-2´´ or H-2´). The presence of an amide group in the structure was confirmed by the signal at δ_H_ 8.12 (-NH). Signals of proton resonances in the aliphatic region (δ_H_ 1.45–3.20) were also elucidated (S3 and S4 Figs in S1 File).

The presence of two cinnamamide moieties in the structure was confirmed by the HMBC experiment (Fig 4). The signals at δ_H_ 7.38 (m, H-3´´ or H-3´) and δ_H_ 7.42 (m, H-3´or H-3´´) show J^3^ couplings to the quaternary carbon signals at δ_C_ 165.27 (C-1´or C-1´´) and δ_C_ 165.32 (C-1´´ or C-1´) indicate a presence of two carbonyls in the structure (S9 and S10 Figs in S1 File). Additionally, the HMBC spectra also showed correlations of the -NH group (δ_H_ 8.12) with the above mentioned carbonyl signals (δ_C_ 165.27 and δ_C_ 165.32) that confirm the presence of two cinnamamide moieties.

In the aliphatic region of the ^13^C NMR spectra (S5 and S8 Figs in S1 File), the presence of a methyl (δ_C_ 42.09) and seven methylene carbons (δ_C_ 24–55) was confirmed by DEPT-135 (S13 and S14 Figs in S1 File). The methyl group (δ_C_ 42.09) directly linked to the nitrogen in the aliphatic chain can be confirmed in the HMBC with the long-distance proton couplings between the δ_H_ 2.16 (s, NCH_3_) and the methylene carbons at δ_C_ 55.26 (C-4) and δ_C_ 57.12 (C-6).

Seven well-defined signals of methylene carbons were found in the aliphatic region of the ^13^C NMR spectrum. The corresponding ^1^H-^13^C correlations were confirmed in the HSQC spectrum (S11 and S12 Figs in S1 File), where isochrony of two hydrogen atoms was demonstrated by the correlation of the proton resonance at δ_H_ 1.47 with two different carbon signals at δ_C_ 24.53 and δ_C_ 27.48. Furthermore, the proposed structure was confirmed by the following ^1^H-^1^H correlations in the COSY spectrum (Fig 4): δ_H_ 2.32 with δ_H_ 1.45; δ_H_ 2.35 with δ_H_ 1.61; δ_H_ 3.18 with δ_H_ 1.47, and δ_H_ 3.20 with δ_H_ 1.61 (S15 and S16 Figs in S1 File).

The MS/MS spectrum of the compound (**1)** is in agreement with the above NMR data. The ion *m/z* 420 showed sequential losses of 218 Da (*m/z* 202), 14 Da (*m/z* 188), and 57 Da (*m/z* 131) (S1 Fig in S1 File), typical for the fragmentation pattern of cinnamic acid amides [46, 59]. Therefore, the compound (**1**) was identified as 5-*N*-methylmaytenine, i.e. 1,10-di-*E*-cinnamamide of 5-*N-*methylspermidine.

The compound (**2**) appeared as a light brownish amorphous solid. The molecular formula was determined by HRMS in positive mode as C_18_H_20_NO_3_. The MS/MS spectrum of the *m/z* 298 ion showed sequential losses of 17 Da (*m/z* 281) and 15 Da (*m/z* 266), and a loss of 31 Da (*m/z* 281 → 250) (S17 Fig in S1 File); which are consistent with aporphine alkaloids containing adjacent methoxyls in the ring A and the non-substituted ring D [60, 81] (Fig 4).

The ^1^H NMR spectrum is in agreement with the MS data. It exhibited signals typical for proaporphine alkaloids at δ_H_ 2.52 (dd; 6.6 and 12 Hz; H-7 or H-7´), δ_H_ 2.42 (dd; 10.5 and 12 Hz; H-7´or H-7), δ_H_ 3.02 (m; H-4), δ_H_ 3.44 (ddd; 6.3, 11, and 13 Hz; H-5 or H-5´) e δ_H_ 3.70 (ddd; 1.5, 6.3, and 13 Hz; H-5´or H-5). The following signals were observed in the range of aromatic proton resonances: δ_H_ 6.89 (s, H-3) corresponding to an *orto*-substituted ring A; δ_H_ 7.16 (dd; 3 and 10 Hz; H-8), δ_H_ 6.29 (d; 1.8 and 10 Hz; H-9), δ_H_ 6.41 (dd; 1.8 and 10 Hz; H-11), and δ_H_ 7.01 (dd; 3 and 10 Hz; H-12) characteristic of the unsubstituted ring D; and two signals δ_H_ 3.61 (s, 3H) and δ_H_ 3.82 (s, 3H) of the methoxy-substituents in the ring A (Fig 4 and S19-S21 Figs in S1 File).

The proaporphine alkaloid structure was proposed based on the HMBC experiments (Fig 4). The signals at δ_H_ 7.16 (dd; 3 and 10 Hz; H-8) and δ_H_ 7.01 (dd; 3 and 10 Hz; H-12) showed a J^3^-coupling to the carbon at δ_C_ 186.6 (C-10). The proaporphine skeleton was also established following long-distance ^1^H-^13^C couplings of the signals at δ_H_ 6.89 (s, H-3) and those of the methoxyls at δ_H_ 3.61 (s, 3H) and δ_H_ 3.82 (s, 3H) with the carbons at δ_C_ 144.6 (C-1) and δ_C_ 154.7 (C-2), thus confirming the existence of two substitutions in the A ring (S24 and S25 Figs in S1 File). Therefore, the compound (**2**) was elucidated as being the proaporphine alkaloid stepharine.

The compounds were identified by a comparison of the obtained spectral results with data reported in the literature [46, 59, 82]. The alkaloid (**1**) was identified as 5-*N*-methylmaytenine, this is the first report on isolation of this compound from a natural product, as well as the first occurrence of the alkaloid in the Menispermaceae family [59].

The proaporphine alkaloid stepharine (**2**) was also described by us for the first time in *A*. *panurensis*. Previously, its presence was reported in *Stephania* genus [83–89], as well as in some *Abuta* species [4, 15].

### Acetylcholinesterase inhibition

The docking study provided a very useful tool in the interpretation of the AChE inhibitory activity results, indicating that molecules under investigation were able to bind effectively to the TcAChE enzyme active site.

**Fig 5:**
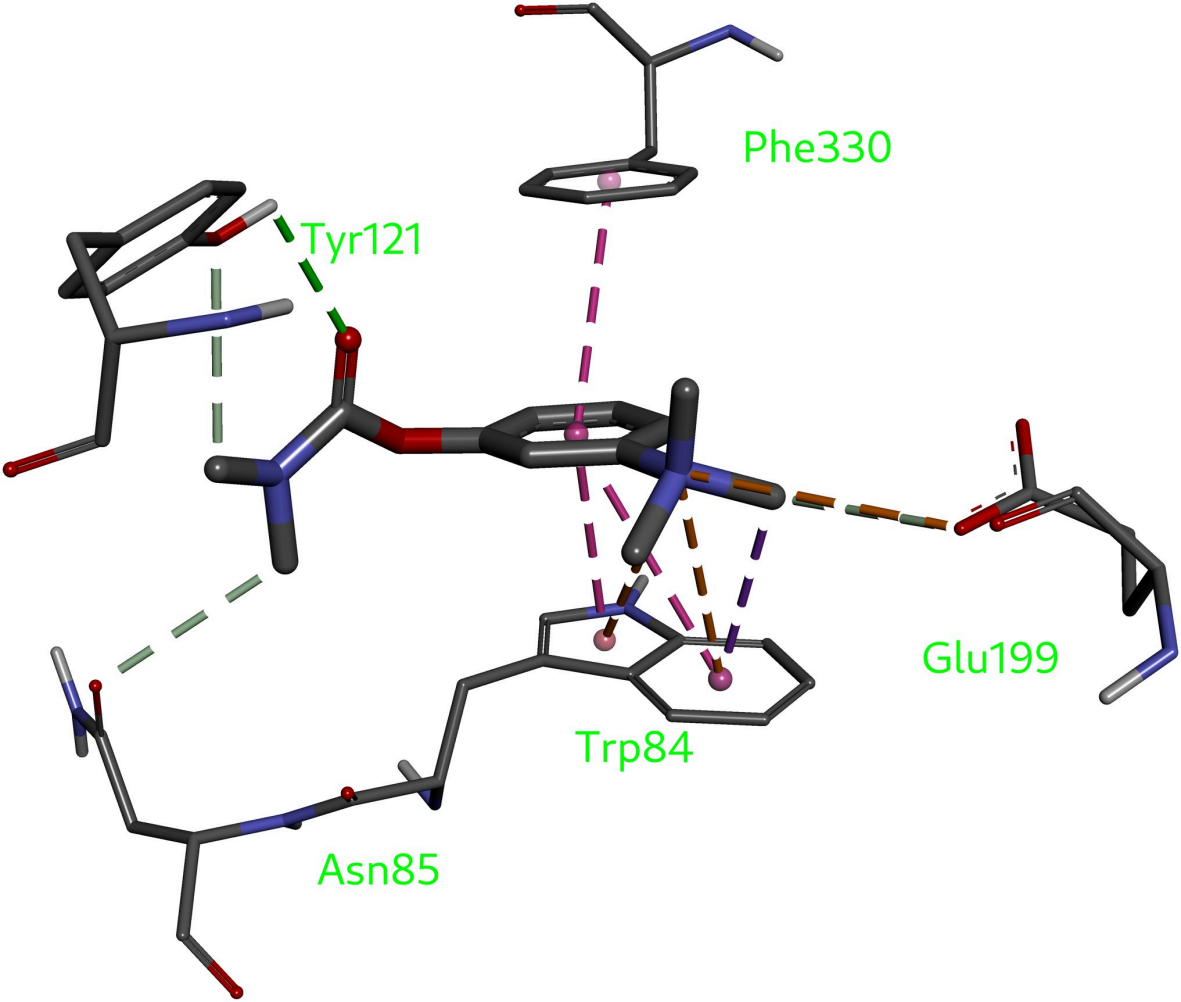
Neostigmine interactions with the AChE binding pocket.

The neostigmine (used as a positive control in the assay) forms π-stacking interactions with the aromatic rings of amino acids from the so-called anionic subsite of the enzyme corresponding to the choline-binding pocket (Trp84 and Phe330). The stronger one is formed with the phenyl ring of Trp84 (3.66 Å). The stacking distance to Phe330 is 4.31 Å implying a weaker interaction. There exist two attractive Coulomb interactions as well. The first one is between the positively charged quaternary ammonium nitrogen and a carboxylic group of the Glu199. The second interaction is formed between the same charged nitrogen atom and a phenyl ring of the Trp84, at a distance of approximately 4.4 Å, typical for tetraalkylammonia–π interactions [90] (Fig 5).

**Fig 6:**
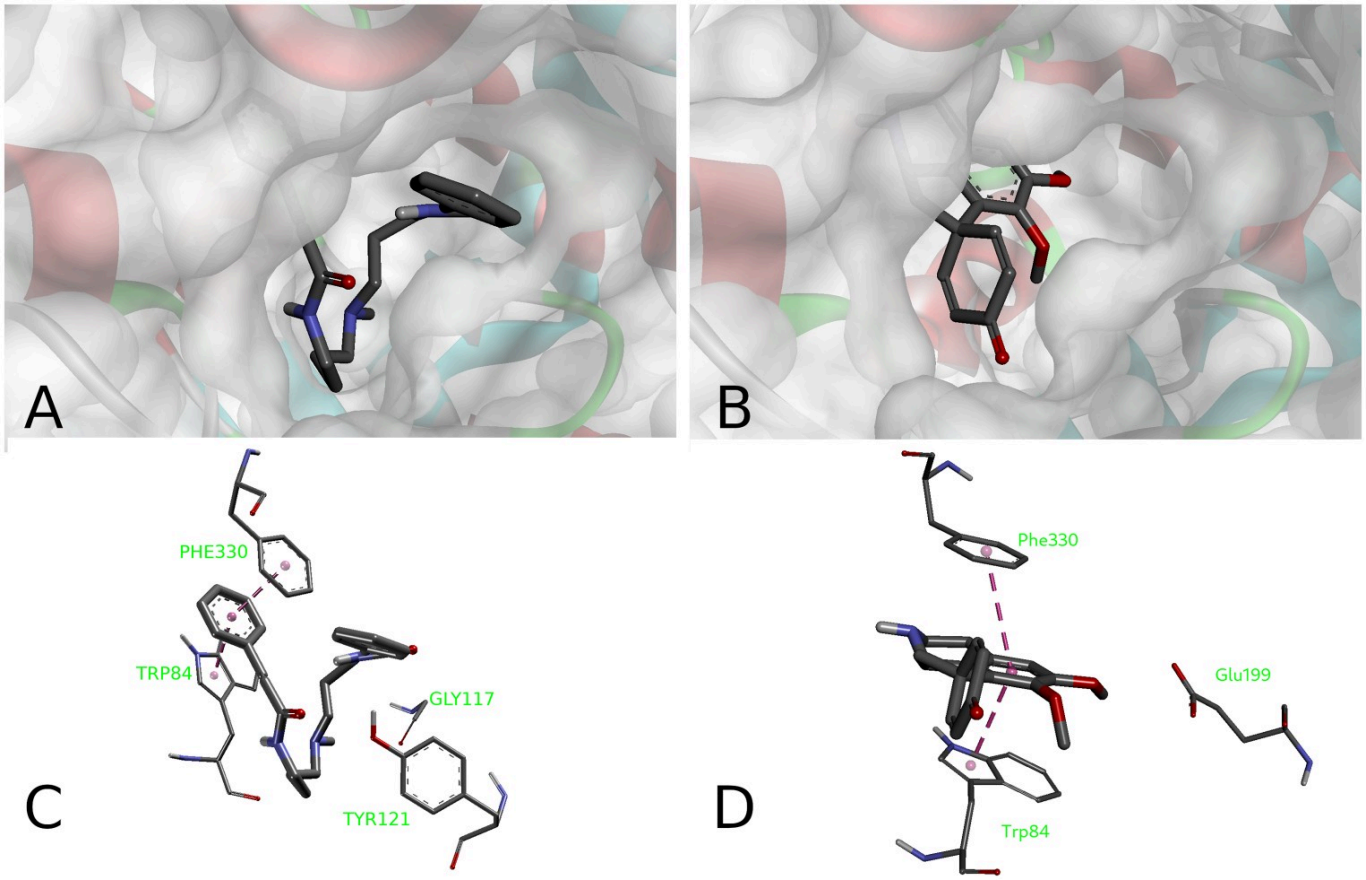
Binding poses (A, B) of the alkaloids inside the AChE pocket and their binding modes (C, D). 5-*N*-methylmaytenine (A, C) and stepharine (B, D).

Because the active site pocket of the acetylcholinesterase forms a deep and narrow gorge, formed of aromatic residues by 40% [91], the hydrophobic interactions play a major role in binding of the compounds under investigation to the AChE (Figs 5 and 6). Docking data indicate that (**1**) is a plausible ligand for the AChE, which can adopt a variety of binding poses due to its conformational flexibility. One of the cinnamic acid phenyls is sandwiched between two aromatic residues of the enzyme Trp84 and Phe330 at the distance of 3.6Å…3.9Å that is slightly above a typical value for a parallel displaced π-stacking. Interactions with peripheral subsites Tyr121 and Gly117 were also observed. Stepharine (**2**) fits well inside the AChE pocket with the predicted binding affinity of -10.3 kcal/mol. This energy corresponds to substantial interaction between the ligand and the enzyme. (Fig 6). π-Stacking is again the major protein-ligand interaction for this compound (3.66 Å with Trp84 pyrrole and 4.09 Å with Phe330 phenyl rings). Both alkaloids have their binding poses at the lipophilic anionic subsite (Trp84 and Phe330) of the TcAChE (Fig 6), where they are able to interact with the enzyme pocket [91, 92].

Overall, all three compounds studied (**1**, **2**, and neostigmine) have phenyl rings as the most important structural features that lead to similar interactions guiding their affinity towards the AChE. The preferential interaction of the neostigmine quaternary nitrogen with the π electrons of the aromatic rings at the binding site of the enzyme indicates greater affinity of that subsite towards easily polarizable ammonium ligands. [93]. The alkaloids in question have no such moieties, which can be a reason for higher IC_50_ values of the **1** and **2**, than that of neostigmine.

Peripheral anionic binding site of the enzyme is also associated with the AChE-mediated abnormal β-amyloid protein aggregation in Alzheimer’s disease patients [94, 95]. It is interesting to note that owing to a conformational flexibility of the molecule, 5-*N-*methylmaytenine interacts simultaneously with both the anionic and the peripheral subsites of the enzyme. Therefore, **(1)** may well be capable to have greater pharmacological potential [94], when compared to stepharine that interacts with the anionic active subsite only.

Currently, natural products constitute one of the main sources of the AChE inhibitors, used as active compounds to treat damages to central and peripheral nervous system, as well as to alleviate symptoms of neurodegenerative diseases [28, 96, 97]. Bisbenzylisoquinoline and protoberberine alkaloids exhibit a moderate AChE enzyme inhibition potential with IC_50_ values in the range of 34.66μM to 78.22μM [17] and 36.6μM to 141.8μM [98], respectively. Whereas aporphine and proaporphine alkaloids demonstrate better AChE inhibitory activity with the IC_50_ values ranging from 2.98μM to 20.4μM and this effect is often related to different substituents in their structure [27]. Polyamine alkaloids such as putrescine, spermidine, spermine, cadaverine, and their derivatives are present ubiquitously in all living cells; they have a variety of functions inside the cell, including the cell growth and regeneration [99]. For the central nervous system in particular, studies show that polyamines act on receptors related to neurodegenerative processes [100, 101]. For example, the spermidine decreases significantly the AChE activity, oxidative stress and neuroinflammation in a cerebral hippocampus [102].

Our study of two alkaloids first obtained from *A*. *panurensis* revealed that both compounds are promising AChE enzyme inhibitors. The capability of 5-*N*-methylmaytenine to interact simultaneously with several subsites in the enzyme structure may provide an important guideline in the search for new active compounds for treatment of neurological disorders [103].

### Cytotoxicity assay

**Fig 7:**
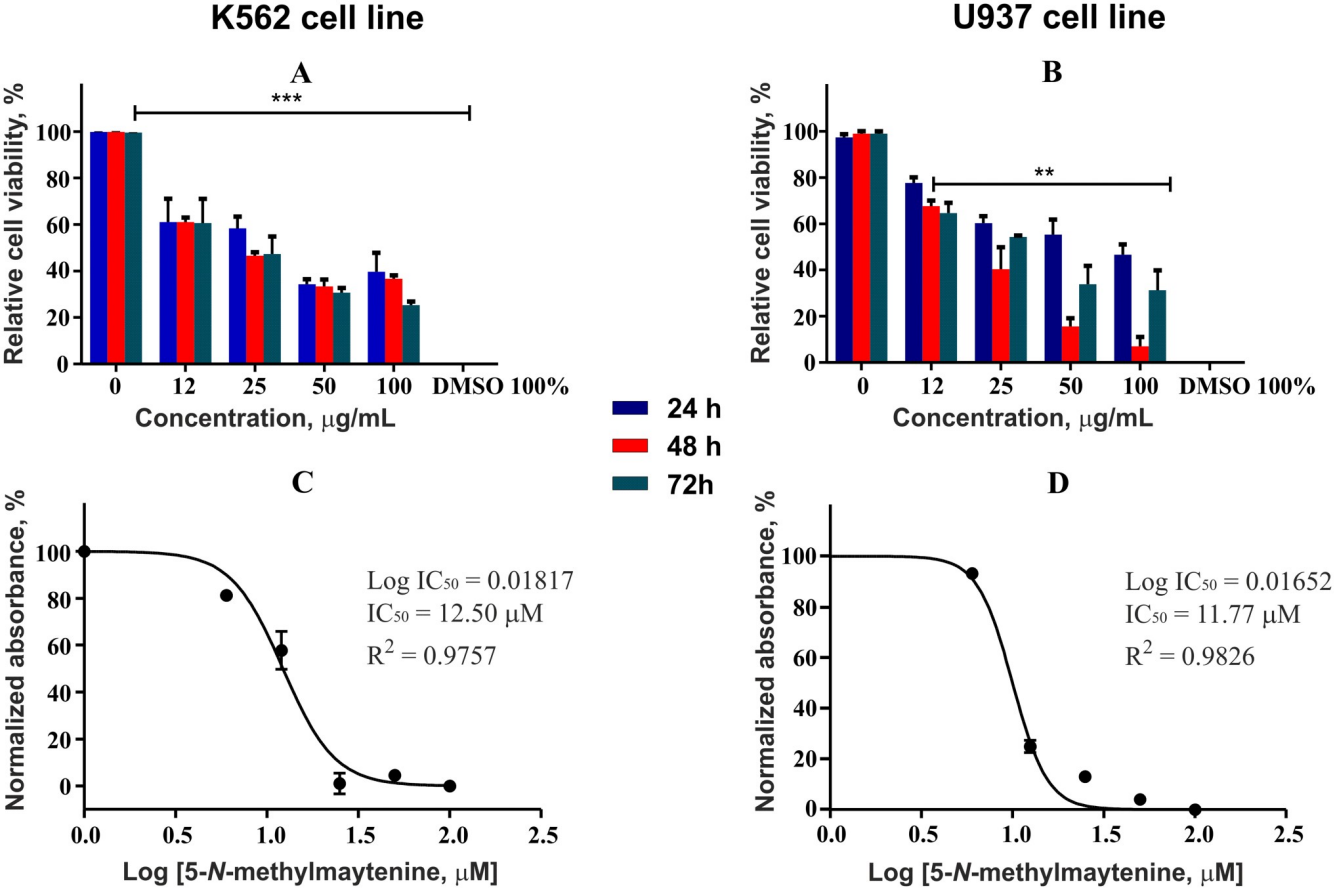
Cytotoxic activity of 5-*N*-methylmaytenine respective IC_50_ values.

**Fig 8:**
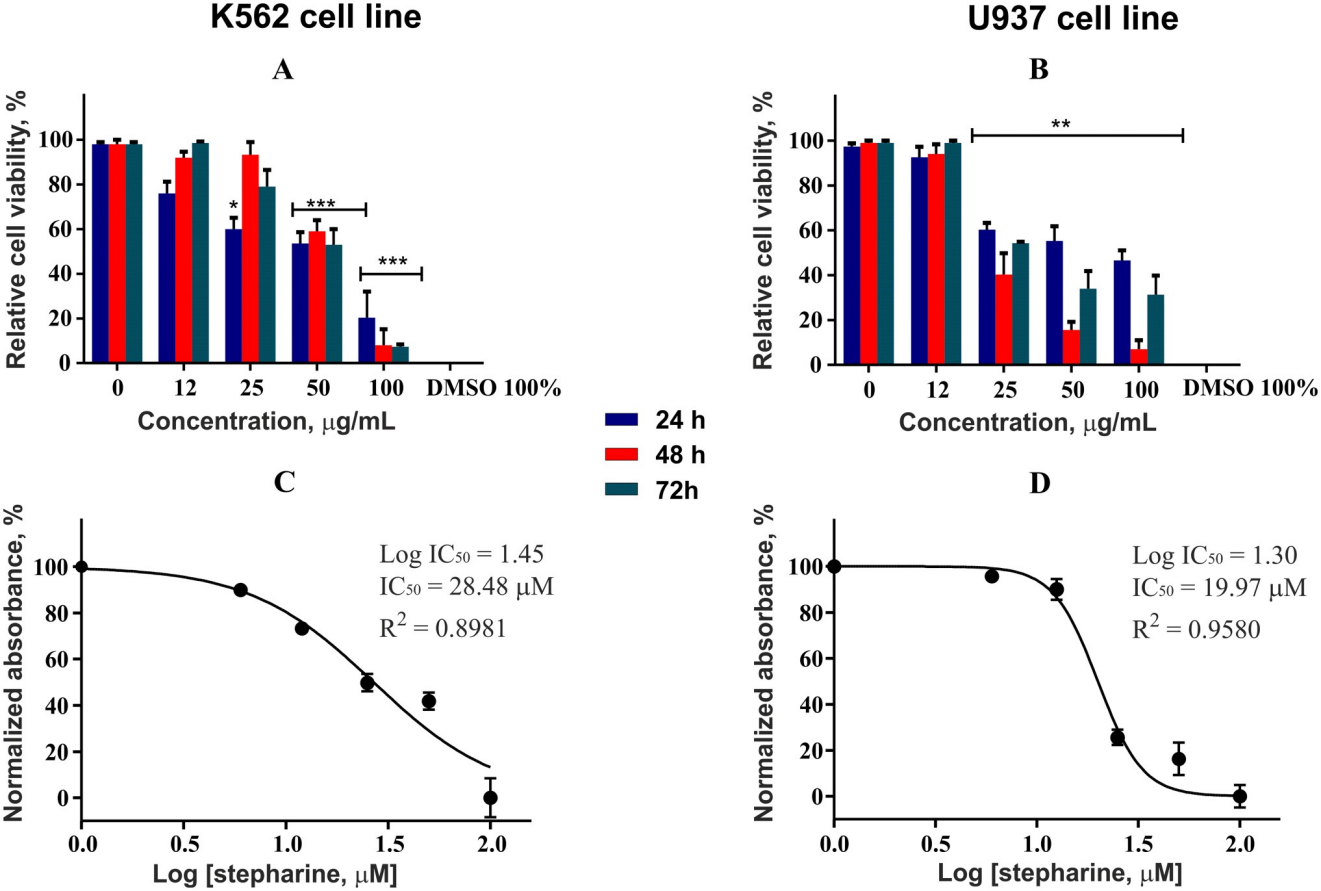
Cytotoxic activity of stepharine and respective IC_50_ values.

**Fig 9:**
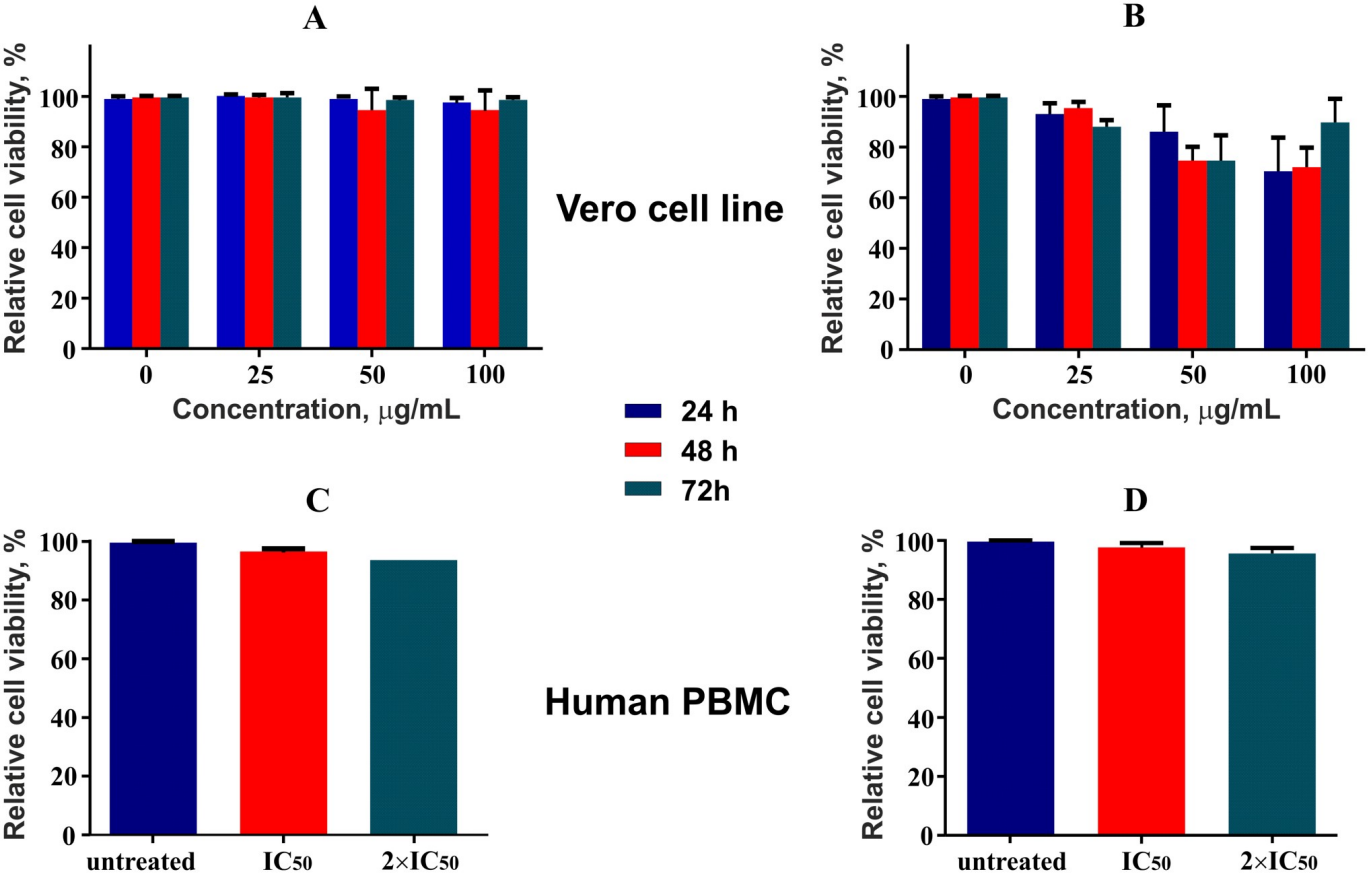
Cytotoxicity assessment of the alkaloids to Vero and human PBMC cells. Stepharine (A, C) and 5-*N*-methylmaytenine (B, D).

Effectiveness of 5-*N*-methylmaytenine (Fig 7) and stepharine (Fig 8) in different concentrations were evaluated against U937 and K562 tumor cell lines. The U937 cells were more susceptible to the stepharine anticancer action. Interestingly, the stepharine demonstrated higher cytotoxic activity against the K562 strain only in the first 24 hours of treatment. This result suggest that K562 cell line is more resistant to the stepharine treatment than U937. Both compounds showed practically no toxicity to non-cancerous cells (Vero and human PBMC, Fig 9).

Although stepharine showed greater stability of action in the course of the assay, the 5-*N-*methylmaytenine demonstrated higher efficiency, according to the IC_50_ values.

The results obtained corroborate well with the literature reports that isoquinoline alkaloids of different backbones, such as berberine and aporphine ones containing an oxo-substituent in their structure, particularly oxoisoaporphines, demonstrate moderate to strong potential for cytotoxic activity against several tumor cell lines, including those used in the present work [96, 104–107].

### Immunomodulatory assay

The anti-inflammatory properties of acetylcholinesterase inhibitors are mediated by a cholinergic system present in the immune cells [108, 109]. For instance, macrophages and T cells express α7 homopentameric nAChR receptor that can down-regulate the production of inflammatory cytokines (TNF-α and IL-1β) and NF-κB-dependent transcription, when stimulated [110]. The latter pathway regulation may be involved in the pathogenesis of many chronic diseases such as asthma, rheumatoid arthritis, atherosclerosis, and even though the Alzheimer’s disease [111–113]. In this study, we did not assess the potential of stepharine and 5-*N*-methylmaytenine in regulating the NF-κB immune pathway. However, our findings demonstrate that the cholinergic anti-inflammatory pathway could reduce the production of IL-6 and IL-8 owing to the AChE inhibitor treatment, which can protect against the damage provoked by inflammation in different types of inflammatory diseases.

According to the molecular docking studies, both alkaloids bind to the interleukins with quite similar affinity in the range of -8 …-6 kcal/mol.

#### Binding to the IL-6

**Fig 10:**
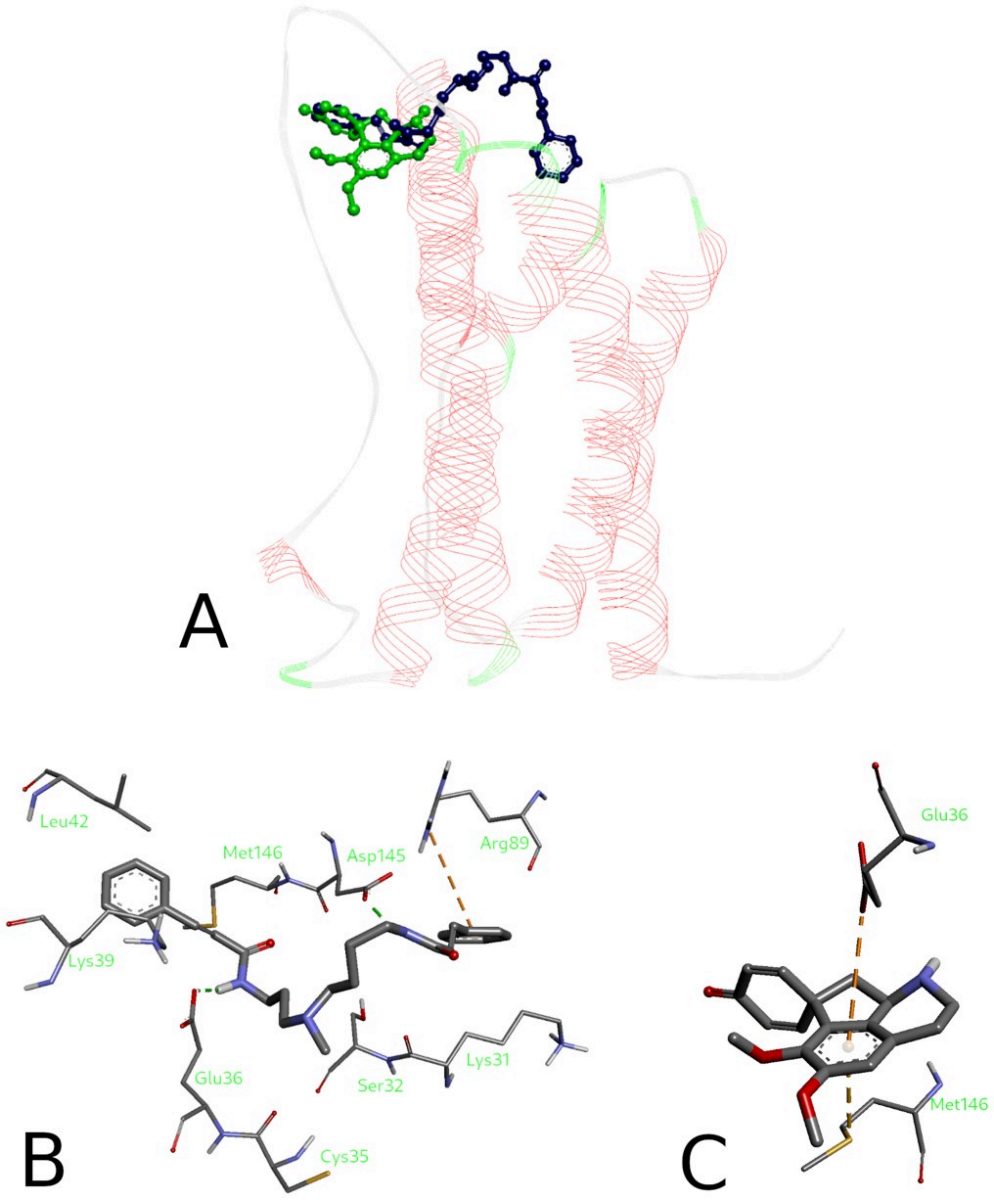
Binding poses (A) of the alkaloids at the IL-6 and their binding modes (B, C). 5-*N*-methylmaytenine (blue A, B) and stepharine (green A, C).

In the case of IL-6, 5-*N*-methylmaytenine binds to the upper rim of the protein (Fig 10), giving rise to hydrogen bonds with carboxylic groups of Glu36 (modeled loop, 2.20 Å) and Asp145 (1.94 Å). A weak cation-π interaction with Arg89 guanidine moiety and favorable hydrophobic interactions between the phenyl ring of **1** and carbon chains of Leu42 and Lys39 were also formed.

Similarly, stepharine (**2**) binds to the top region of the IL-6 interacting with Met146 of the α-helix (π-sulfur interaction at 3.65 Å) and with Glu36 of the modeled loop (weak anion-π interaction with the carboxyl group).

**Fig 11:**
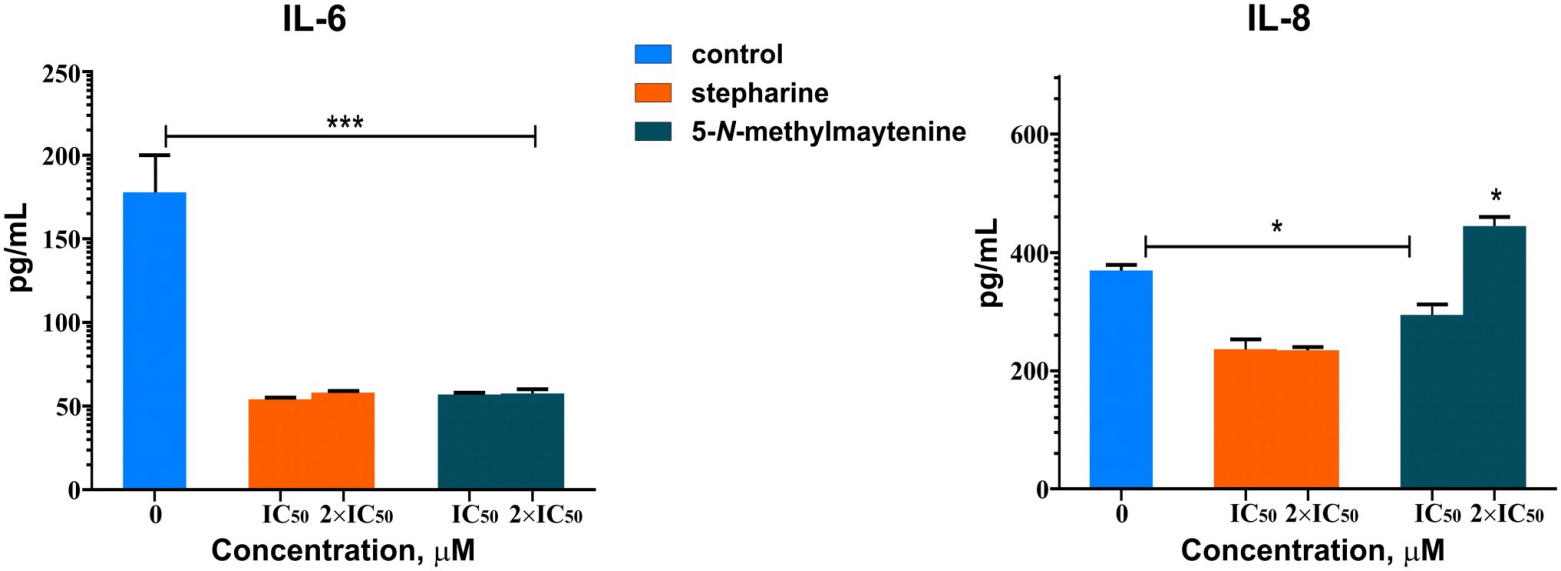
Immunomodulatory effect of 5-*N*-methylmaytenine and stepharine.

Both alkaloids interact with the Site 3 of the IL-6 (Fig 2) indicating that they are able to disrupt IL-6 –gp130 complex formation and thus hinder the signaling. Results of the immunological assay are in agreement with the above statement (Fig 11).

#### Binding to IL-8

**Fig 12:**
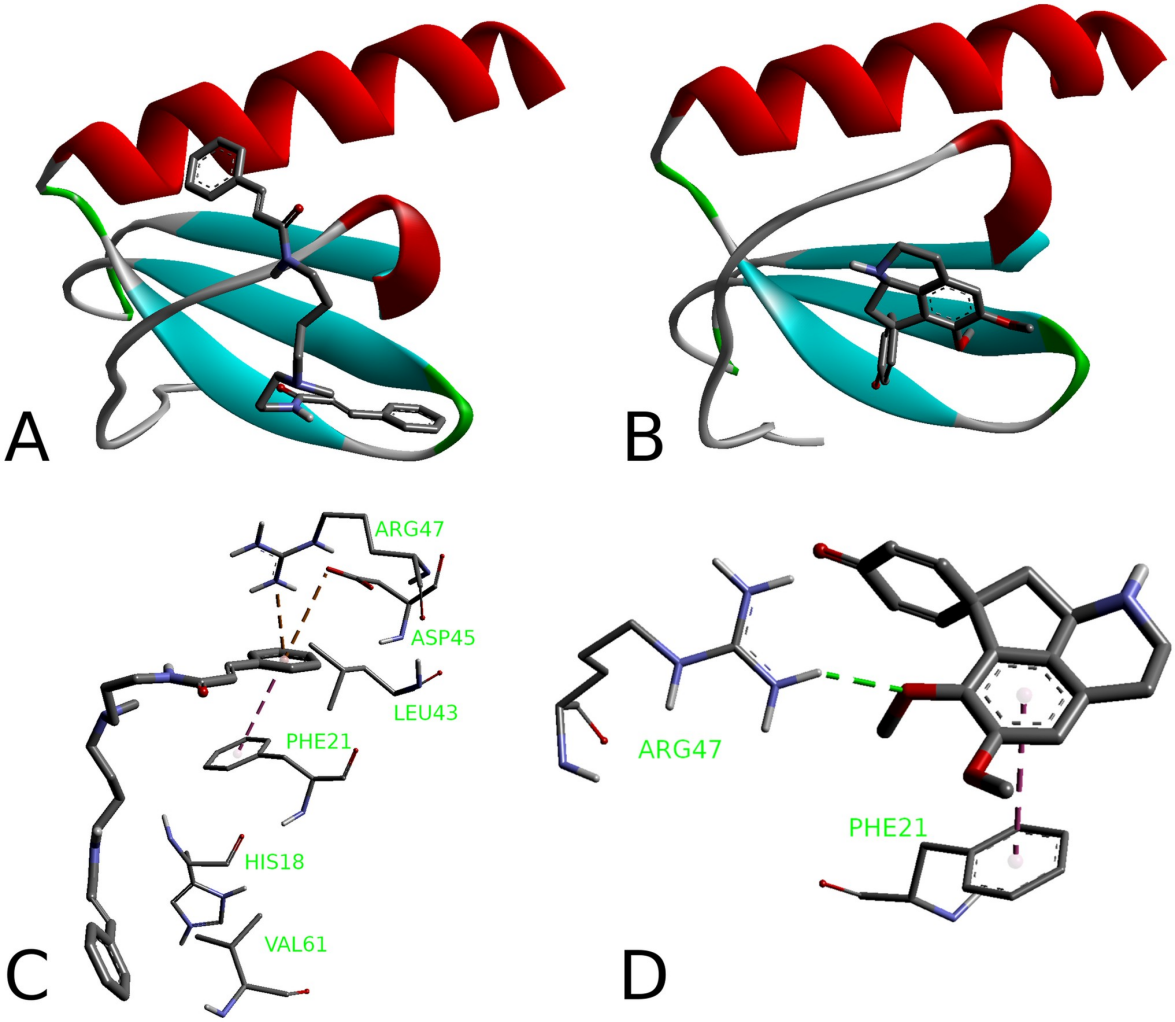
Binding poses (A, B) of the alkaloids at the IL-8 and their binding modes (C, D). 5-*N*-methylmaytenine (A, C) and stepharine (B, D).

According to the docking results, both alkaloids bind into a shallow pocket between a Thr12 –Pro19 loop and a β-sheet region Ile40 –Cys50 (Fig 12), which are close to the receptor-binding Site I (Fig 3); implying inhibitory action of both **1** and **2**. 5-*N*-methylmaytenine forms an aromatic interaction with the Phe21 phenyl ring and two π-charge interactions with the Arg47 guanidine moiety of and the Asp45 carboxyl group. Stepharine binds to the protein pocket in a similar way by π-π stacking interaction with Phe21, but acts as a hydrogen-bond acceptor for the -NH proton of Arg47 (Fig 12). This fact may suggest a slightly stronger inhibition of IL-8 production by stepharine. In the case of 5-*N*-methylmaytenine, the induction of the IL-8 level downregulation seems to happen in a dose-dependent manner (Fig 11).

**Fig 13:**
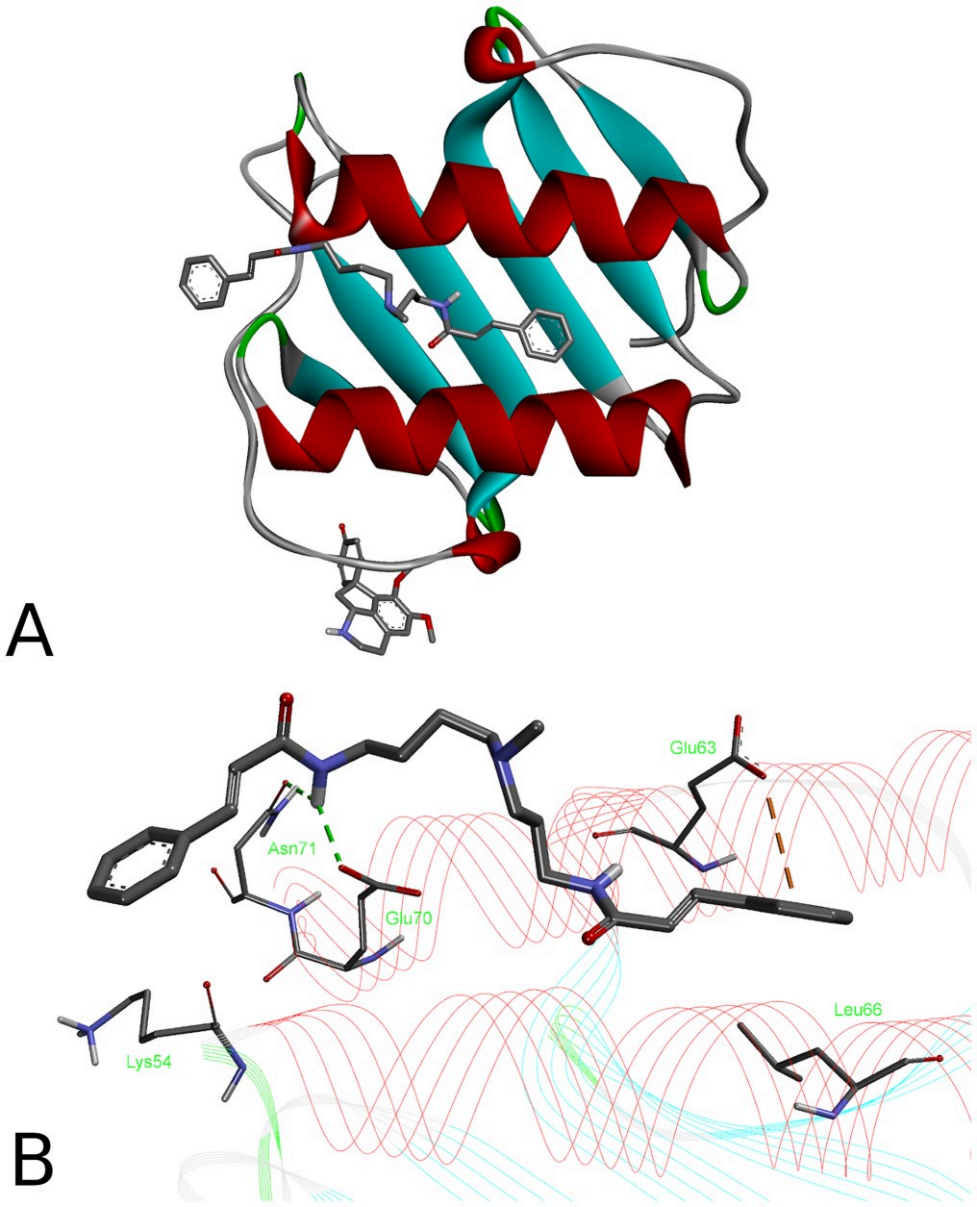
Binding poses of the alkaloids at the IL-8 dimer (A) and binding mode of 5-*N*-methylmaytenine (B). Stepharine-dimer binding mode is similar to the one shown in the Fig 12D.

It was shown that some interleukins, including the IL-8, can exist both as dimers and monomers in solution [78]. The dimerization occurs at higher IL-8 concentrations and is highly sensitive to solution conditions such as pH and ionic strength [114, 115]. In general, affinity to the receptors and activity of the dimers are much lower than those of the monomers [114, 116]. We conducted docking studies of 5-*N*-methylmaytenine and stepharine to the IL-8 dimer (Fig 13).

Affinities of the **1** and **2** to the dimer are similar to those of the monomeric protein: -7.0 kcal/mol for **1** and -5.9 kcal/mol for **2**. Inclusion of water molecules into the dimer structure did not lead to improvement of binding affinities. It is interesting to note that binding positions of the two ligands became different. While stepharine binds to the dimer the same way as it interacts with the monomer (Fig 12), the 5-*N*-methylmaytenine molecule is stretched between α-helices and interacts with both monomer moieties. Most of the direct interactions were found with one of the IL-8 units, *viz*., two hydrogen bonds with oxygen atoms of Asn71 and Glu70 (2.71 Å and 2.38 Å, respectively) and a weak anion-π interaction between the phenyl ring of **1** and a carboxylic group of Glu63 (3.85 Å). Carbon chains of Lys54 and Leu66 of another IL-8 molecule form hydrophobic interactions with the phenyl rings of 5-*N*-methylmaytenine. At lower concentrations, **1** is able to interact with the IL-8 dimers and possibly to prevent their dissociation into more active monomer form. At higher concentrations, the binding of 5-N-methylmaytenine to the IL-8 monomers and their activation may take place.

Mostly, IL-6 and IL-8 play a crucial role in the selective chemotaxis, degranulation, and activation of neutrophils. High levels of those interleukins are associated with an immunopathogenesis of many chronic inflammation diseases, such as cell injury in kidney inflammation, poor outcome of different neurological manifestations, and excessive infiltration of neutrophils in airways of cystic fibrosis patients [117–119]. Thus, therapies targeting pro-inflammatory cytokines, such as IL-6 and IL-8, have important clinical implications.

## Conclusion

This is the first paper dealing with 5-*N*-methylmaytenine isolation from natural products and the first report on the occurrence of both 5-*N*-methylmaytenine and stepharine alkaloids in branches of the Amazonian plant *Abuta panurensis*. Inhibitory activity of both alkaloids towards the AChE enzyme was evaluated by spectrophotometry and molecular docking study. The compounds in question bind effectively to the enzyme active site and demonstrate promising inhibitory potential. Owing to the greater conformational flexibility of 5-*N*-methylmaytenine, it is capable to interact with both anionic and peripheral subsites, thus demonstrating higher AChE inhibition potential, when compared to stepharine.

Both alkaloids were effective against K562 and U937 tumor cells, showing practically no toxicity to normal cell lines Vero and human PBMC.

5-*N*-methylmaytenine and stepharine demonstrated immunomodulatory activity towards IL-6 and IL-8 interleukins. In the former case, both alkaloids inhibited the IL-6 production at very similar levels, which may probably be related to the formation of hydrogen bonds with the protein binding sites. In the latter case, stepharine inhibited considerably the production of IL-8 in comparison to 5-*N*-methylmaytenine that showed a dose dependent action (inhibitory at the IC_50_ dose, and stimulatory at the twofold IC_50_ one). Such a behavior may possibly be explained by different binding modes of the alkaloids to the interleukin monomer and dimer forms.

Our results suggest that 5-*N*-methylmaytenine and stepharine could be used as reversible AChE inhibitors in the treatment of neurological disorder manifestations, as well as candidate immunomodulatory agents in the inflammatory disease context. However, more research is necessary to better investigate the complete pharmacological potential and toxicological profile of these compounds.

## Supporting information

S1 File (PDF)

## Abbreviations

1D NMR: One-dimensional NMR
2D NMR: Two-dimensional NMR
AChE: acetylcholinesterase
AChI: acetylcholine iodide
C18: Octadecyl Carbon Chain
COSY: Correlated Spectroscopy
DAD: Diode Array Detection
DEPT-135: Distortionless Enhancement by Polarization Transfer
DMSO: dimethylsufoxide
DTNB: 5,5´-dithio-bis(2- nitrobenzoic)acid
ELISA: Enzyme-Linked Immunosorbent Assay
FBS: Fetal bovine serum
HMBC: Heteronuclear Multiple Bond Correlation
HPLC: High Performance Liquid Chromatography
HRMS: High Resolution Mass Spectrometry
HSQC: Heteronuclear Single Bond Correlation
IC_50_: Half Minimal Inhibitory Concentration
IL: Interleukin
LC-APCI-MS: Liquid Chromatography—Atmospheric Pressure Chemical Ionization—Mass Spectrometry
*m/z*: Mass-to-charge ratio
MS/MS: Tandem Mass Spectrometry
MTT: 3-(4,5-dimethylthiazol-2-yl)-2,5-diphenyltetrazolium bromide
NMR: Nuclear magnetic resonance
PBMC: peripheral blood mononuclear cells
PBS: Phosphate buffer saline
PFP: Pentafluorophenyl
RMSD: Root-mean-square deviation
TMS: Tetramethylsilane
UCSF: University of California, San Francisco
UV: Ultraviolet
XRD: X-ray diffraction
μM: micromolar
