# Supplementary material for: Alkaloids of Abuta panurensis Eichler: In silico and in vitro study of acetylcholinesterase inhibition, cytotoxic and immunomodulatory activities
Source: PLoS One. 2020 Sep 29;15(9):e0239364. doi: 10.1371/journal.pone.0239364 (PMC7523975; doi:10.1371/journal.pone.0239364)
Supplement: S1 File — (PDF) [file pone.0239364.s001.pdf]

Supporting material for the manuscript

Alkaloids of *Abuta panurensis* Eichler: *in silico* and *in vitro* study of acetylcholinesterase inhibition, antitumor and immunomodulatory activities

by

Rochelly da Silva Mesquita, Andrii Kyrylchuk, Regiane Costa de Oliveira, Ingrity Suelen Costa Sá, Gabriel Coutinho Borges Camargo, Gemilson Soares Pontes, Felipe Moura Araújo da Silva, Rita de Cássia Saraiva Nunomura, and Andriy Grafov

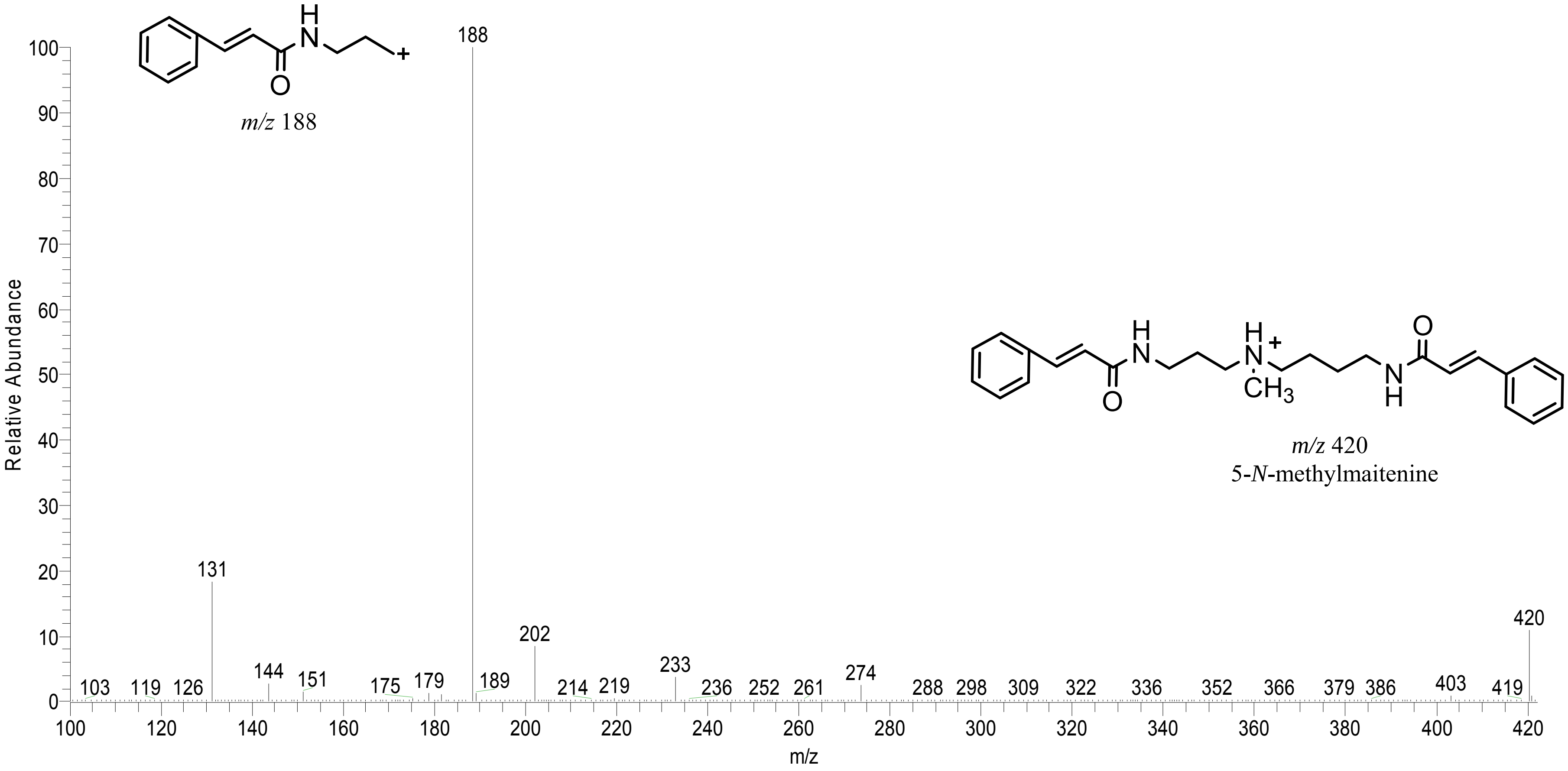

S1 Fig. MS/MS mass spectrum of 5-*N*-methylmaytenine with chemical structure and identification of the main fragment ions.

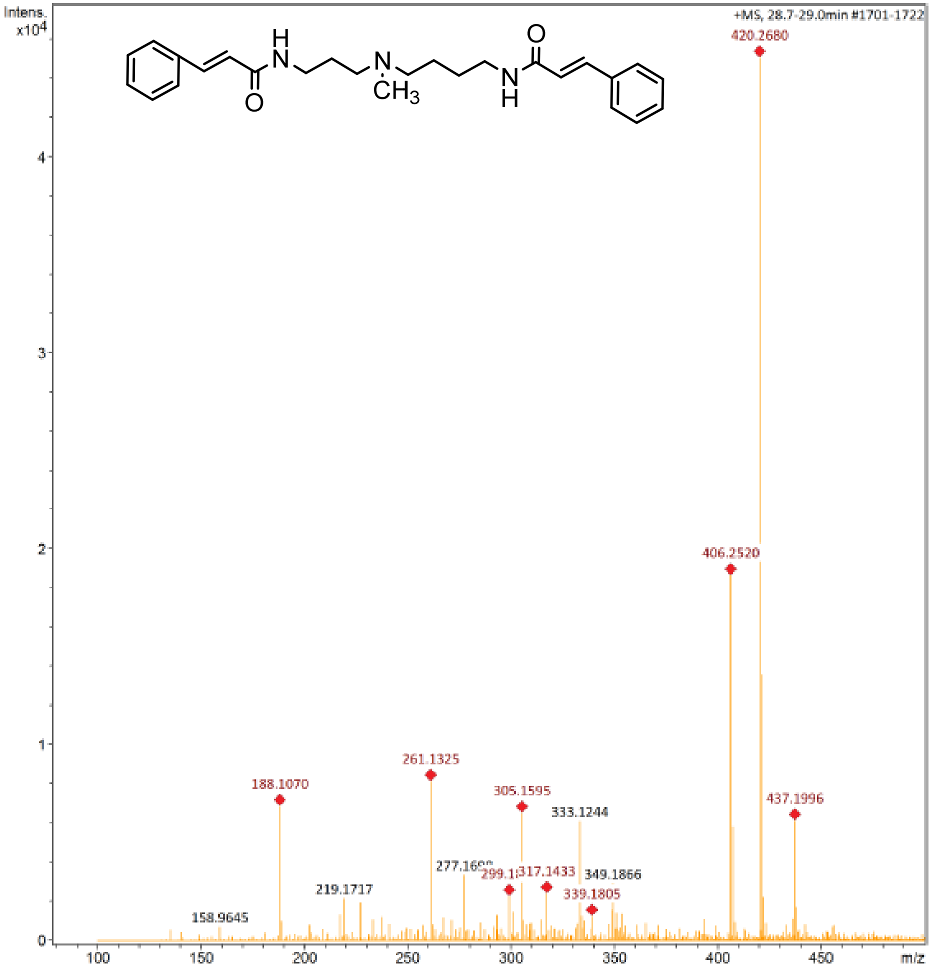

S2 Fig. Hight resolution mass-spectrum of 5-*N*-methylmaytenine with chemical structure.

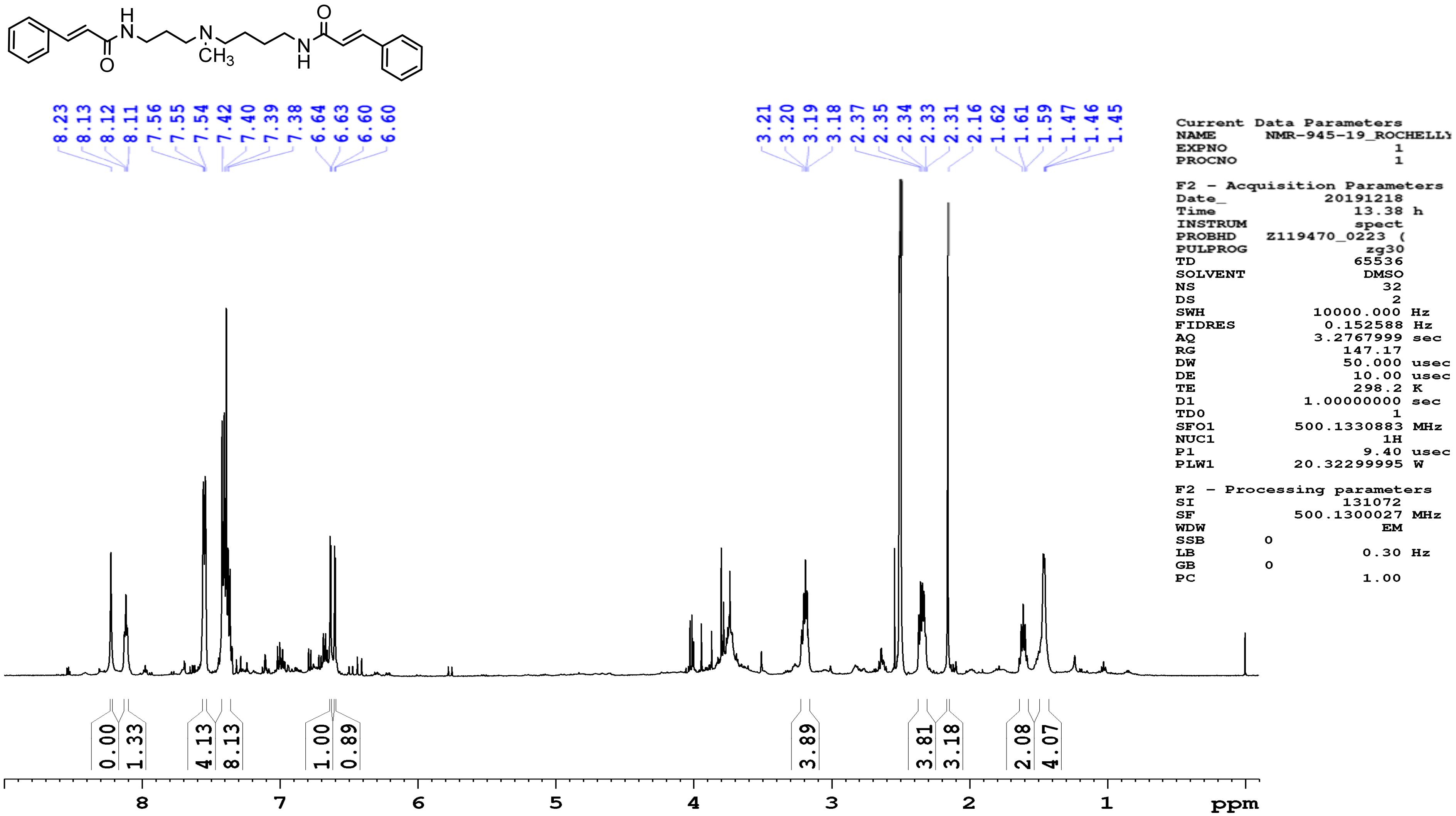

S3 Fig. ^{1}H NMR spectrum (500 MHz, DMSO d_6, TMS) of 5-*N*-methylmaytenine.

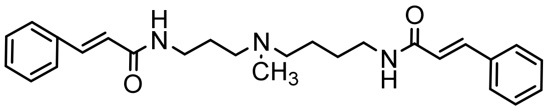

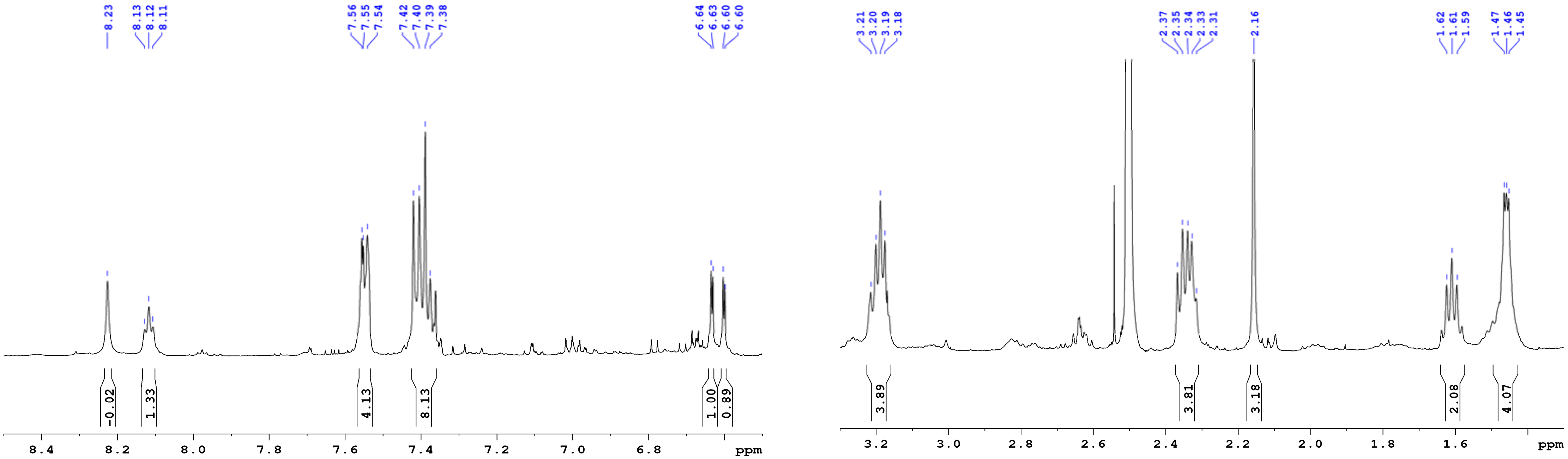

S4 Fig. Expanded low-field region of the ^{1}H NMR spectrum (500 MHz, DMSO d_6, TMS) of 5-*N*-methylmaytenine.

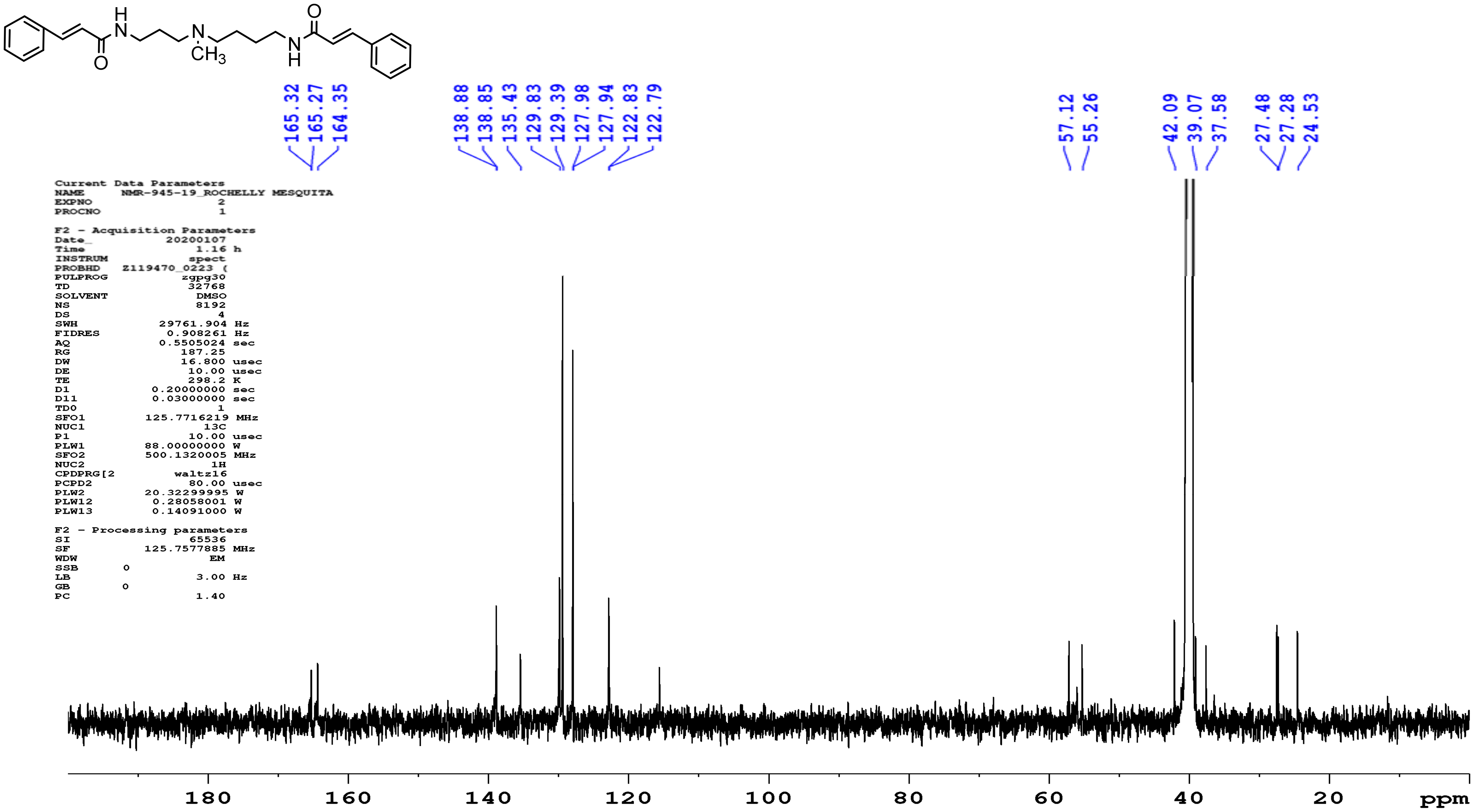

S5 Fig. ^{13}C NMR spectrum (125 MHz, DMSO d_6, TMS) of 5-*N*-methylmaytenine.

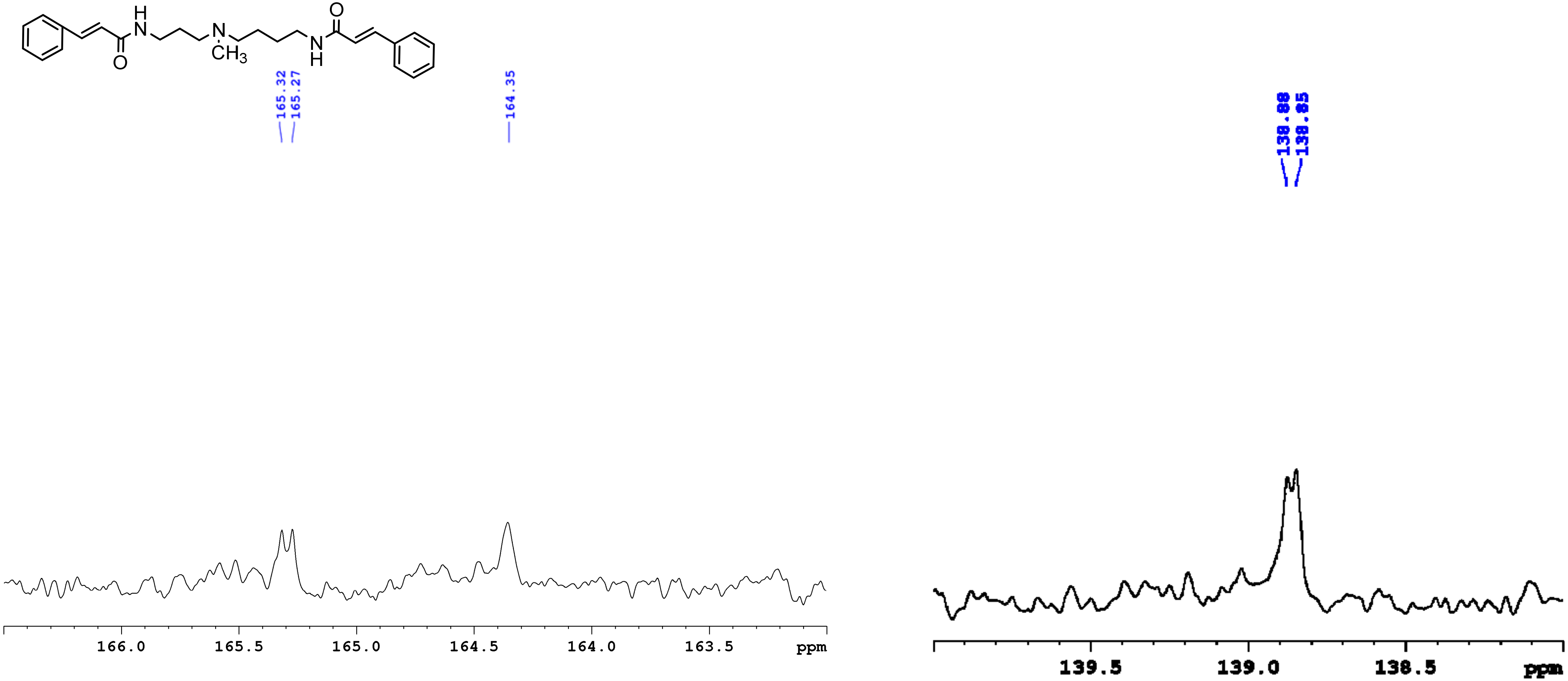

S6 Fig. Expanded regions of the ^{13}C NMR spectrum (125 MHz, DMSO d_6, TMS) of 5-*N*-methylmaytenine.

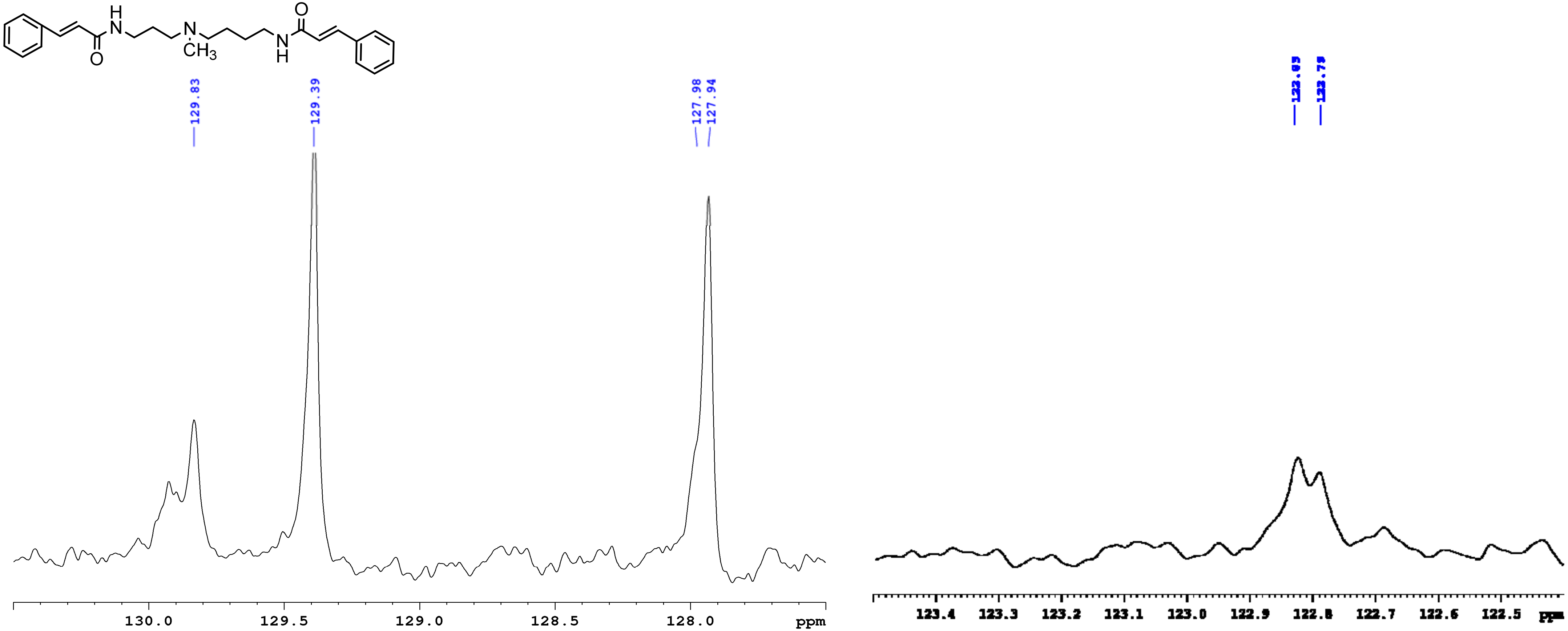

S7 Fig. Expanded regions of the ^{13}C NMR spectrum (125 MHz, DMSO d$_6$, TMS) of 5-*N*-methylmaytenine.

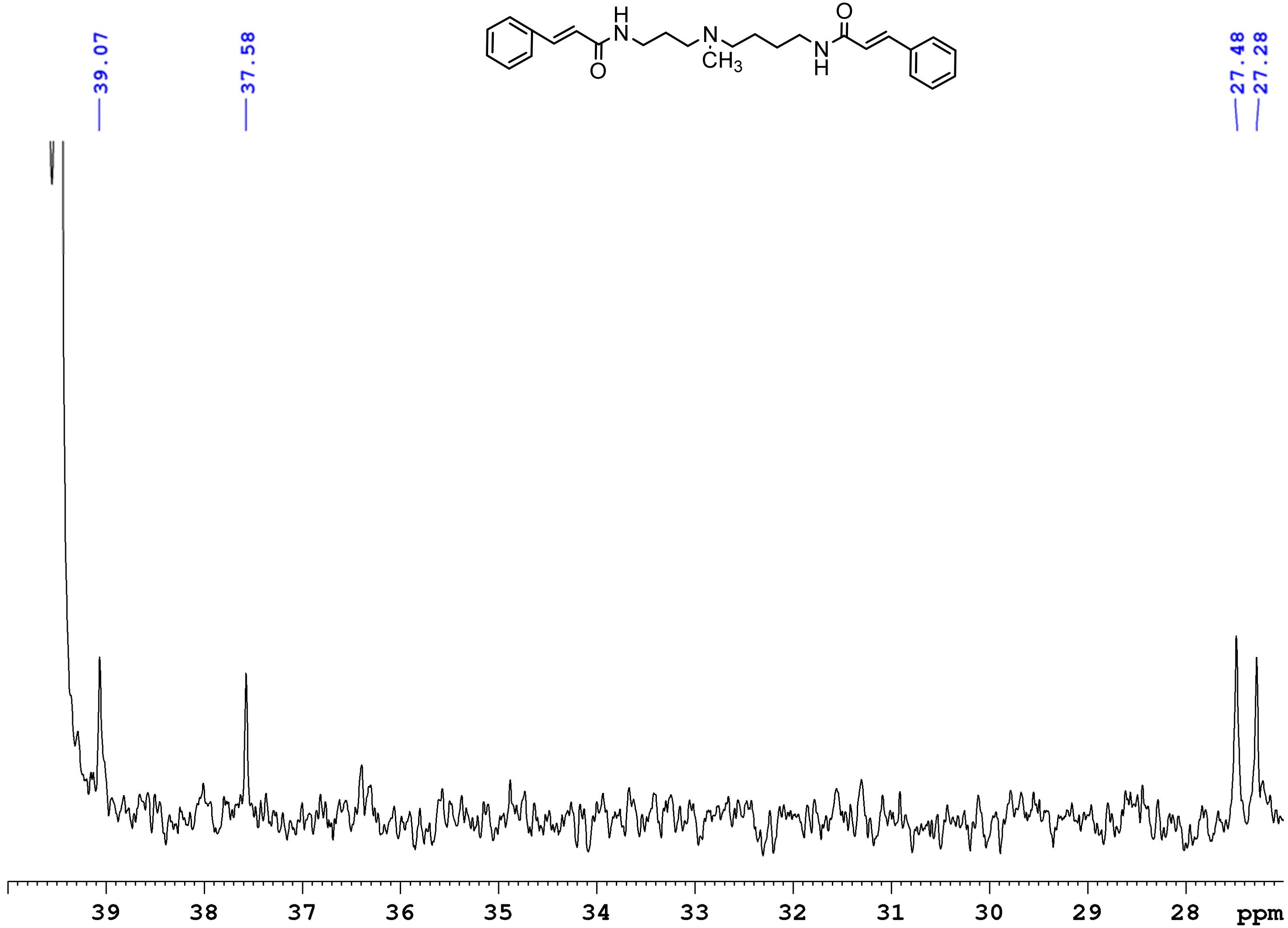

S8 Fig. Expanded region of the ^{13}C NMR spectrum (125 MHz, DMSO d_6, TMS) of 5-*N*-methylmaytenine.

```
Current Data Parameters
NAME      NMR-945-19_ROCHELLY MESQUIT
EXPNO                 300
PROCNO                  1

F2 - Acquisition Parameters
Date_            20200107
Time                 5.23 h
INSTRUM             spect
PROBHD    Z119470_0223 (
PULPROG      hmbcgplpndqf
TD                   2048
SOLVENT              DMSO
NS                     52
DS                     16
SWH              5980.861 Hz
FIDRES           2.920342 Hz
AQ              0.1712128 sec
RG                 187.25
DW                 83.600 usec
DE                  10.00 usec
TE                  298.1 K
CNST2         145.0000000
CNST13          8.0000000
D0             0.00000300 sec
D1             1.00000000 sec
D2             0.00344828 sec
D6             0.06250000 sec
D16            0.00020000 sec
IN0            0.00001660 sec
TDav                    1
SFO1          500.1325072 MHz
NUC1                   1H
P1                   9.40 usec
P2                  18.80 usec
PLW1          20.32299995 W
SFO2          125.7716219 MHz
NUC2                  13C
P3                  10.00 usec
PLW2          88.00000000 W
GPNAM[1]      SMSQ10.100
GPZ1                50.00 %
GPNAM[2]      SMSQ10.100
GPZ2                30.00 %
GPNAM[3]      SMSQ10.100
GPZ3                40.10 %
P16               1000.00 usec

F1 - Acquisition parameters
TD                    235
SFO1             125.7716 MHz
FIDRES         256.344513 Hz
SW                239.486 ppm
FnMODE                 QF

F2 - Processing parameters
SI                   4096
SF            500.1300000 MHz
WDW                  SINE
SSB       0
LB        0 Hz
GB        0
PC                   1.40

F1 - Processing parameters
SI                   1024
MC2                    QF
SF            125.7577885 MHz
WDW                  SINE
SSB       0
LB        0 Hz
GB        0
```

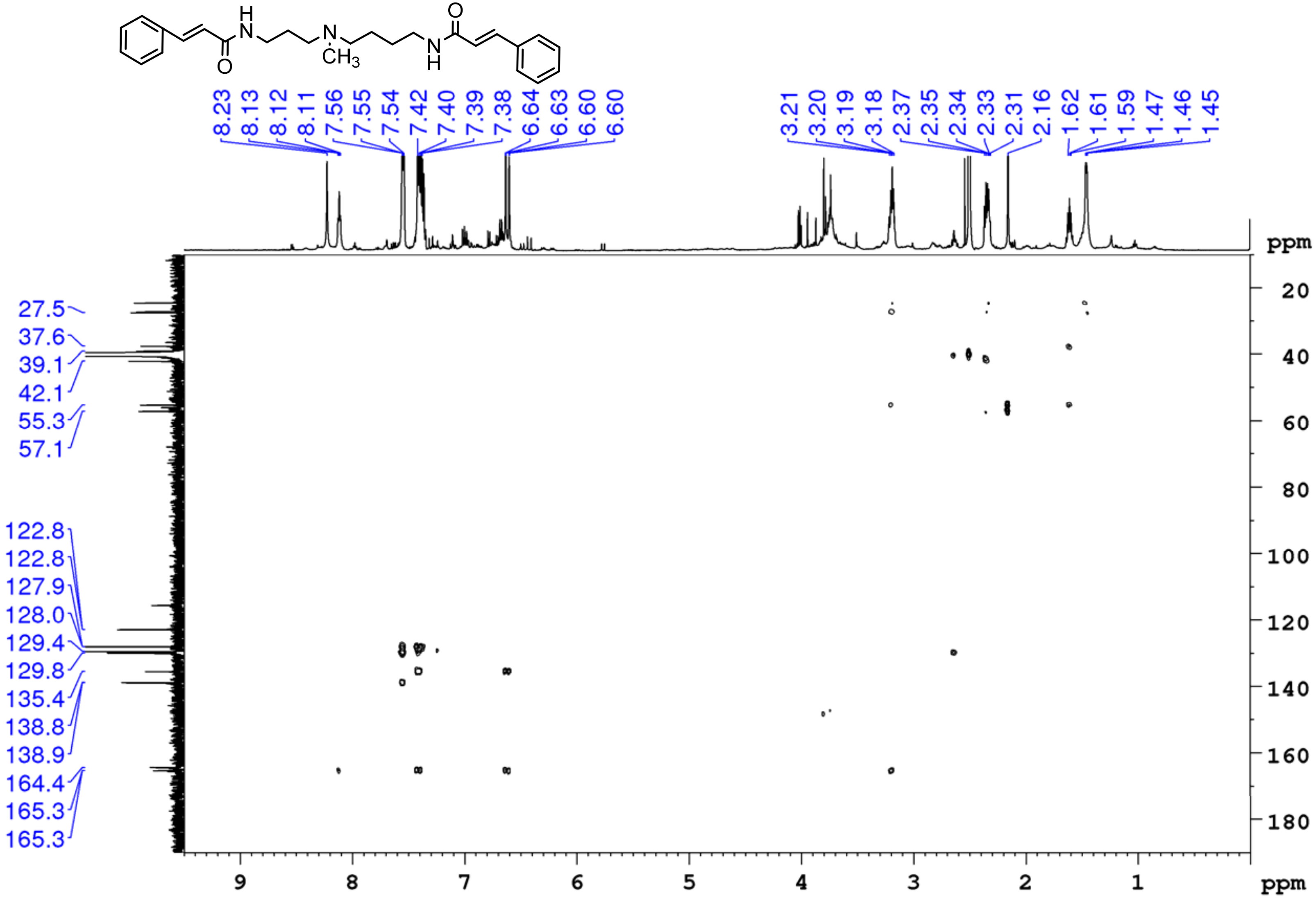

S9 Fig. HMBC spectrum (125 MHz, DMSO d_6, TMS) of 5-*N*-methylmaytenine.

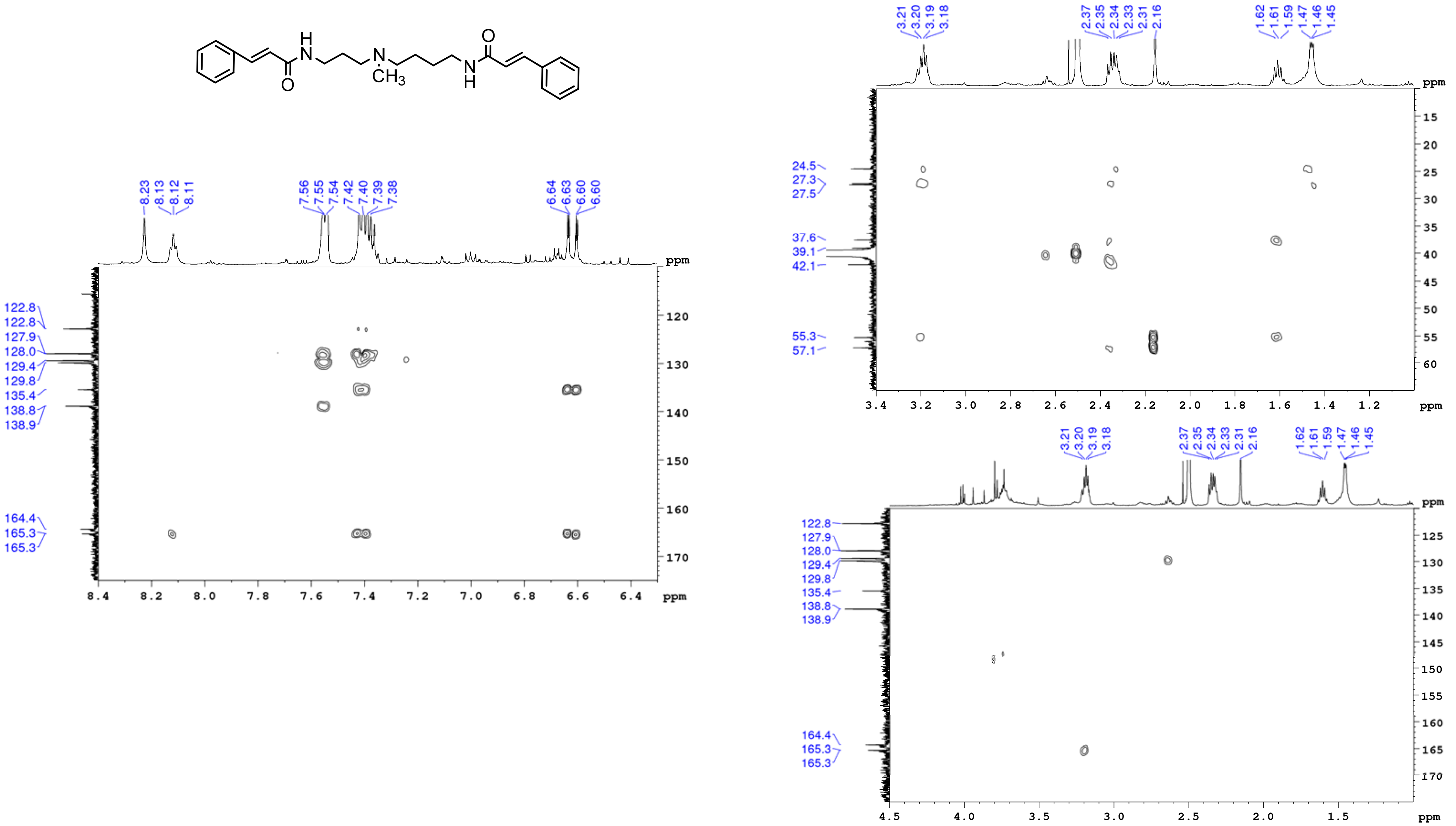

S10 Fig. Expanded regions of the HMBC spectrum (125 MHz, DMSO d_6, TMS) of 5-*N*-methylmaytenine.

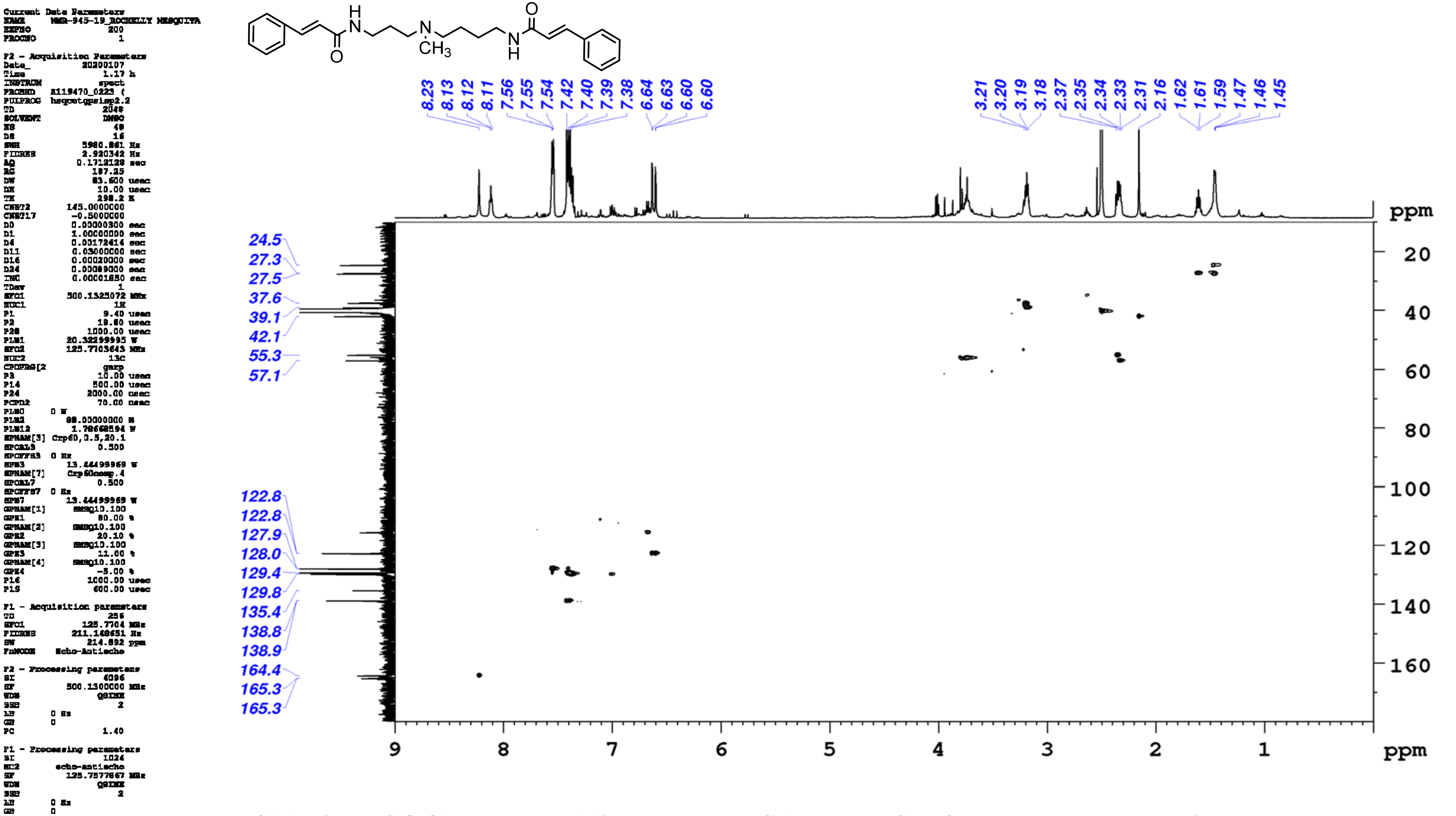

S11 Fig. HSQC spectrum (125 MHz, DMSO d$_6$, TMS) of 5-*N*-methylmaytenine.

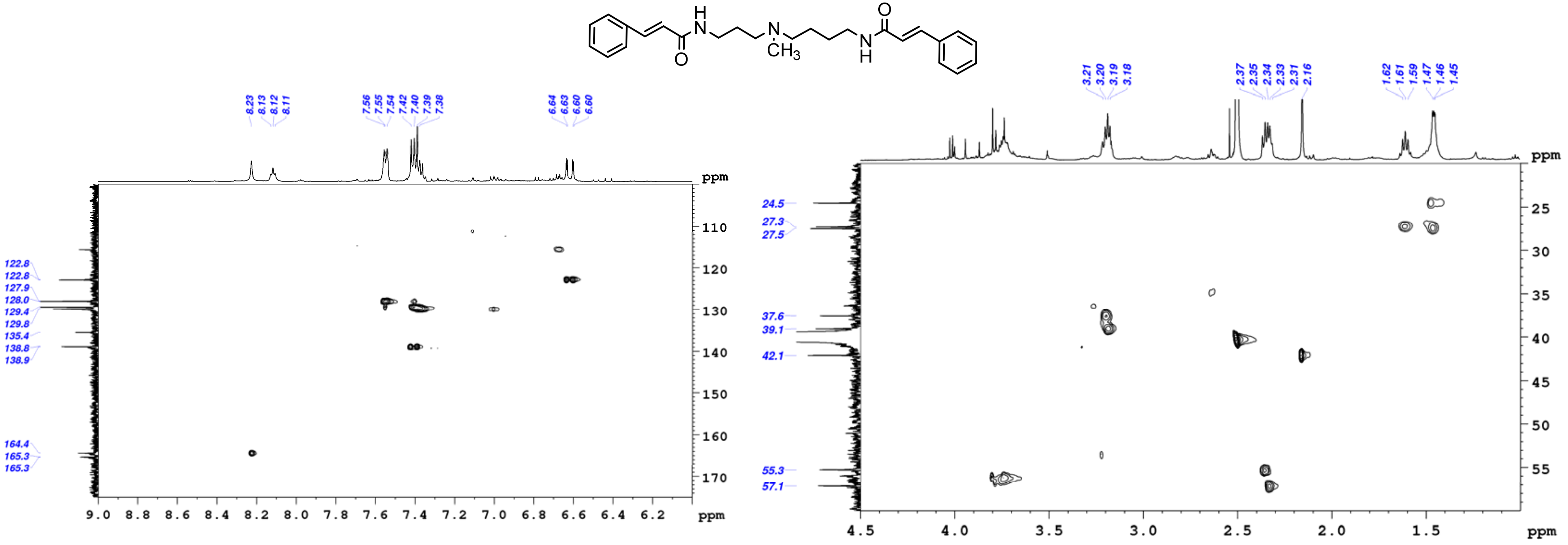

S12 Fig. Expanded regions of the HSQC spectrum (125 MHz, DMSO d_6, TMS) of 5-*N*-methylmaytenine.

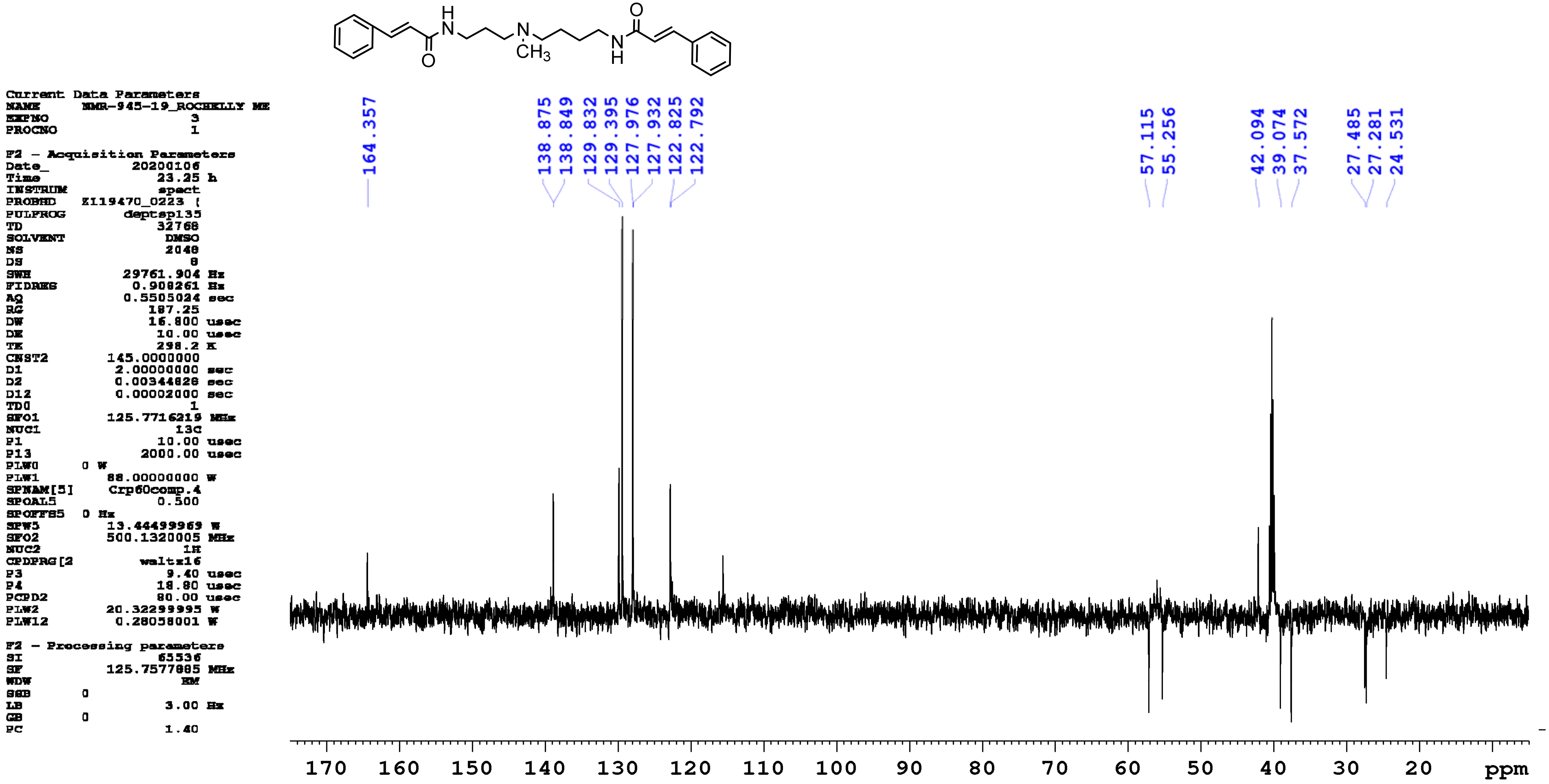

S13 Fig. DEPT-135º spectrum (125 MHz, DMSO d_6, TMS) of 5-*N*-methylmaytenine.

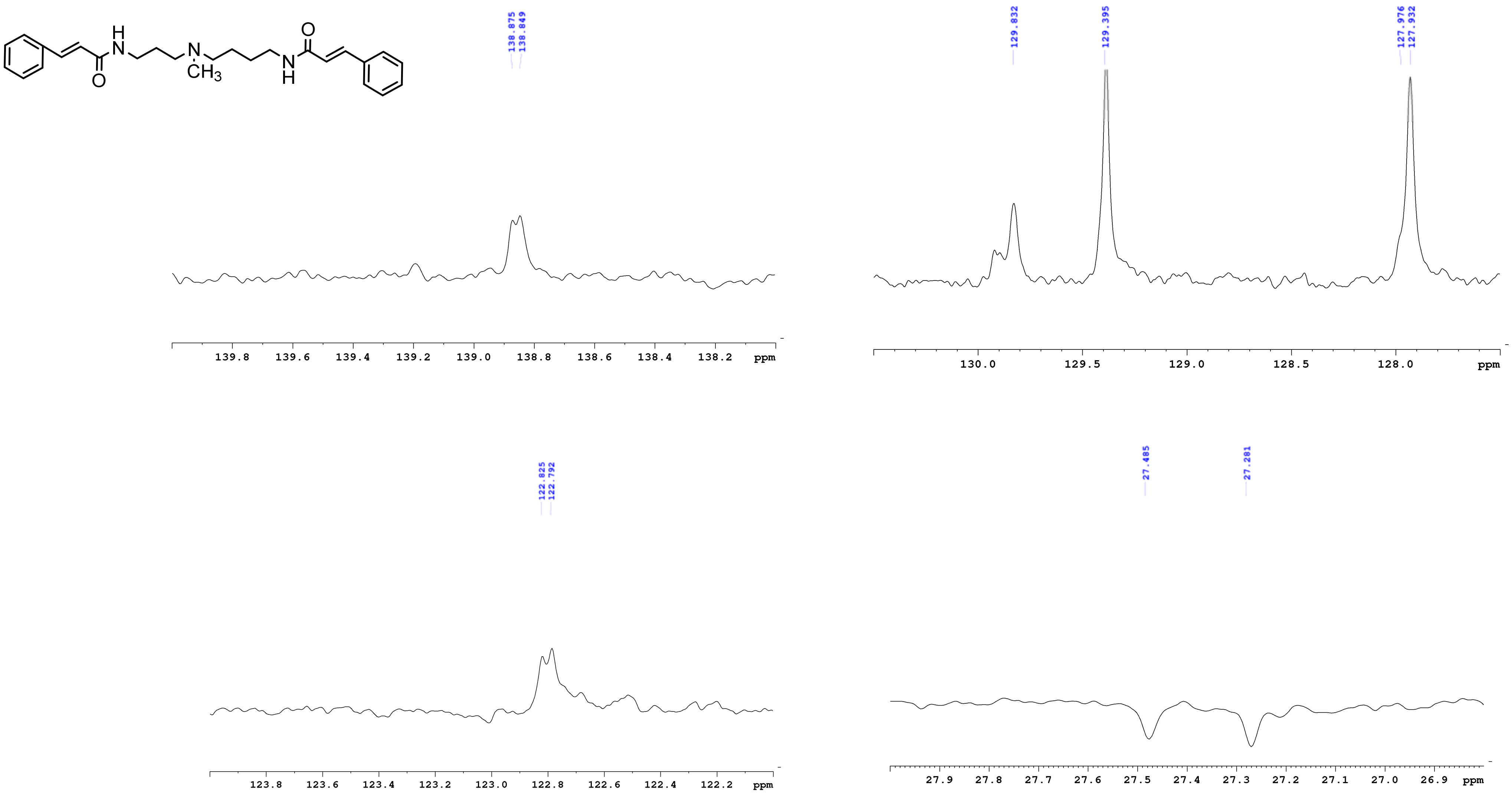

S14 Fig. Expanded regions of the DEPT-135º spectrum (125 MHz, DMSO d_6, TMS) of 5-*N*-methylmaytenine.

Current Data Parameters
NAME NMR-945-19_ROCHELLY ME
EXPNO 100
PROCNO 1

F2 - Acquisition Parameters
Date_ 20200106
Time 20.12 h
INSTRUM spect
PROBHD Z119470_0223 (
PULPROG cosygpppqf
TD 2048
SOLVENT DMSO
NS 40
DS 16
SWH 5980.861 Hz
FIDRES 2.920342 Hz
AQ 0.1712128 sec
RG 187.25
DW 83.600 usec
DE 10.00 usec
TE 298.1 K
D0 0.00000300 sec
D1 1.00000000 sec
D11 0.03000000 sec
D12 0.00002000 sec
D13 0.00000400 sec
D16 0.00020000 sec
IN0 0.00016720 sec
TDav 1
SFO1 500.1325072 MHz
NUC1 1H
P0 9.40 usec
P1 9.40 usec
P17 2500.00 usec
PLW1 20.32299995 W
PLW10 1.99530005 W
GPNAM[1] SMSQ10.100
GPZ1 10.00 %
P16 1000.00 usec

F1 - Acquisition parameters
TD 128
SFO1 500.1325 MHz
FIDRES 93.450958 Hz
SW 11.959 ppm
FnMODE QF

F2 - Processing parameters
SI 4096
SF 500.1300000 MHz
WDW QSINE
SSB 0
LB 0 Hz
GB 0
PC 1.40

F1 - Processing parameters
SI 1024
MC2 QF
SF 500.1300000 MHz
WDW QSINE
SSB 0
LB 0 Hz
GB 0

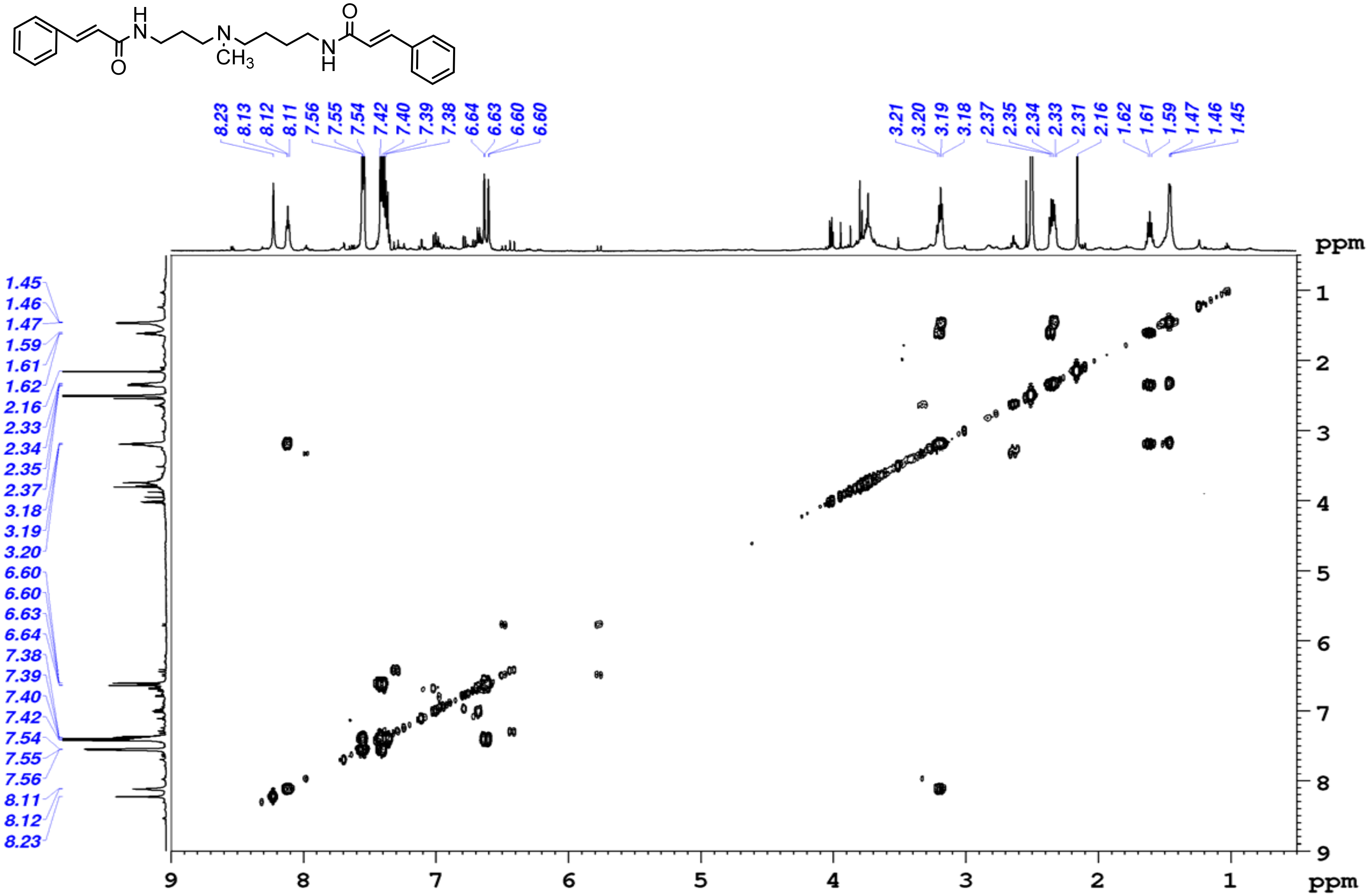

S15 Fig. COSY spectrum (500 MHz, DMSO d_6, TMS) of 5-*N*-methylmaytenine.

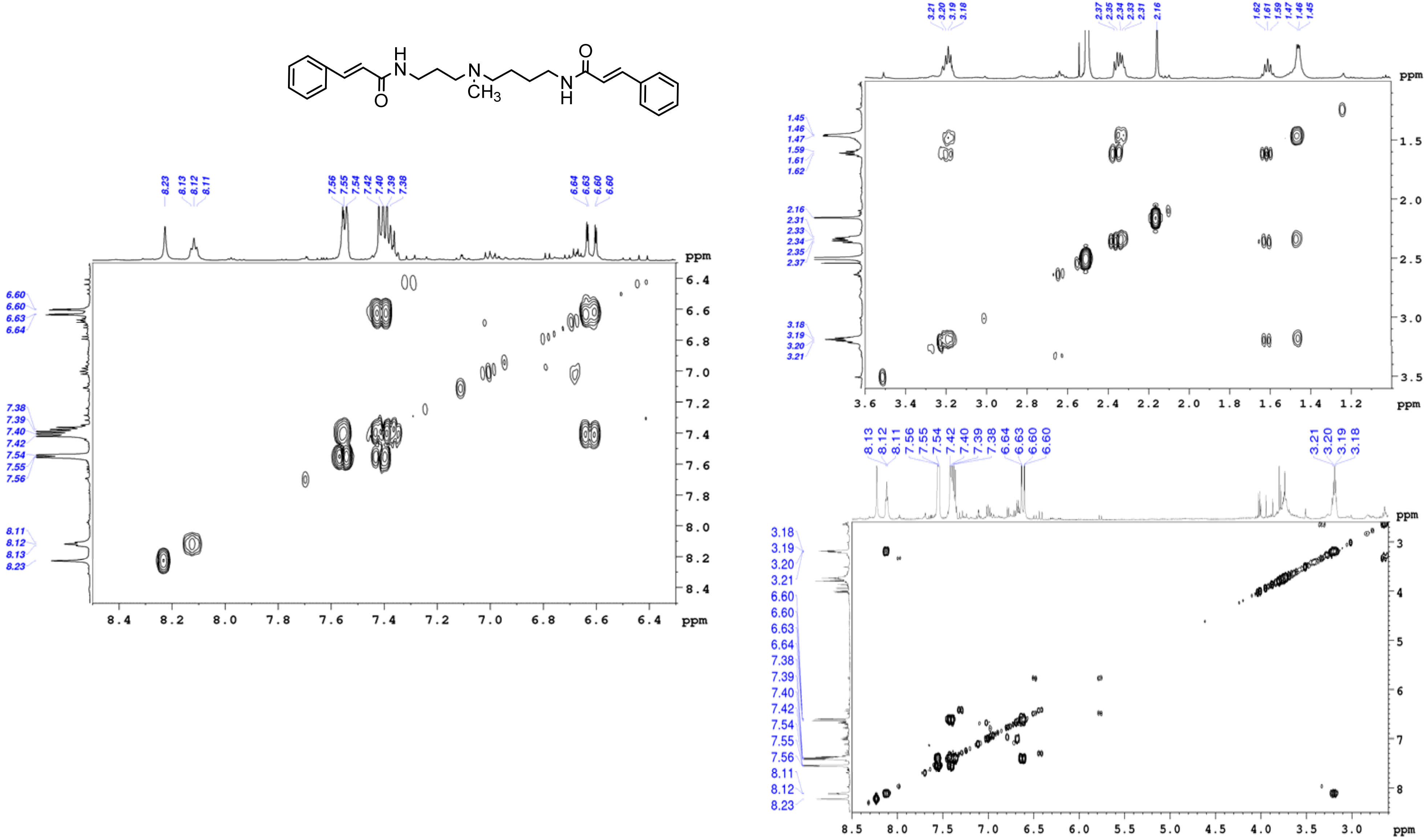

S16 Fig. Expanded regions of the COSY spectrum (500 MHz, DMSO d_6, TMS) of 5-*N*-methylmaytenine.

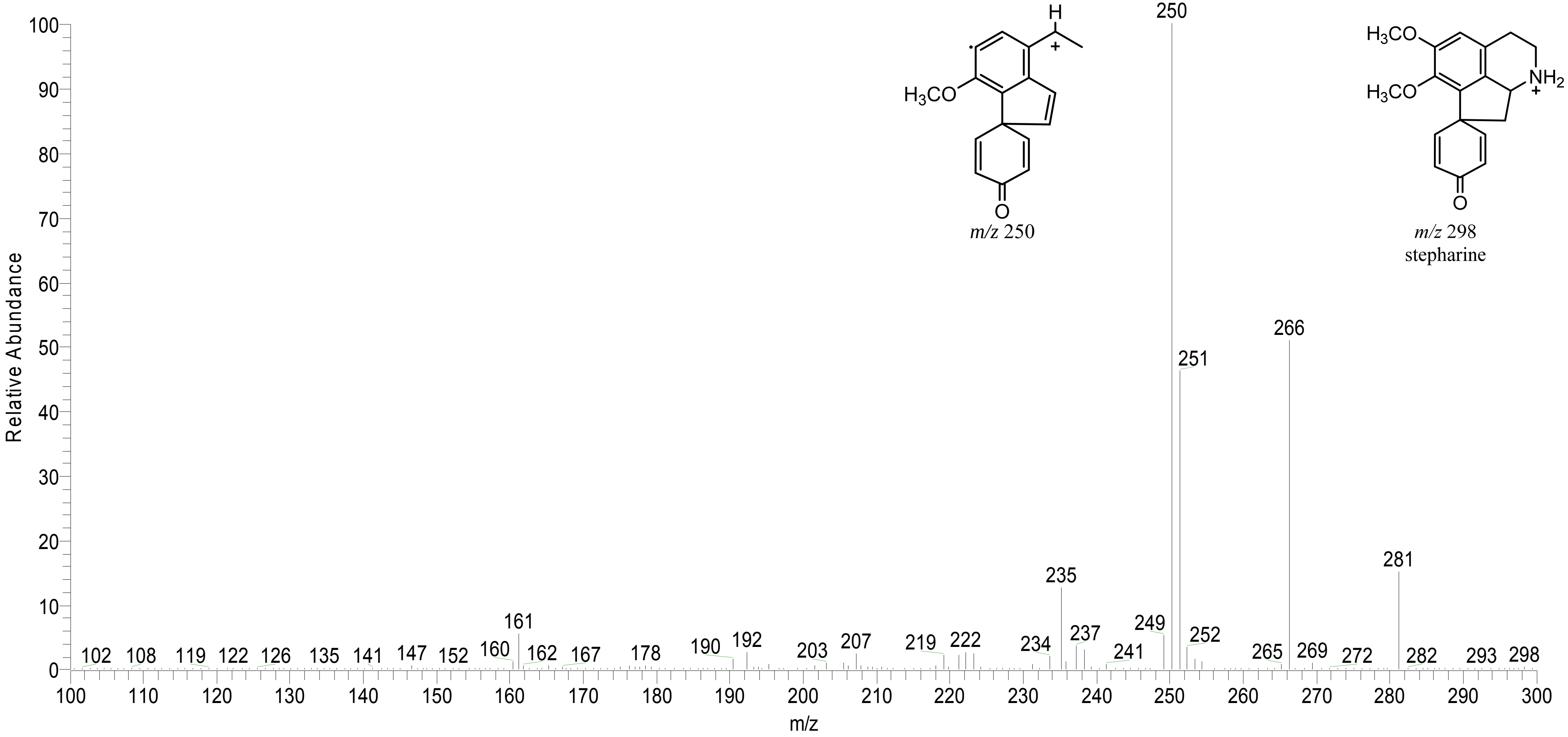

S17 Fig. MS/MS mass spectrum of stepharine with chemical structure and identification of the main fragment ions.

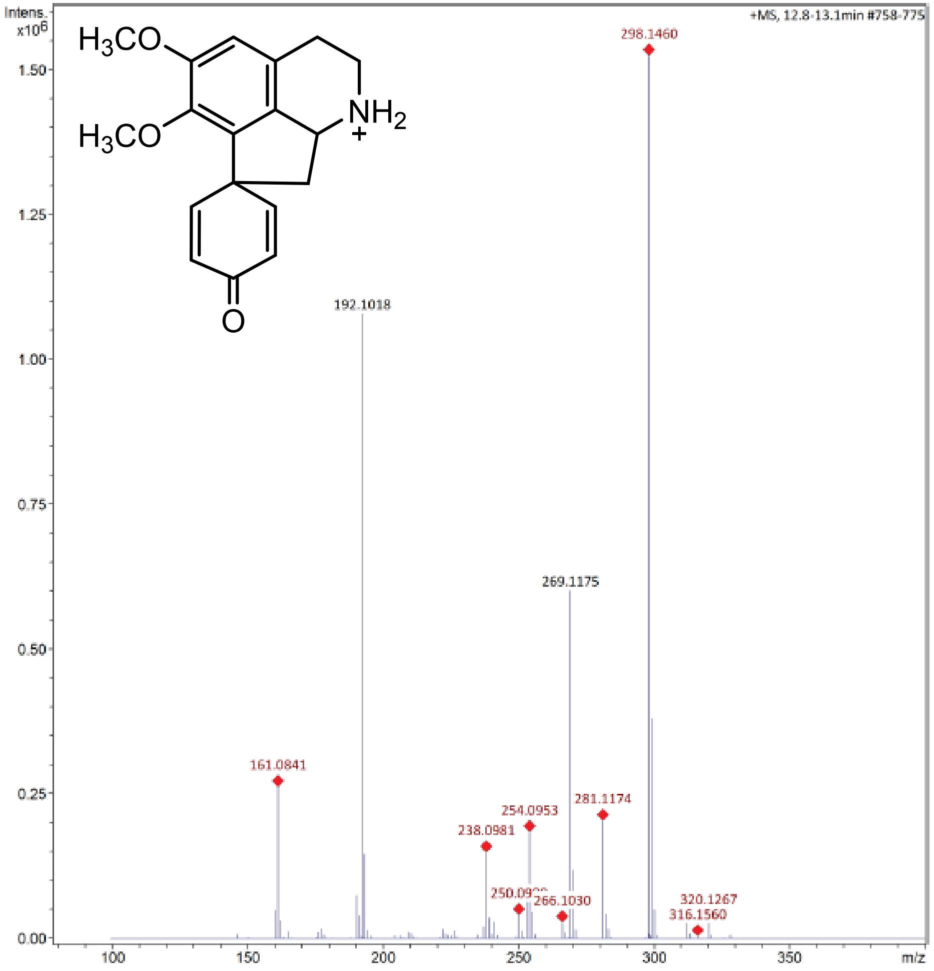

S18 Fig. Hight resolution mass spectrum of stepharine with chemical structure.

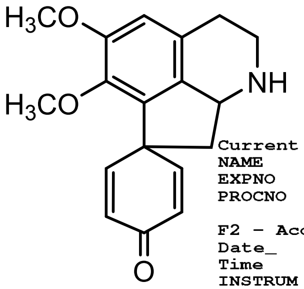

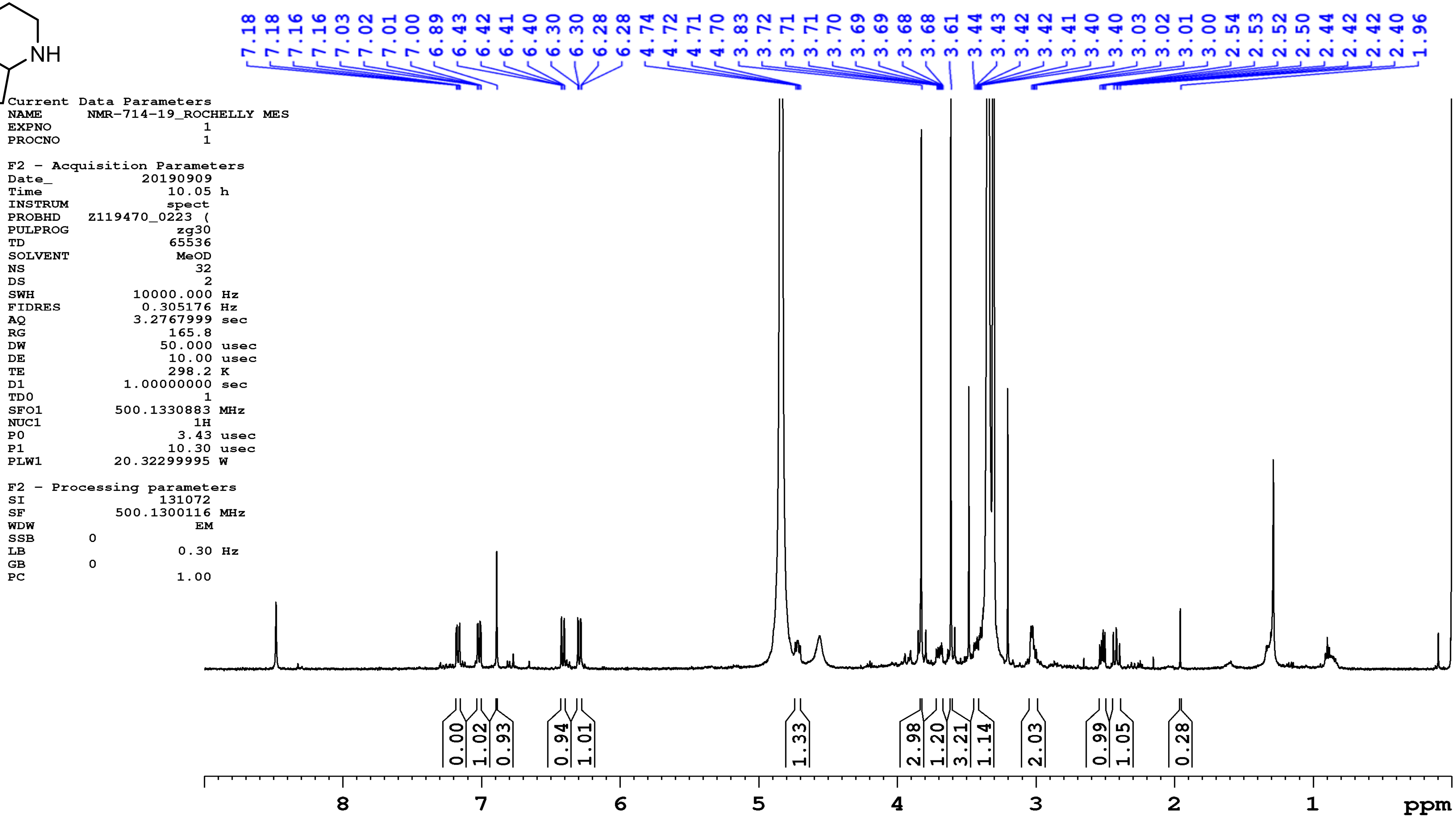

S19 Fig. ^{1}H NMR spectrum (500 MHz, CD_3OD, TMS) of stepharine.

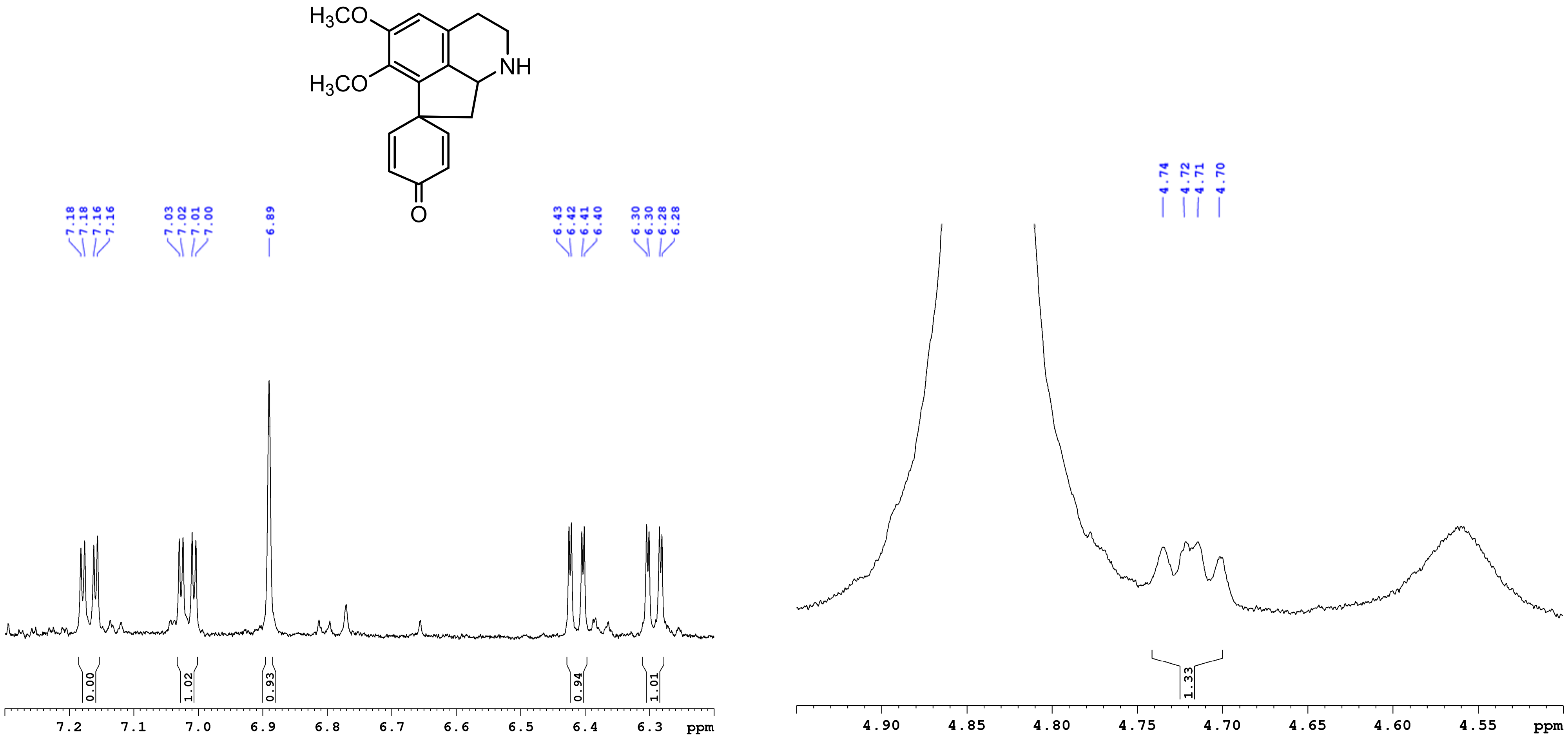

S20 Fig. Expanded regions of the ^{1}H NMR spectrum (500 MHz, CD_3OD, TMS) of stepharine.

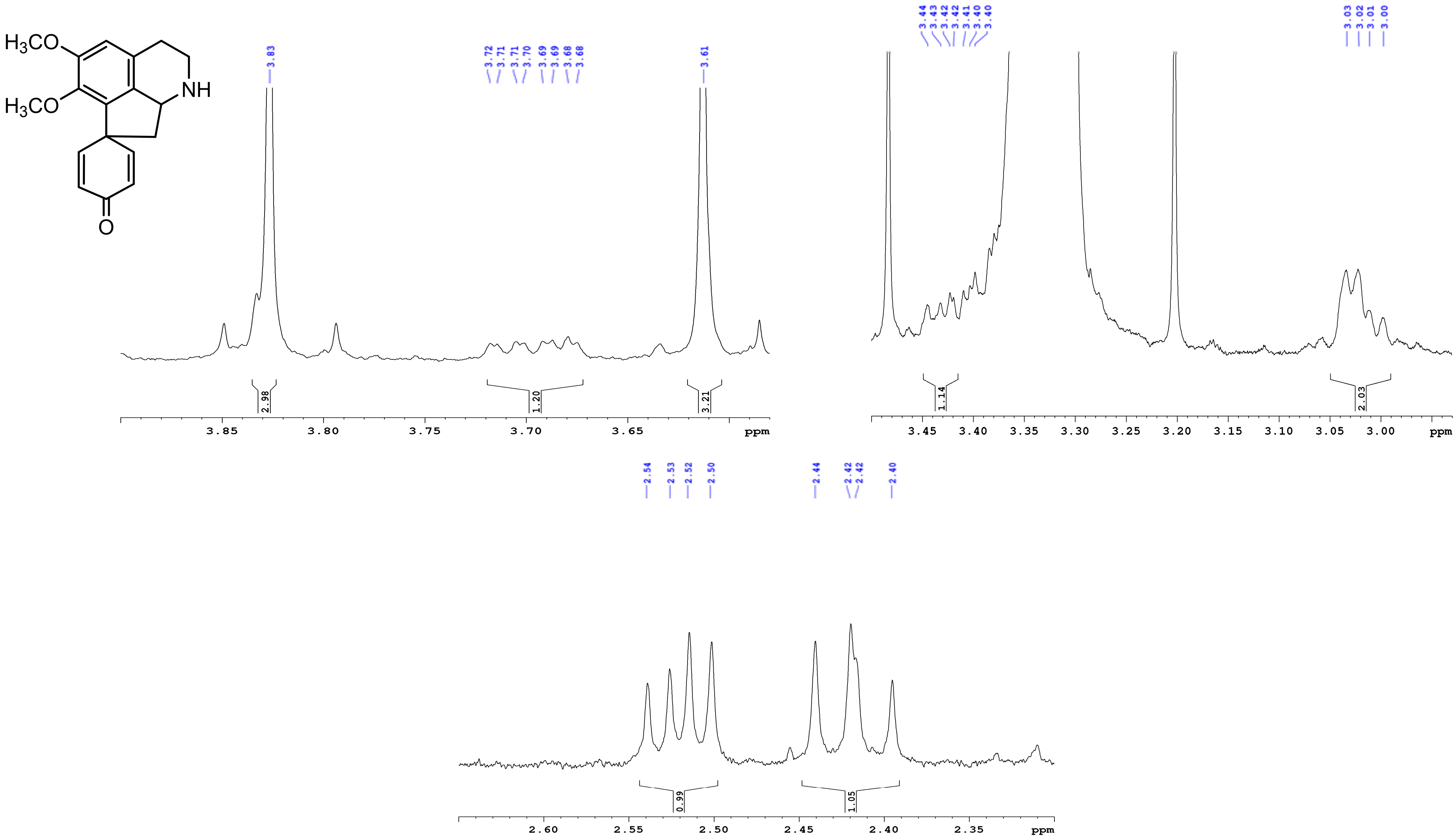

S21 Fig. Expanded regions of the ^{1}H NMR spectrum (500 MHz, CD_3OD, TMS) of stepharine.

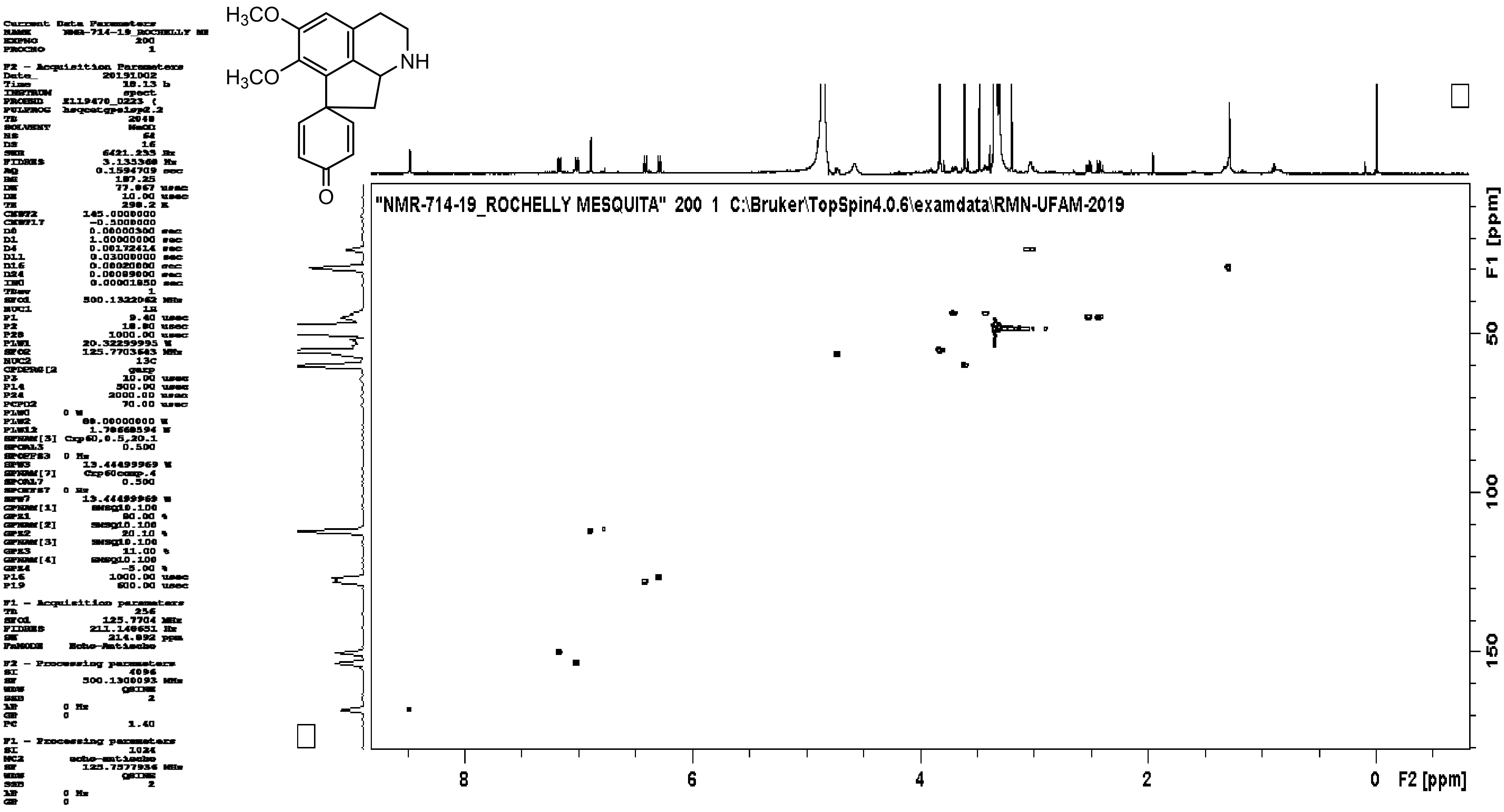

S22 Fig. HSQC spectrum (125 MHz, CD_3OD, TMS) of stepharine.

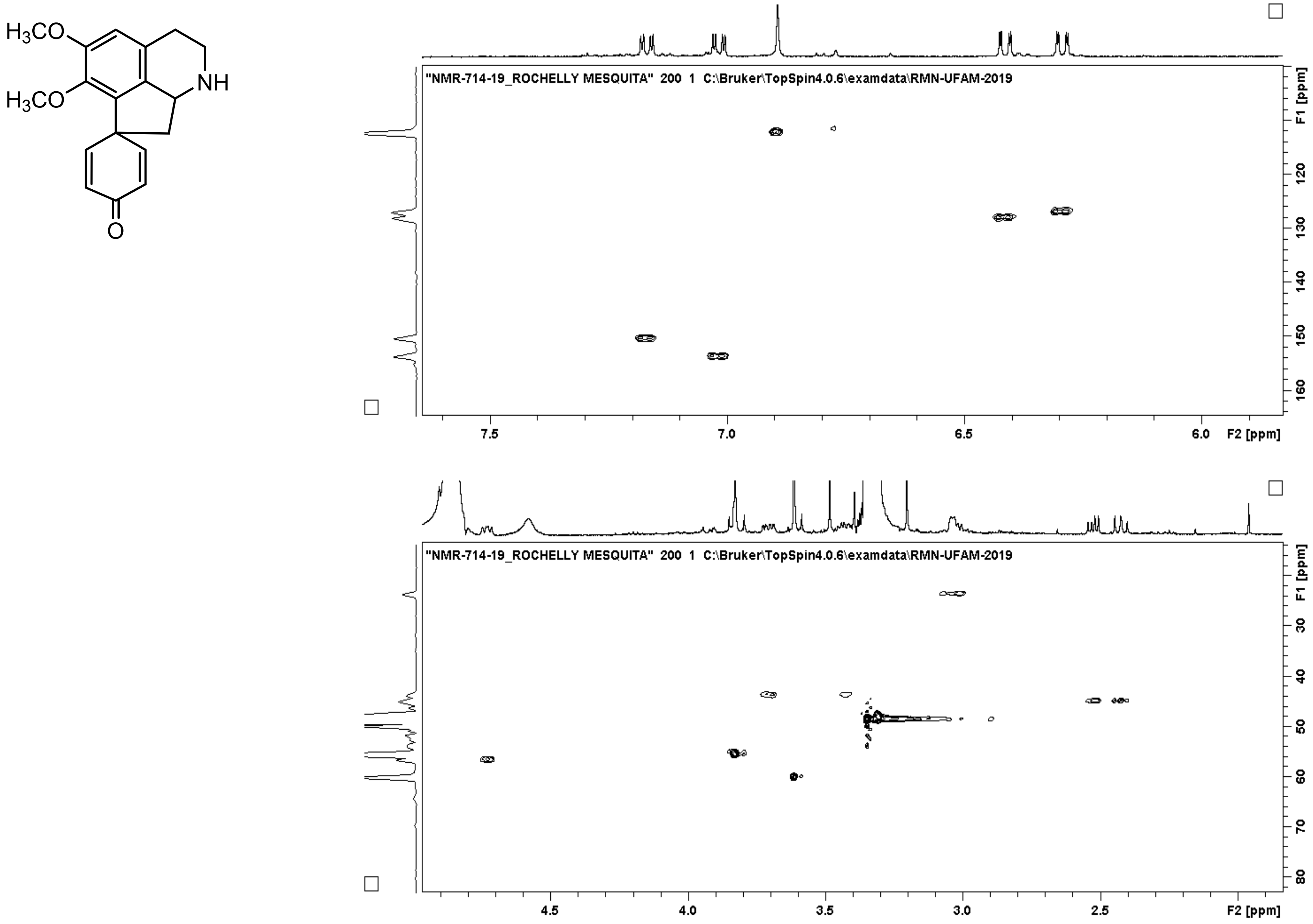

S23 Fig. Expanded regions of the HSQC spectrum (125 MHz, CD_3OD, TMS) of stepharine.

```
Current Data Parameters
NAME        NMR-714-19_ROCHELLY
EXPNO                     300
PROCNO                      1

F2 - Acquisition Parameters
Date_              20191002
Time                  23.39 h
INSTRUM               spect
PROBHD     Z119470_0223 (
PULPROG        hmbcgplpndqf
TD                     2048
SOLVENT                MeOD
NS                      186
DS                       16
SWH             6421.233 Hz
FIDRES          3.135368 Hz
AQ             0.1594709 sec
RG                   187.25
DW                   77.867 usec
DE                    10.00 usec
TE                    298.2 K
CNST2           145.0000000
CNST13            8.0000000
D0               0.00000300 sec
D1               1.00000000 sec
D2               0.00344828 sec
D6               0.06250000 sec
D16              0.00020000 sec
IN0              0.00001660 sec
TDav                      1
SFO1         500.1322062 MHz
NUC1                     1H
P1                     9.40 usec
P2                    18.80 usec
PLW1            20.32299995 W
SFO2         125.7716219 MHz
NUC2                    13C
P3                    10.00 usec
PLW2            88.00000000 W
GPNAM[1]         SMSQ10.100
GPZ1                  50.00 %
GPNAM[2]         SMSQ10.100
GPZ2                  30.00 %
GPNAM[3]         SMSQ10.100
GPZ3                  40.10 %
P16                 1000.00 usec

F1 - Acquisition parameters
TD                      240
SFO1            125.7716 MHz
FIDRES        251.004013 Hz
SW               239.486 ppm
FnMODE                   QF

F2 - Processing parameters
SI                     4096
SF           500.1300128 MHz
WDW                    SINE
SSB        0
LB         0 Hz
GB         0
PC                     1.40

F1 - Processing parameters
SI                     1024
MC2                      QF
SF           125.7578149 MHz
WDW                    SINE
SSB        0
LB         0 Hz
GB         0
```

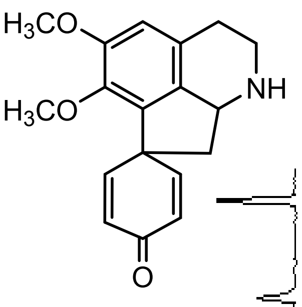

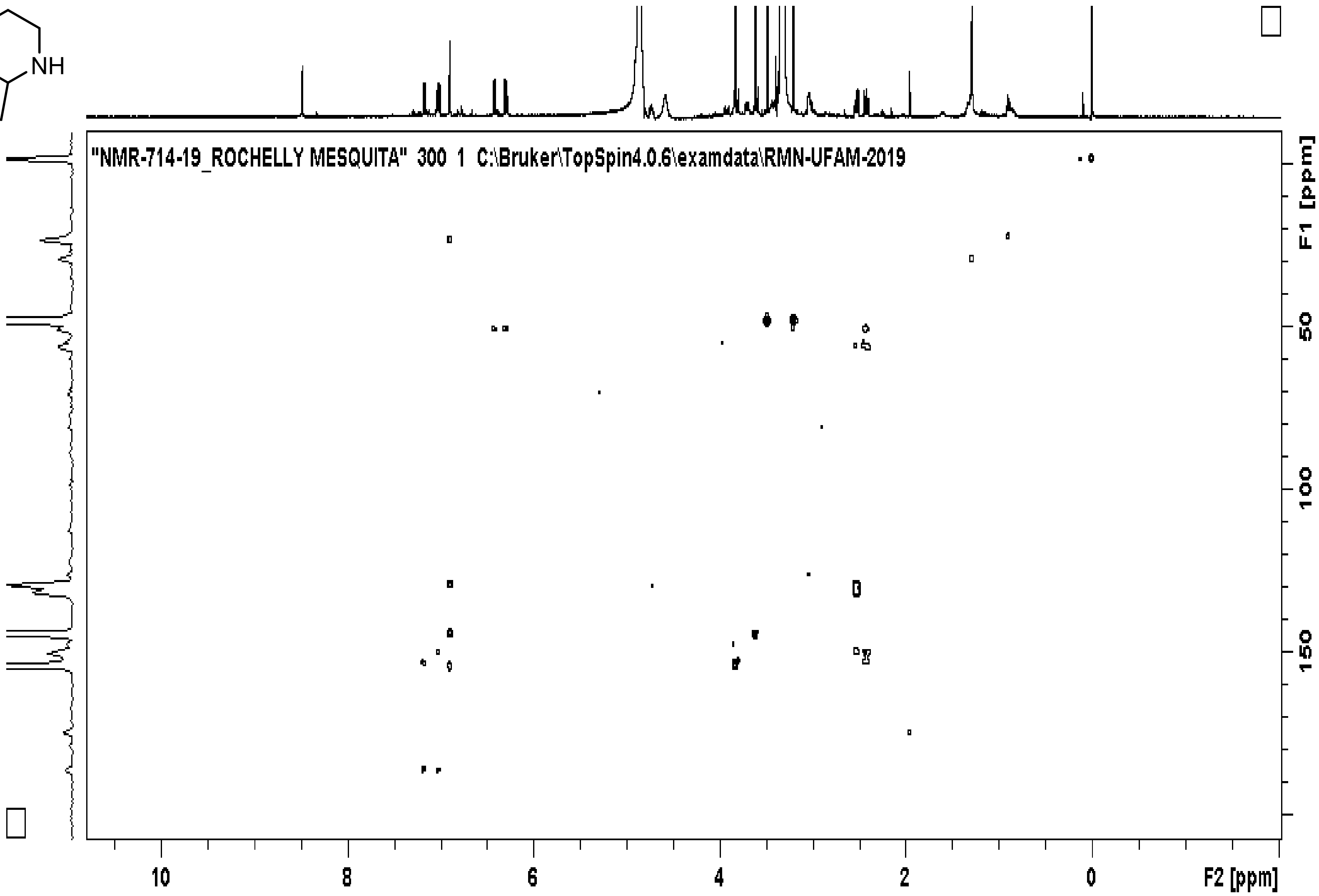

S24 Fig. HMBC spectrum (125 MHz, CD_3OD, TMS) of stepharine.

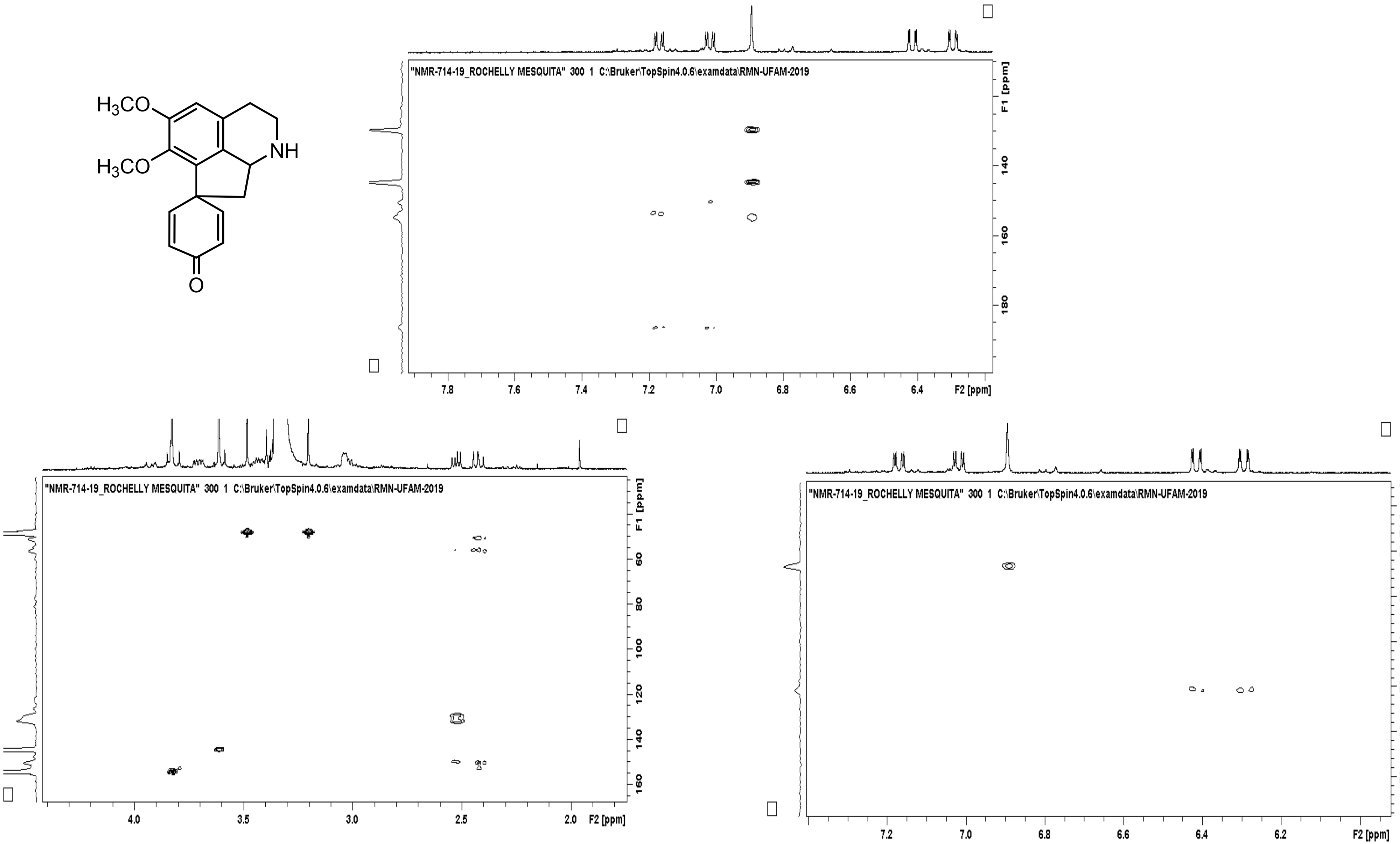

S25 Fig. Expanded regions of the HMBC spectrum (125 MHz, CD_3OD, TMS) of stepharine.

Current Data Parameters
NAME NMR-714-19_ROCHELLY ME
EXPNO 100
PROCNO 1

F2 - Acquisition Parameters
Date_ 20191002
Time 16.49 h
INSTRUM spect
PROBHD Z119470_0223 (
PULPROG cosygpppqf
TD 2048
SOLVENT MeOD
NS 32
DS 8
SWH 6421.233 Hz
FIDRES 3.135368 Hz
AQ 0.1594709 sec
RG 187.25
DW 77.867 usec
DE 10.00 usec
TE 298.2 K
D0 0.00000300 sec
D1 1.00000000 sec
D11 0.03000000 sec
D12 0.00002000 sec
D13 0.00000400 sec
D16 0.00020000 sec
IN0 0.00015580 sec
TDav 1
SFO1 500.1322062 MHz
NUC1 1H
P0 9.40 usec
P1 9.40 usec
P17 2500.00 usec
PLW1 20.32299995 W
PLW10 1.99530005 W
GPNAM[1] SMSQ10.100
GPZ1 10.00 %
P16 1000.00 usec

F1 - Acquisition parameters
TD 128
SFO1 500.1322 MHz
FIDRES 100.288834 Hz
SW 12.834 ppm
FnMODE QF

F2 - Processing parameters
SI 4096
SF 500.1300000 MHz
WDW QSINE
SSB 0
LB 0 Hz
GB 0
PC 1.40

F1 - Processing parameters
SI 1024
MC2 QF
SF 500.1300000 MHz
WDW QSINE
SSB 0
LB 0 Hz
GB 0

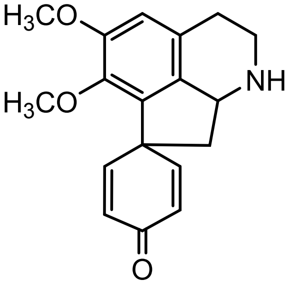

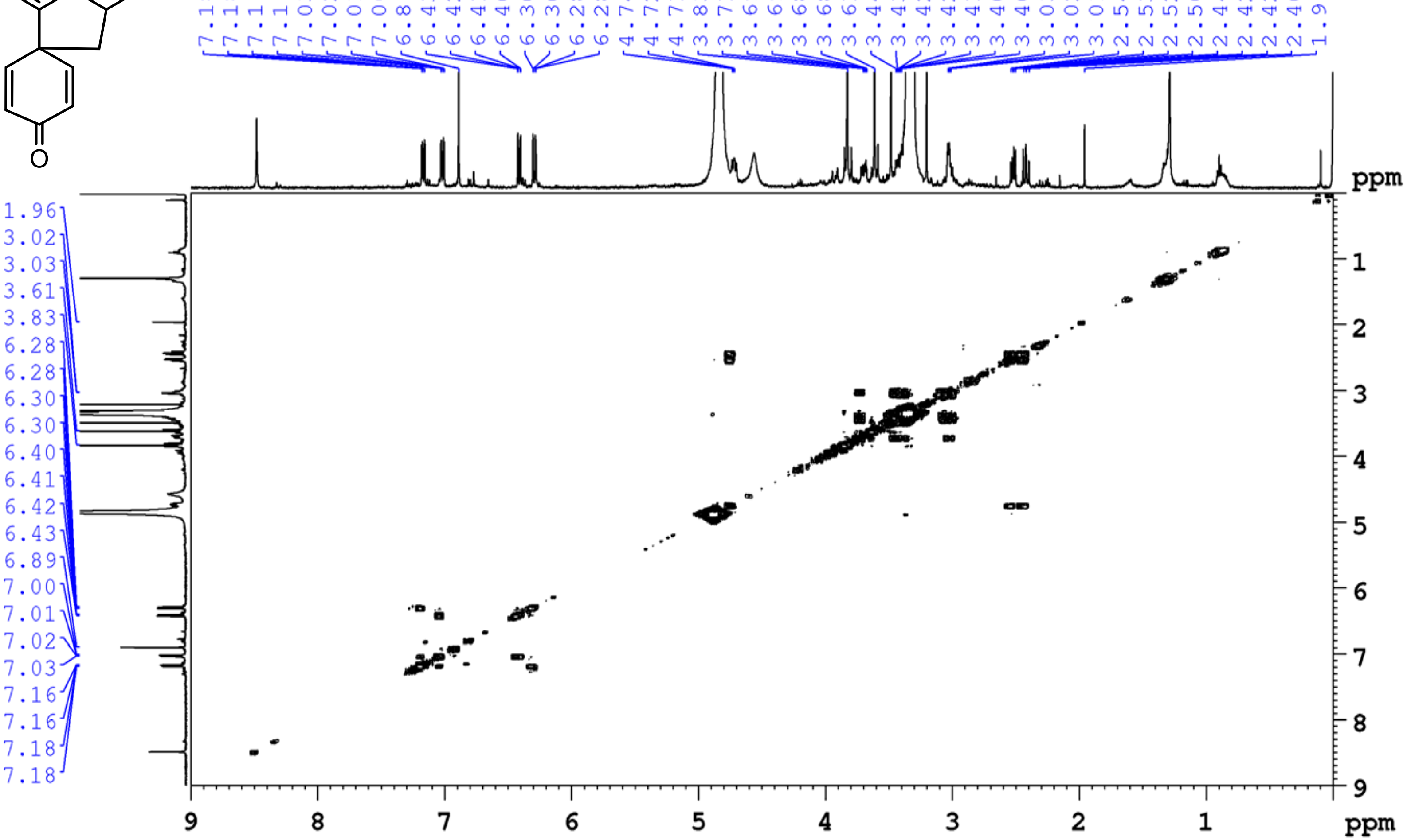

S26 Fig. COSY spectrum (500 MHz, CD_3OD, TMS) of stepharine.

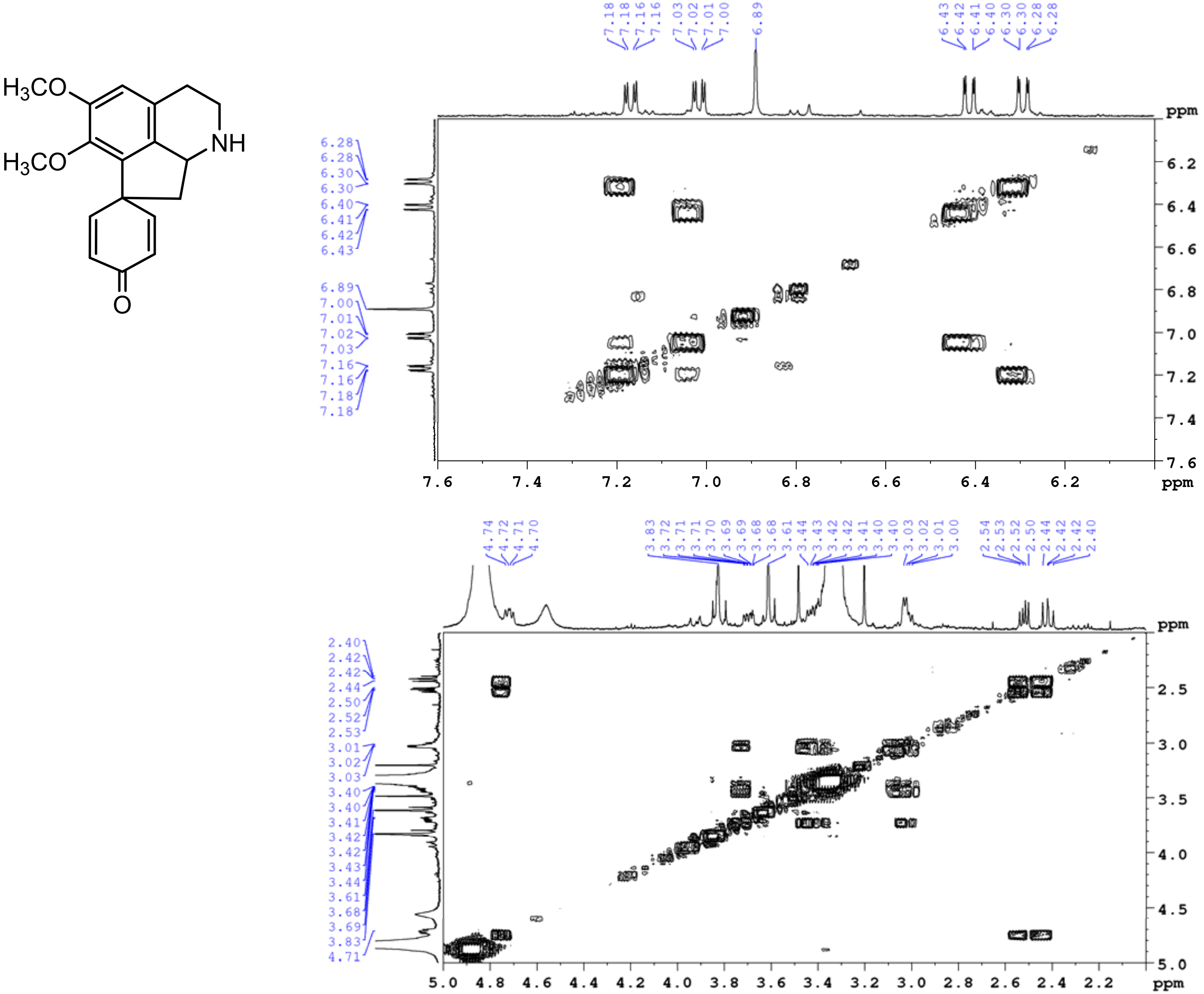

S27 Fig. Expanded regions of the COSY spectrum (500 MHz, CD_3OD, TMS) of stepharine.

S1 Table. NMR chemical shifts of 5-*N*-methylmaitenine. The experiments were realized in DMSO d_6 at magnetic field strength of 11.7 T; 500 MHz for ^{1}H and 125 MHz for ^{13}C NMR.

s: singlet; d: doublet; t: triplet; m: multiplet.

Position	^{1}H		^{13}C	
	δ_H, ppm (multiplicity; number of H; *J*, Hz)	COSY, ppm	δ_C, ppm	HMBC, ppm
4´ and 4´´	-	-	135.43	-
1´ or 1´´	-	-	165.27	-
1´´ or 1´	-	-	165.32	-
5´ and 5´´ or 9´ and 9´´	7.56 – 7.54 (m; 2H)	H-3’; H-7’; H-3”; H-7”	127.98	C-3’; C-3”; C-7’; C-7”; C-9’; C-9” or C-5’; C-5”
9´ and 9´´ or 5´ and 5´´	7.56 – 7.54 (m; 2H)	H-3’; H-3”; H-7’; H-7”	127.94	C-3’; C-3”; C-7’; C-7”; C-5’; C-5” or C-9’; C-9”
6´, 6´´, 8´and 8´´	7.56 – 7.54 (m; 4H)	H-3’; H-3”; H-7’; H-7”	129.39	C-3’; C-3”; C-7’; C-7”
7´ and 7´´	7.40 (m; 2H)	H-5’; H-5” H-6’; H-6” H-9’; H-9”	129.83	C-5’; C-5”; C-9’; C-9”
3´or 3´´	7.42 (m; 1H)	H-2’; H-2” H-5’; H-5” H-6’; H-6” H-9’; H-9”	138.85	C-1’; C-1”; C-2’; C-2”; C-4’; C-4”; C-5’; C-5”; C-9’; C-9”
3´´or 3´	7.38 (m; 1H)	H-2’; H-2” H-5’; H-5” H-6’; H-6” H-9’; H-9”	138.88	C-1’; C-1”; C-2’; C-2”; C-4’; C-4”; C-5’; C-5”; C-9’; C-9”
2´or 2´´	6.63 (d; 1H; 16 Hz)	H-3’; H-3”	122.79	C-1’; C-1”; C-4’; C-4”
2´´ or 2´	6.60 (d; 1H; 16 Hz)	H-3’; H-3”	122.83	C-1’; C-1”; C-4’; C-4”
1 and 10 N-H	8.12 (t; 5.5 Hz)	H-2; H-9	-	C-1’; C-1”
2	3.20 (m; 2H)	H-3; -NH	37.58	C-1’; C-1”; C-3; C-4
9	3.18 (m; 2H)	H-8; -NH	39.07	C-1’; C-1”; C-8
4	2.35 (m; 2H)	H-3	55.26	C-2; C-3; C-5; C-6
6	2.32 (m; 2H)	H-7	57.12	C-4; C-8
5 N-CH_3	2.16 (s; 3H)	-	42.09	C-4; C-6
3	1.61 (m; 2H)	H-2; H-4	27.28	C-4; C-2
8	1.47 (m; 2H)	H-9	24.53	C-7
7	1.45 (m; 2H)	H-6	27.48	C-8

S2 Table. NMR chemical shifts of stepharine. The experiments were realized in CD_3OD at magnetic field strength of 11.7 T; 500 MHz for ^1H NMR.

s: singlet; m: multiplet; dd: doublet of doublets; ddd: doublet of doublets of doublets.

**Position**	**^1H**	**^{13}C**	
	**δ_H, ppm (multiplicity; number of H; *J*, Hz)**	**δ_C, ppm [a]**	**HMBC, ppm [a]**
1	-	144.6	-
1 -OCH_3	3.61 (s; 3H)	59.9	C-1
1a	-	133.8	-
2	-	154.7	-
2 -OCH_3	3.82 (s ; 3H)	55.4	C-2
3	6.89 (s ; 1H)	112.1	C-1; C-2; C-3b; C-4
3a	-	131.5	-
3b	-	129.3	-
4	3.00 (m; 2H)	23.5	-
5	3.44 (m; 1H)<br>3.69 (ddd; 1H; 13, 5.9, and 2.3 Hz)	43.7	-
6 -NH	1.95 (s)	-	-
6a	4.72 (m; 1H)	56.5	C-1a
7	2.42 (dd; 1H; 12 and 10.5 Hz)<br>2.52 (dd; 1H; 12 and 6.5 Hz)	44.9	C-1a; C-3b; C-6a; C-7a; C-8; C-12
7a	-	50.7	-
8	7.16 (dd; 1H; 10 and 3 Hz)	150.3	C-7; C-7a; C-10
9	6.29 (dd; 1H; 10 and 1.9 Hz)	126.7	C-7a
10	-	186.5	-
11	6.41 (dd; 1H; 10 and 1.9 Hz)	127.8	C-7a
12	7.02 (dd; 1H; 10 and 3 Hz)	153.6	C-10

[a] ^{13}C NMR signals were assigned using the HSQC and HMBC data

Molecular docking calculations

Ligand structures preparation

Ligand structures were initially drawn in a suitable molecular editor software and then transferred to Avogadro [1,2]. Obtained geometries were optimized by classic molecular mechanics implemented in Avogadro. Geometries were optimized using a semi-empirical PM7 level [3] within the MOPAC program [4]. Ligand structure files for docking were prepared using AutoDock Tools [5,6]. Default settings for the detection of rotatable bonds were used.

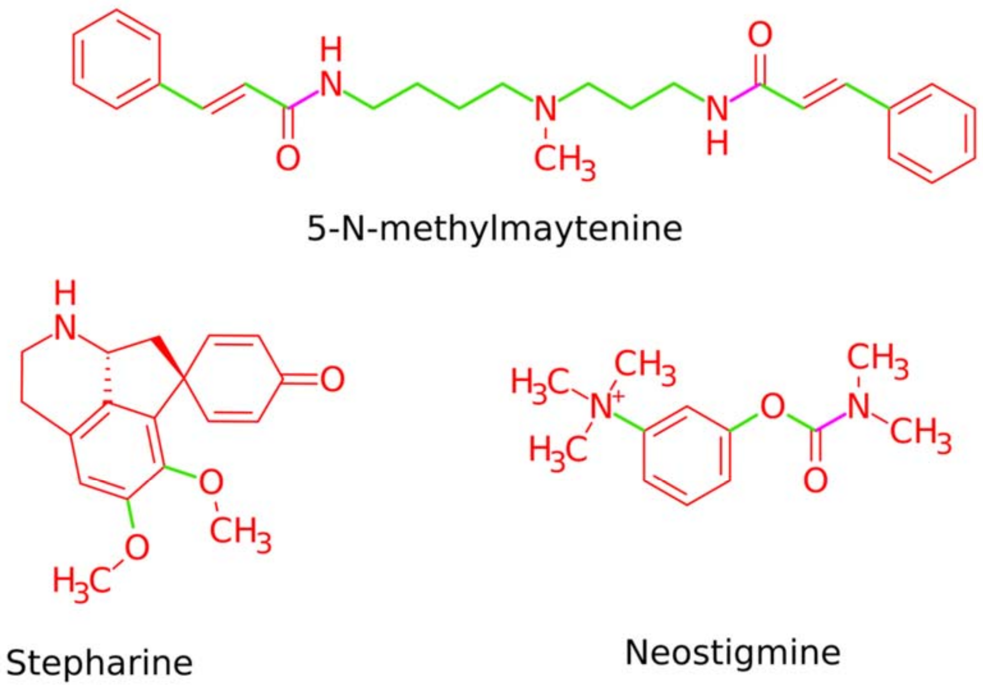

S28 Fig. Chosen rotatable bonds in the ligands under investigation. The bonds marked green are rotatable during the docking run, magenta denotes non-rotatable bonds, and red marks unrotatable bonds.

Protein files preparation

X-ray structures of the proteins under investigation (AChE, PDB ID: 6H12; IL-6, PDB ID: 4NI7 and IL-8, PDB ID: 3IL8) were obtained from the RCSB Protein Data Bank. Small molecules and water were removed. Side chains with the highest occupancy values were left, if alternative conformations were present in the corresponding .pdb files. Binding sites of the proteins were identified using Discovery Studio Visualizer [7]. Structure files for docking were prepared using AutoDock Tools [5,6].

Missing residues

Modeling of missing residues in the protein structures was performed using Modeller web-service [8]. Obtained structures that do not possess serious structural issues were selected for the docking studies. Clashes or contacts between side chains in the produced structures were resolved by minimization routine implemented in UCSF Chimera [9].

AChE (6H12)

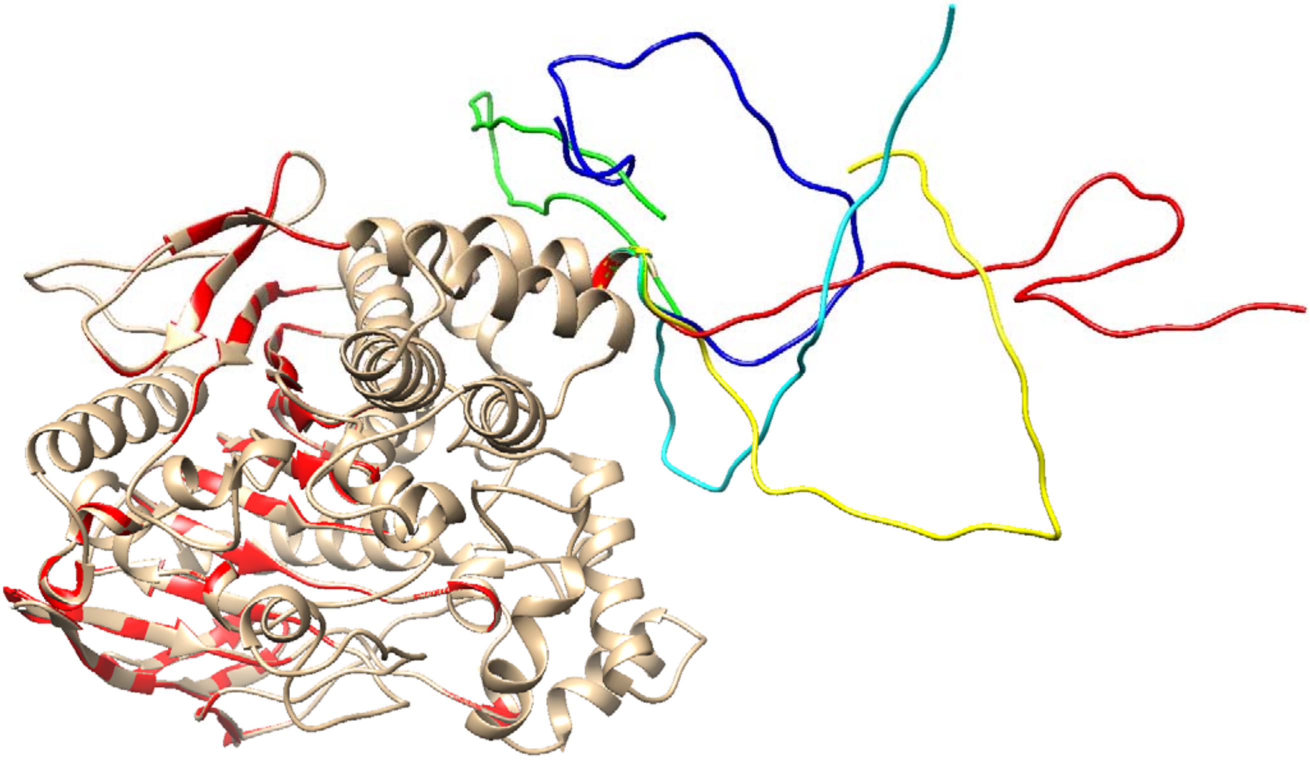

S29 Fig. Alignment of five modeled conformations of missing loops in 6H12 structure. (See S3 Table for details)

S3 Table. Scores of the obtained modeled structures.

Model	Color	ZDOPE	Estimated RMSD	Estimated overlap (3.5 Å)
1	red	-1.79	0.384	0.950
2	yellow	-1.75	0.465	0.950
3	green	-1.88	0.309	0.950
4	cyan	-1.78	0.383	0.950
5	blue	-1.89	0.347	0.950

The X-ray structure of 6H12 had missing residues Ala536-Phe565 at the C-terminal region, which were modeled. All five resulting models were free of clashes/contacts. Model #5 was chosen as the main one for docking studies based on the scores (S3 Table).

IL-6 (4NI7)

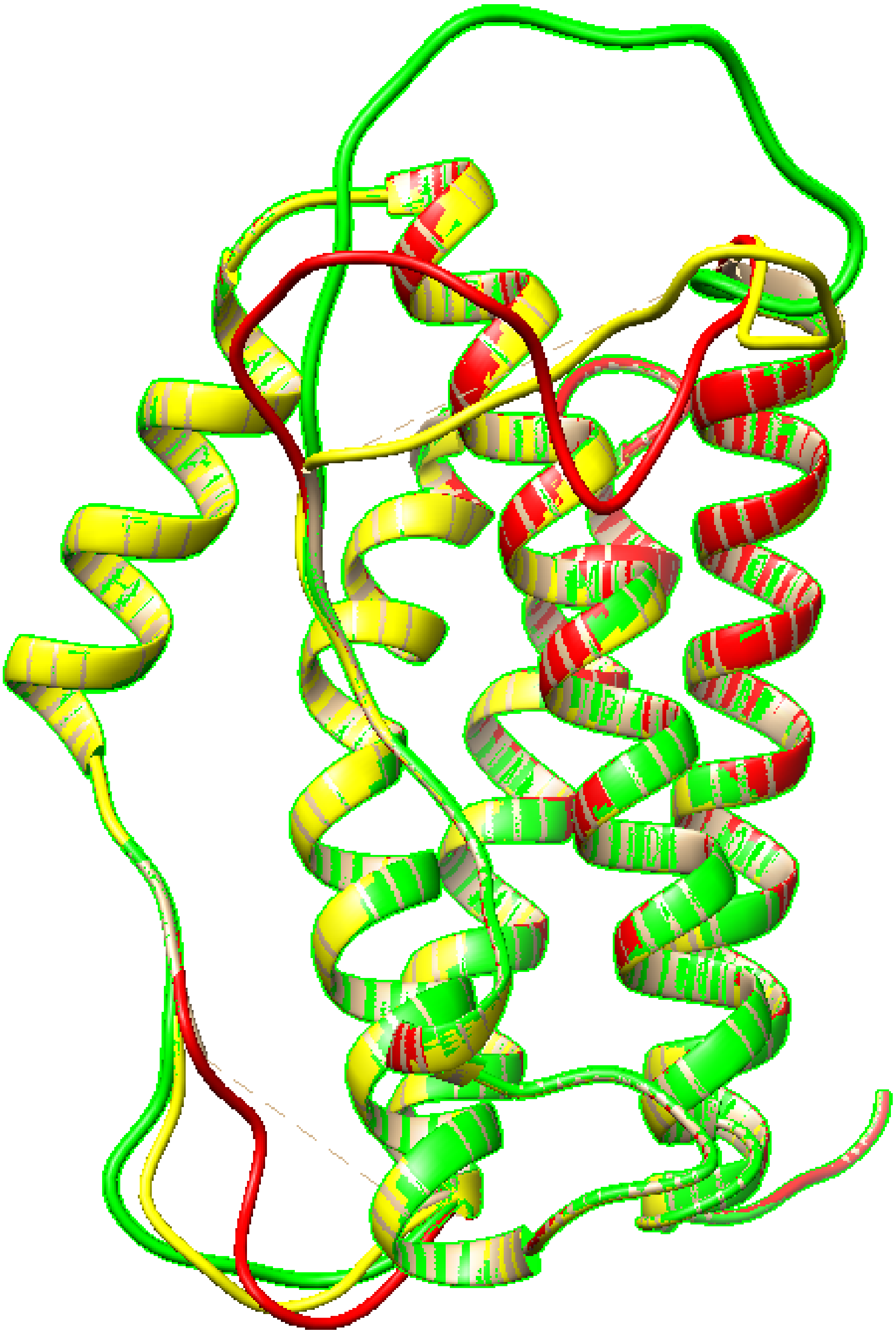

S30 Fig. Alignment of three modeled conformations of missing loops in the 4NI7 structure. (See S4 Table for details)

S4 Table. Scores of the obtained modeled structures.

Model	Color	ZDOPE	Estimated RMSD	Estimated overlap (3.5 Å)
1	red	-2.24	1.243	0.931
2	yellow	-2.19	1.519	0.923
3	green	-2.25	1.278	0.946

The structure of IL-6 published under PDB ID 4NI7 has missing residue clusters Asn48-Asn60 and Lys131-Ala135. There is also a 17 amino acid sequence missing at the N-terminus, which was not modeled in the present work. Modeling produced five conformations, two of which had structural issues and were omitted. Model #3 was chosen as the main one for the docking studies.

Water molecules

Selection of water molecules that can be important in docking was performed by the distance criterion. All molecules farther than 3.3 Å from the H-bond donors and acceptors in the protein structures were removed. Protonation of the oxygen atoms in water molecules was made in UCSF Chimera followed by minimization of hydrogen positions. Subsequently, the .pdb files were prepared, each containing the protein and one H_2O molecule. Those files were converted to .pdbqt according to AutoDock Vina manual and used further for docking.

Docking runs.

Docking studies were performed with AutoDock Vina program [10]. The protein structures stayed rigid during the docking in all cases. Number of binding poses generated in each run was 9. Since the success of a docking run depends on the random seed, which is defined at the beginning of a run and does not change during it; three docking runs were performed for each protein-ligand pair and search space. The best docking poses found and affinities were similar between the runs in most cases, showing that the chosen parameters provided exhaustive search of the conformational space.

In the following sections the list of docking parameters and detailed docking results are provided. All coordinates are given in Å.

AChE (6H12)

Main active pocket

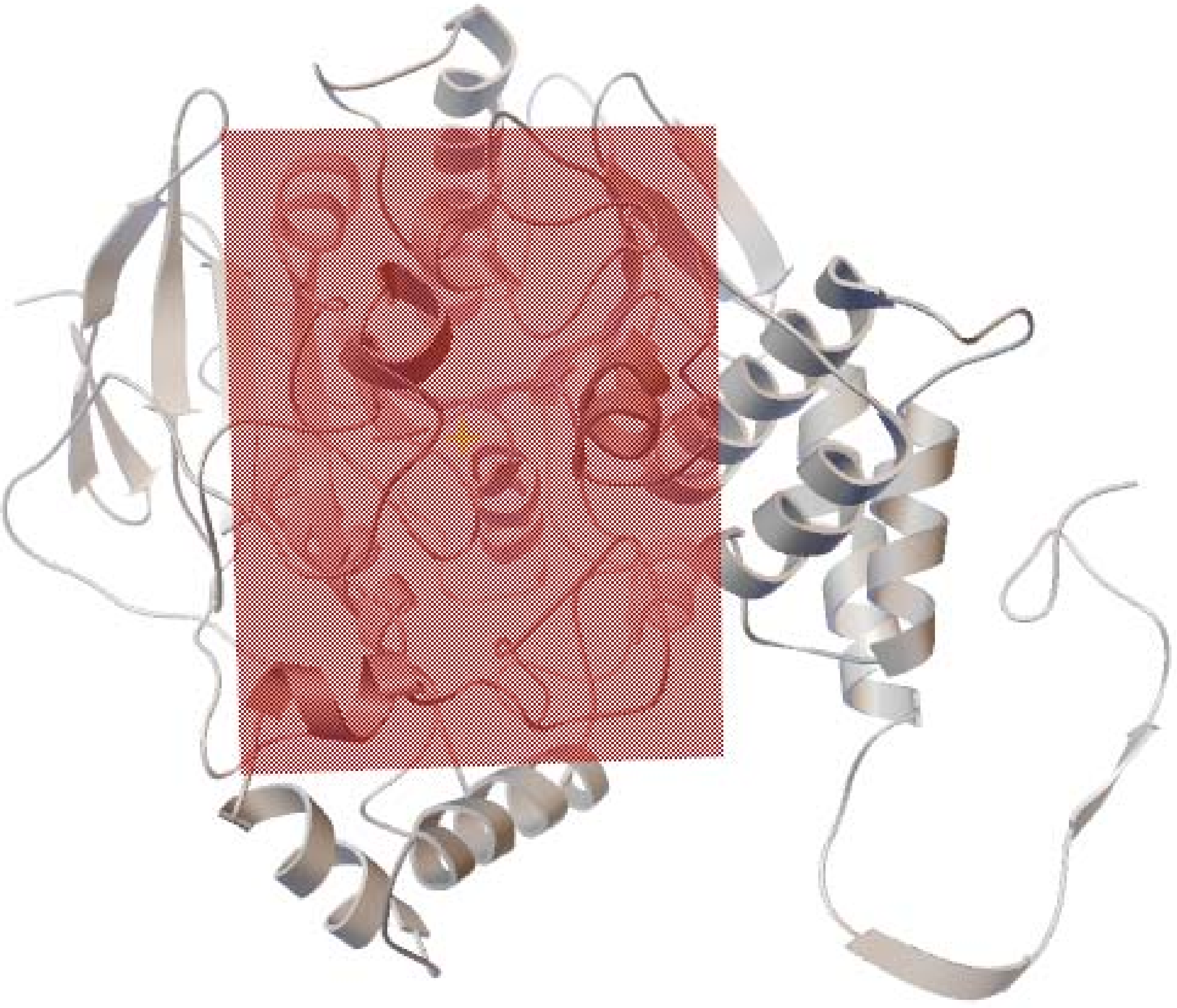

S31 Fig. Search space for the main pocket of 6H12.

Search space:

spacing 1.000

npts 26 34 26

center 80.286 214.252 66.066

Exhaustiveness 50.

Best affinities, kcal/mol:

- no water: neostigmine -7.6, 5-*N*-methylmaytenine -10.5, stepharine -10.3.;
- fully hydrated cavity according to the crystal structure: neostigmine -6.2, 5-*N*-methylmaytenine -8.6, stepharine -6.8;
- single water molecule screening: neostigmine -7.6, 5-*N*-methylmaytenine -10.5, stepharine -10.3.

Modeled loops

Model #1

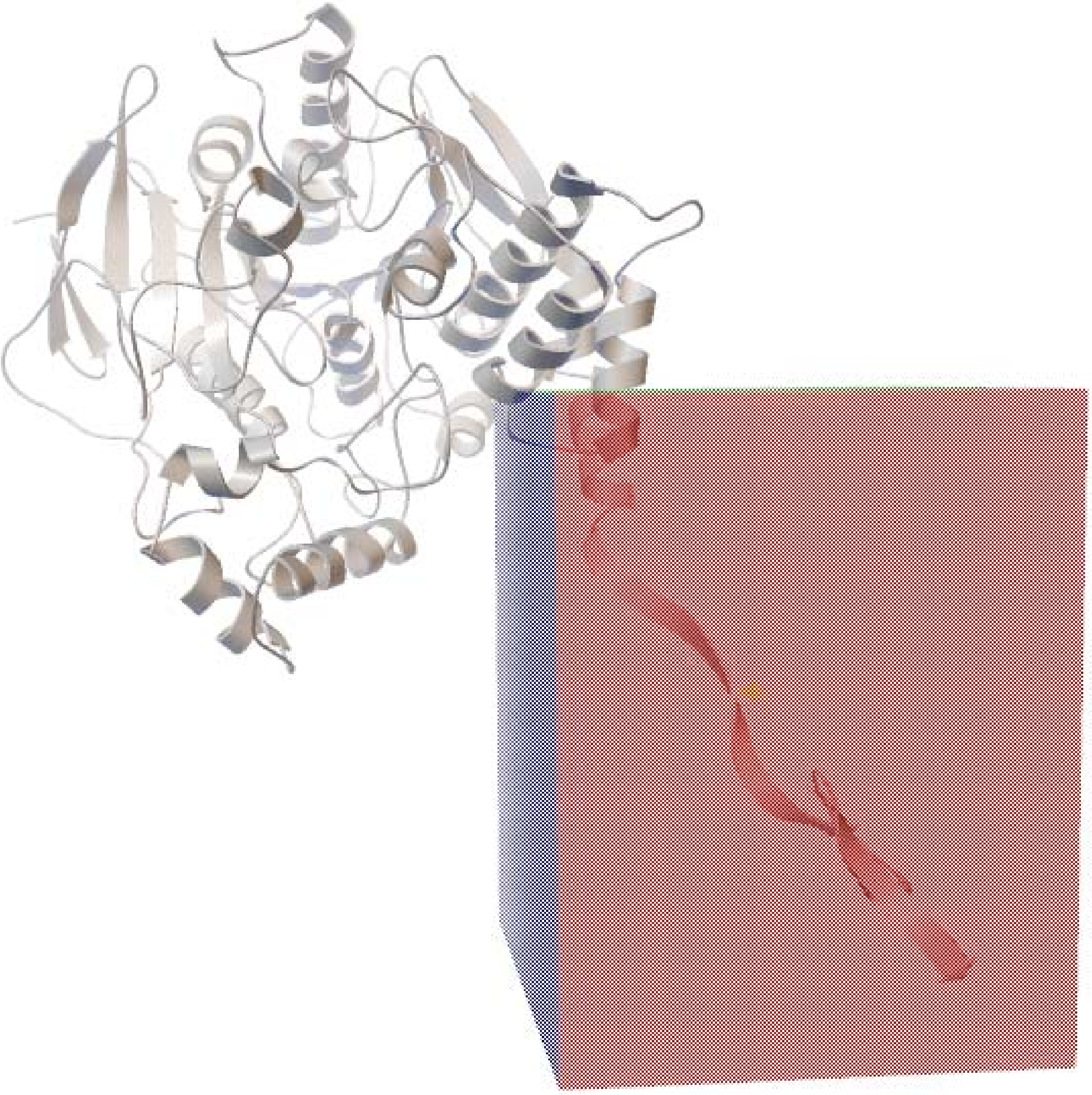

S32 Fig. Search space for the modeled loop #1 of 6H12.

Search space:

spacing 1.000

npts 44 52 40

center 76.258 178.974 103.710

Exhaustiveness 500.

Best affinities, kcal/mol:

- no water: neostigmine -4.4, 5-*N*-methylmaytenine -5.9, stepharine -5.5.

Model #2

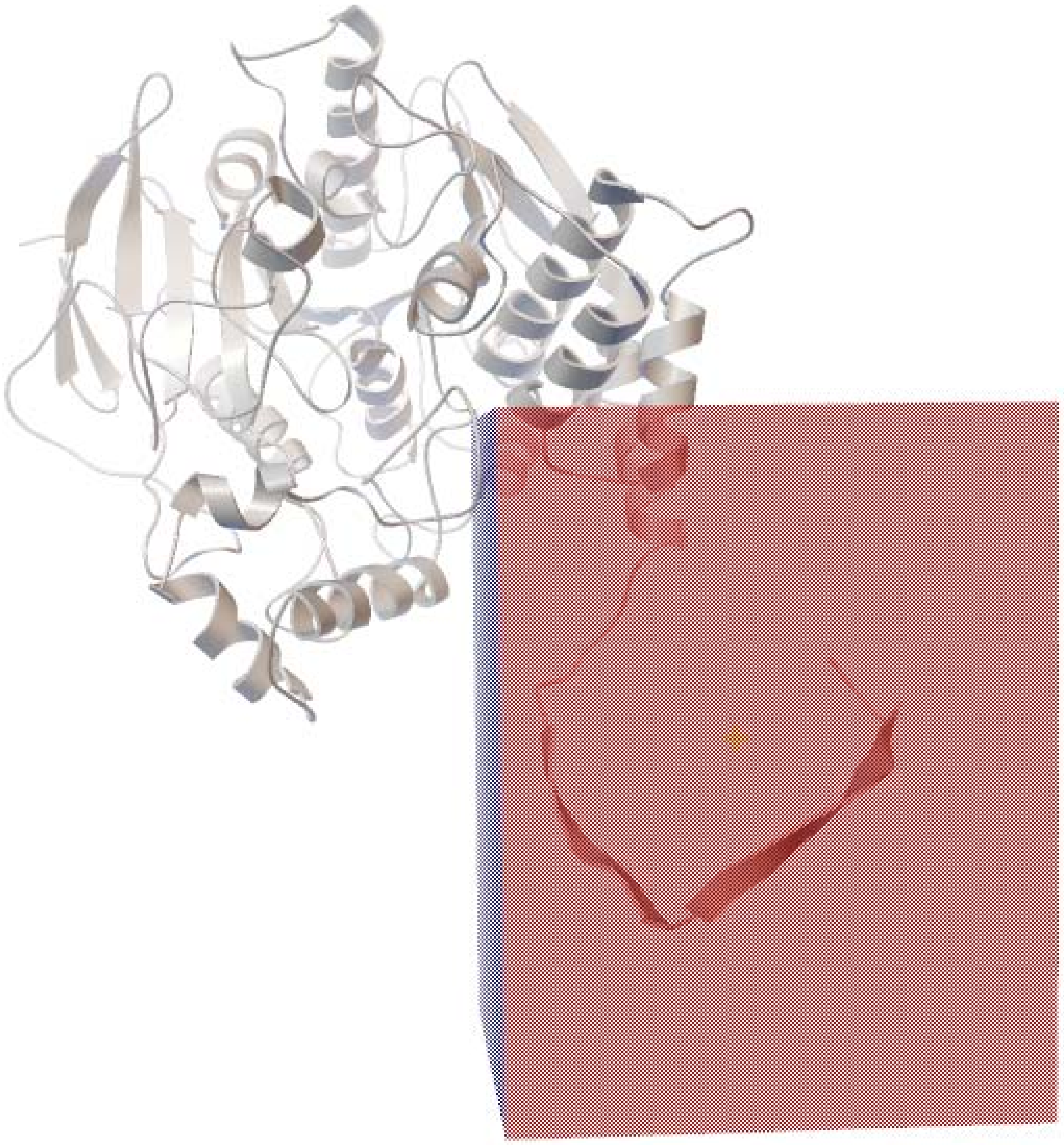

S33 Fig. Search space for the modeled loop #2 of 6H12.

Search space:

spacing 1.000

npts 38 52 40

center 77.258 178.974 98.009

Exhaustiveness 500.

Best affinities, kcal/mol:

- no water: neostigmine -4.6, 5-*N*-methylmaytenine -5.8, stepharine -6.3.

Model #3

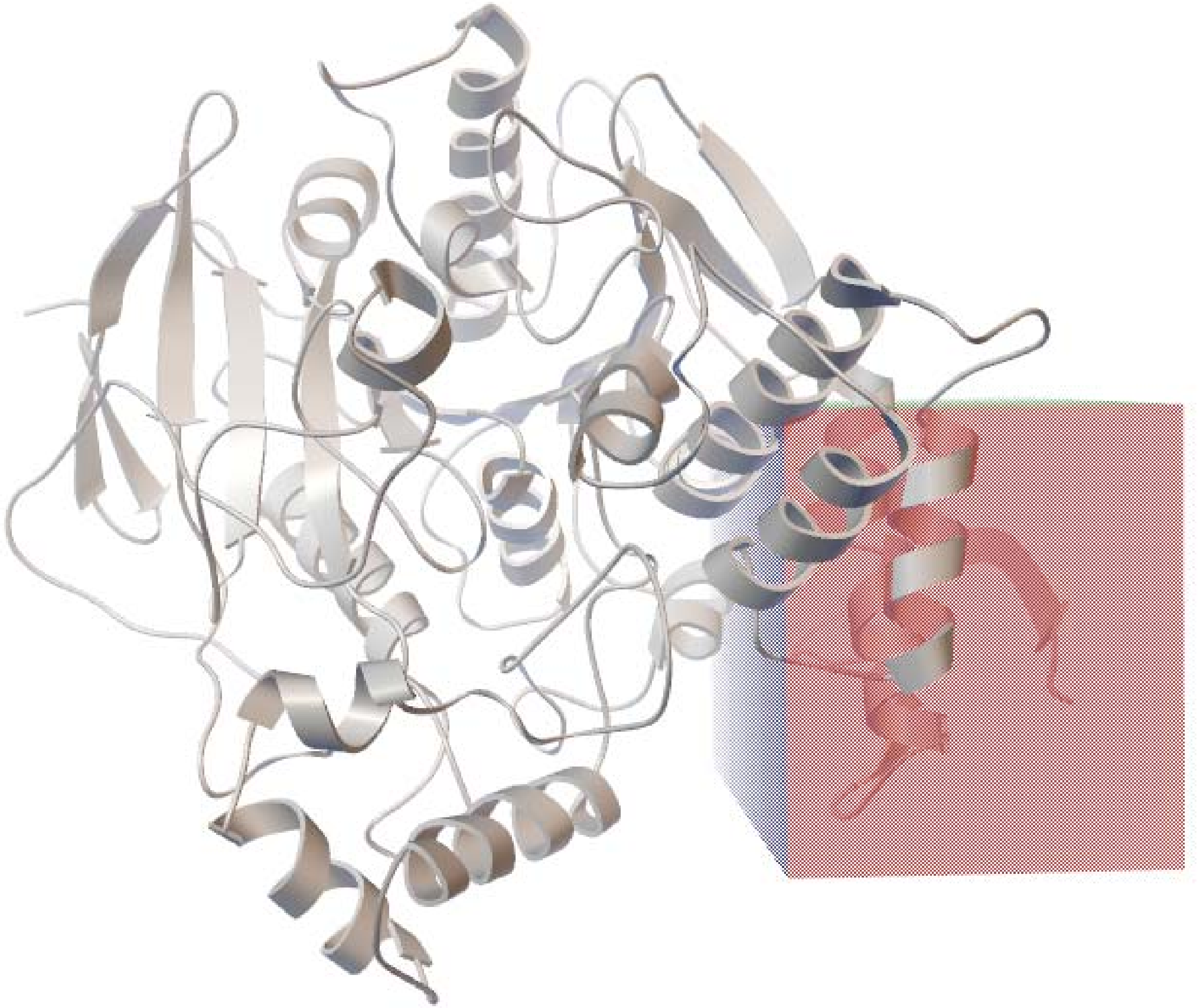

S34 Fig. Search space for the modeled loop #3 of 6H12.

Search space:

spacing 1.000

npts 48 28 24

center 108.598 201.514 97.257

Exhaustiveness 500.

Best affinities, kcal/mol:

- no water: neostigmine -5.6, 5-*N*-methylmaytenine -6.4, stepharine -7.2.

Model #4

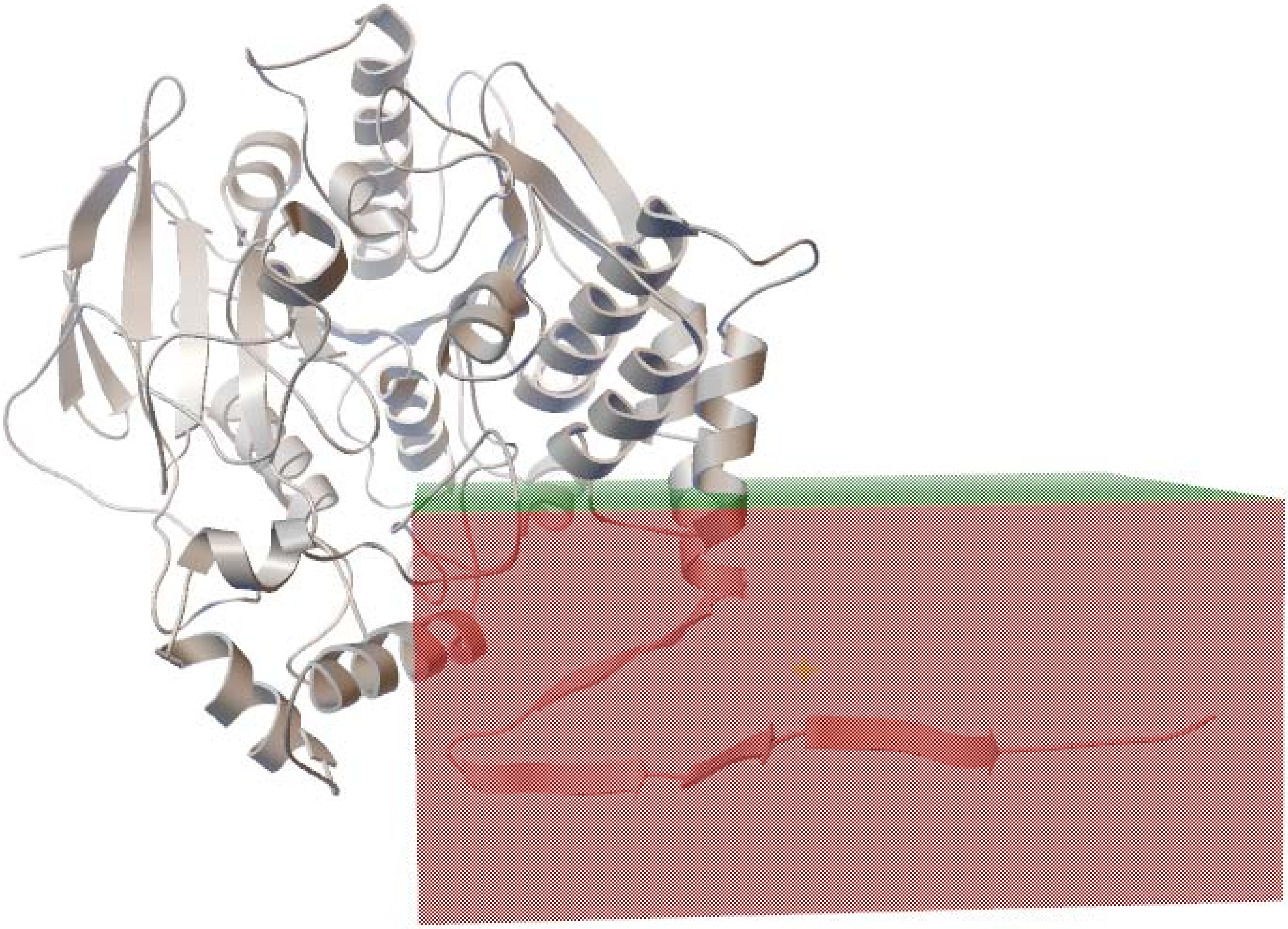

S35 Fig. Search space for the modeled loop #4 of 6H12.

Search space:

spacing 1.000

npts 42 28 60

center 89.254 188.441 100.397

Exhaustiveness 500.

Best affinities, kcal/mol:

- no water: neostigmine -4.9, 5-*N*-methylmaytenine -6.2, stepharine -6.6.

Model #5

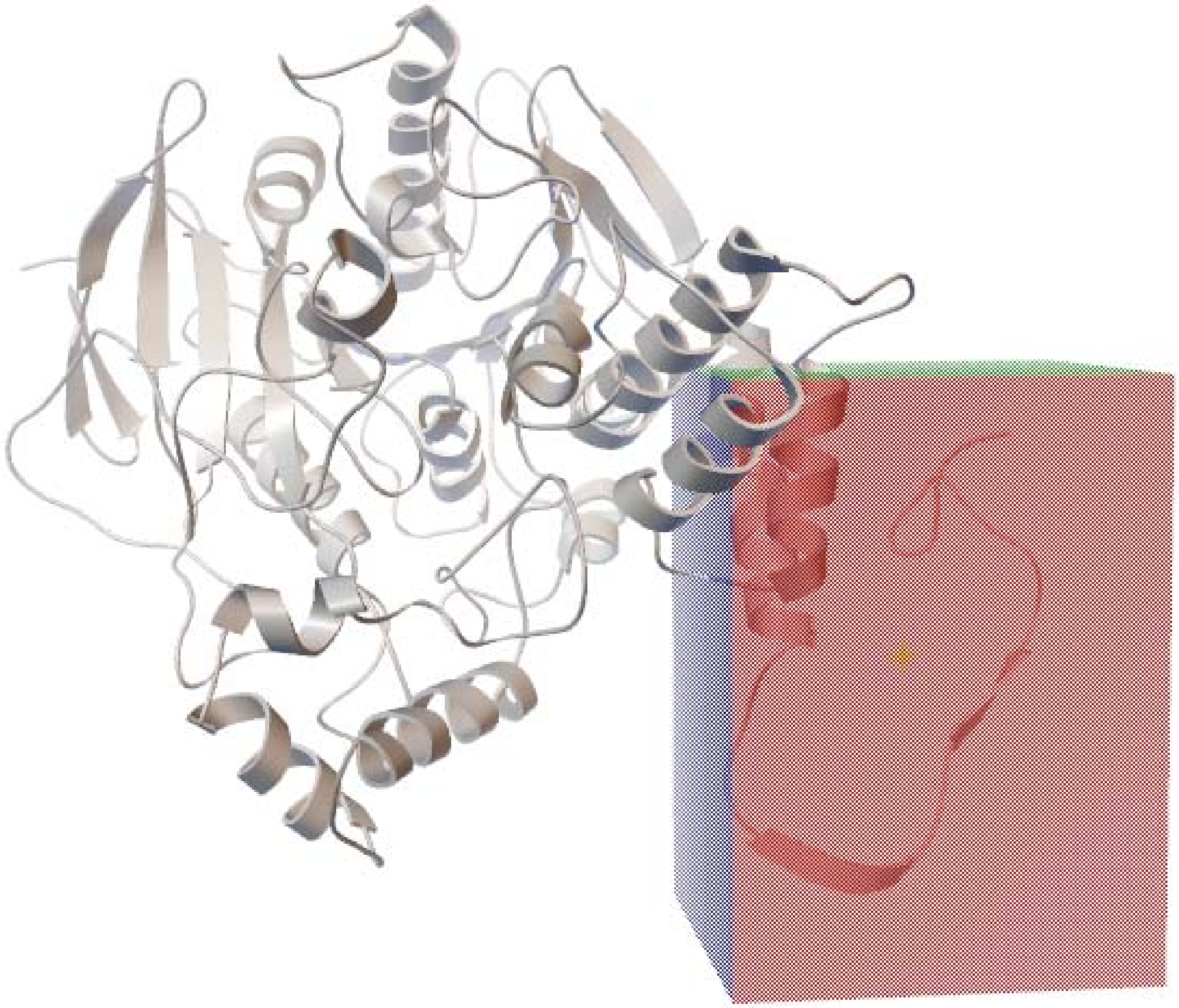

S36 Fig. Search space for the modeled loop #5 of 6H12.

Search space:

spacing 1.000

npts 26 42 30

center 90.842 193.329 102.425

Exhaustiveness 500.

Best affinities, kcal/mol:

- no water: neostigmine -5.0, 5-*N*-methylmaytenine -7.0, stepharine -7.2;
- single water molecule screening: neostigmine -5.0, 5-*N*-methylmaytenine -7.2, stepharine -7.2.

Rear region

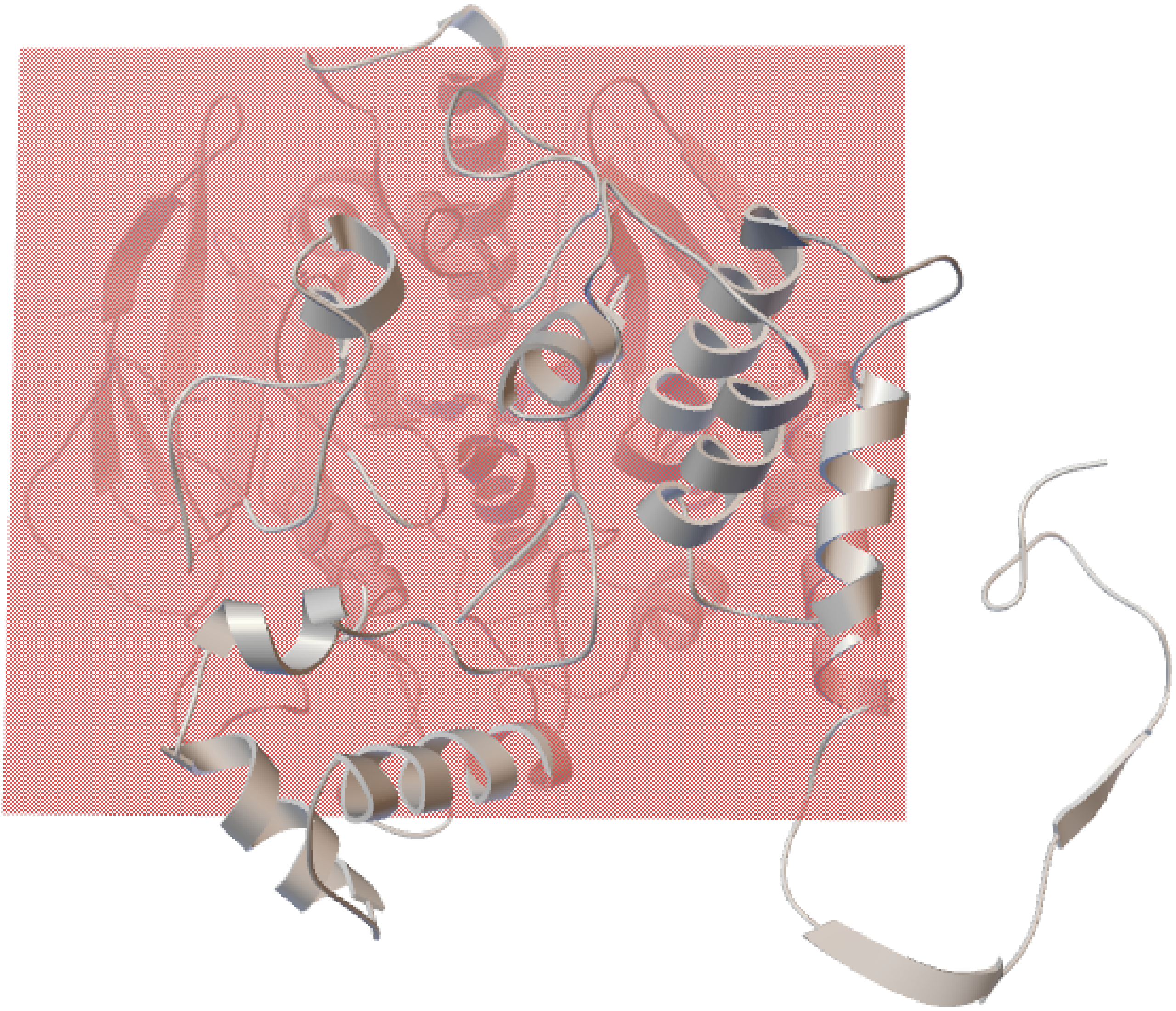

S37 Fig. Search space for the rear region of 6H12.

Search space:

spacing 1.000

npts 36 50 58

center 109.878 212.061 68.212

Exhaustiveness 500.

Best affinities, kcal/mol:

- no water: neostigmine -5.6, 5-*N*-methylmaytenine -6.3, stepharine -7.2.

IL-6 (4NI7)

Model #1

Search space covered the whole protein:

spacing 1.000

npts 46 40 48

center -4.977 12.904 9.694

Exhaustiveness 500.

Best affinities, kcal/mol:

- no water: 5-*N*-methylmaytenine -5.4, stepharine -6.6.

Model #2

Search space covered the whole protein:

spacing 1.000

npts 46 40 48

center -4.977 12.904 9.694

Exhaustiveness 500.

Best affinities, kcal/mol:

- no waters: 5-*N*-methylmaytenine -5.6, stepharine -6.6.

Model #3

Search space covered the whole protein:

spacing 1.000

npts 46 40 54

center -4.977 12.904 12.838

Exhaustiveness 500.

Best affinities, kcal/mol:

- no water: 5-*N*-methylmaytenine -7.9, stepharine -6.9;
- single water molecule screening: 5-*N*-methylmaytenine -7.5, stepharine -6.9.

IL-8 (3IL8)

Monomer

No water

Search space covered the whole protein:

spacing 1.000

npts 36 30 38

center 7.887 26.433 1.841

Exhaustiveness 500.

Best affinities, kcal/mol: 5-*N*-methylmaytenine -6.9, stepharine -5.9.

Water molecules screening

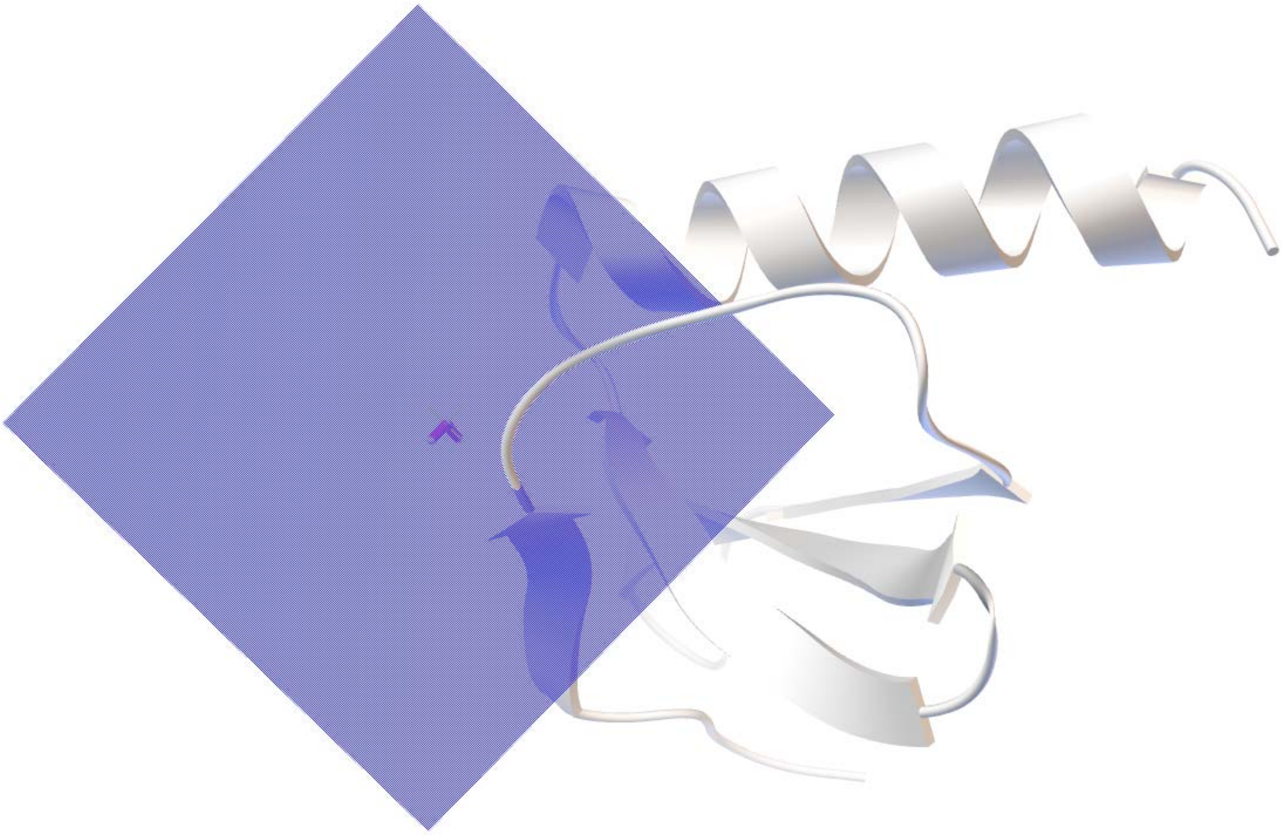

S38 Fig. Example of the search space for the single water molecule screening for 3IL8. Center of the search box (blue) is placed at the water molecule (purple).

Search:

spacing 1.000

npts 20 20 20

centered at the coordinates of the O atom of the water molecule

Exhaustiveness 50.

Best affinities, kcal/mol: 5-*N*-methylmaytenine -6.5, stepharine -5.6.

Dimer

No water

Search space covered the whole protein:

spacing 1.000

npts 40 42 42

center 12.373 21.430 0.000

Exhaustiveness 500.

Best affinities, kcal/mol:

- no water: 5-*N*-methylmaytenine -7.0, stepharine -5.9.

Water molecules screening

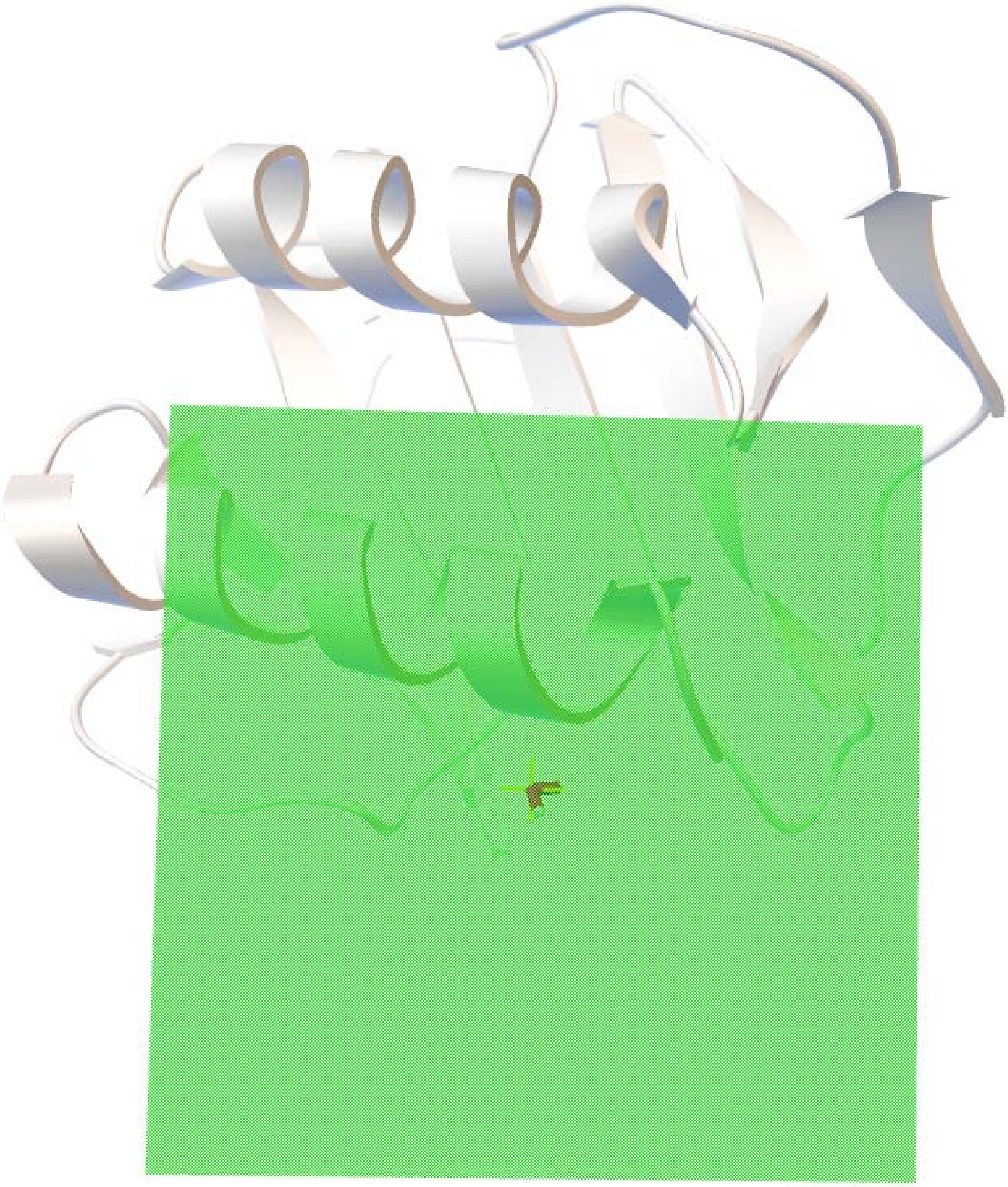

S39 Fig. Example of the search space for the single water molecule screening for the dimer of 3IL8. Center of the search box (green) is placed at the water molecule (purple).

Search:

spacing 1.000

npts 20 20 20

centered at the coordinates of the O atom of the water molecule

Exhaustiveness 50.

Best affinities, kcal/mol: 5-*N*-methylmaytenine -6.8, stepharine -5.9.

References

1. Hanwell MD, Curtis DE, Lonie DC, Vandermeersch T, Zurek E, Hutchison GR. Avogadro: an advanced semantic chemical editor, visualization, and analysis platform. J Cheminform. 2012;4: 17. doi:10.1186/1758-2946-4-17
2. Avogadro: an open-source molecular builder and visualization tool.
3. Stewart JJP. Optimization of parameters for semiempirical methods VI: More modifications to the NDDO approximations and re-optimization of parameters. J Mol Model. 2013;19: 1–32. doi:10.1007/s00894-012-1667-x
4. Stewart JJP. MOPAC2016. Stewart Computational Chemistry, Colorado Springs, CO, USA; 2016.
5. Morris GM, Huey R, Lindstrom W, Sanner MF, Belew RK, Goodsell DS, et al. AutoDock4 and AutoDockTools4: Automated docking with selective receptor flexibility. J Comput Chem. 2009;30: 2785–2791. doi:10.1002/jcc.21256
6. Sanner MF. Pyton: A Programming Language for Software Integration and Development. J Mol Graph Model. 1999;17: 57–61.
7. Dassault Systèmes BIOVIA, Discovery Studio Visualizer, v19. San Diego: Dassault Systèmes; 2018.
8. Šali A, Blundell TL. Comparative Protein Modelling by Satisfaction of Spatial Restraints. J Mol Biol. 1993;234: 779–815. doi:10.1006/jmbi.1993.1626
9. Pettersen EF, Goddard TD, Huang CC, Couch GS, Greenblatt DM, Meng EC, et al. UCSF Chimera?A visualization system for exploratory research and analysis. J Comput Chem. 2004;25: 1605–1612. doi:10.1002/jcc.20084
10. Trott O, Olson AJ. AutoDock Vina: Improving the speed and accuracy of docking with a new scoring function, efficient optimization, and multithreading. J Comput Chem. 2010;31: 455–461. doi:10.1002/jcc.21334
